# Proteochemometric Modeling Identifies Chemically Diverse Norepinephrine Transporter Inhibitors

**DOI:** 10.1021/acs.jcim.2c01645

**Published:** 2023-03-16

**Authors:** Brandon J. Bongers, Huub J. Sijben, Peter B. R. Hartog, Andrey Tarnovskiy, Adriaan P. IJzerman, Laura H. Heitman, Gerard J. P. van Westen

**Affiliations:** † Division of Drug Discovery and Safety, Leiden Academic Centre for Drug Research, Leiden University, Einsteinweg 55, Leiden 2333 CC, The Netherlands; ‡ Oncode Institute, Jaarbeursplein 6, Utrecht 3521 AL, The Netherlands; § Enamine Ltd, Chervonotkatska Street, 78, Kyiv 02094, Ukraine

## Abstract

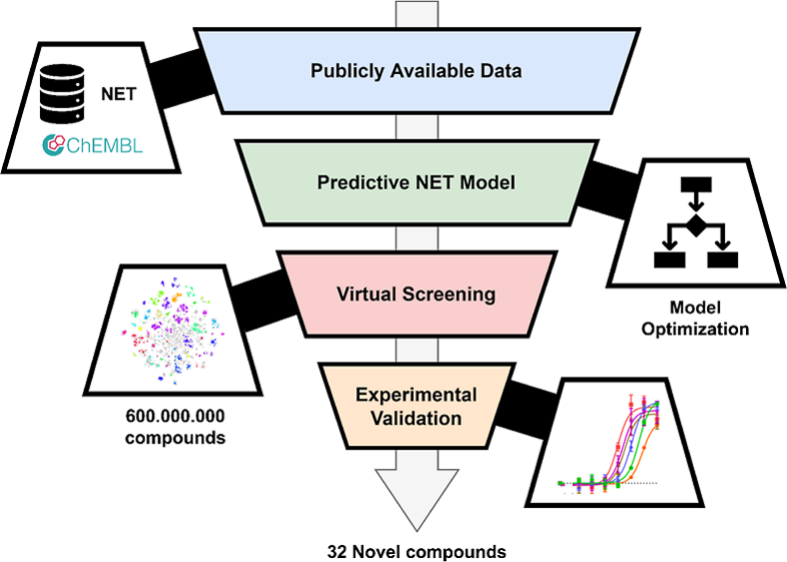

Solute carriers (SLCs) are relatively underexplored compared to other prominent protein families such as kinases and G protein-coupled receptors. However, proteins from the SLC family play an essential role in various diseases. One such SLC is the high-affinity norepinephrine transporter (NET/SLC6A2). In contrast to most other SLCs, the NET has been relatively well studied. However, the chemical space of known ligands has a low chemical diversity, making it challenging to identify chemically novel ligands. Here, a computational screening pipeline was developed to find new NET inhibitors. The approach increases the chemical space to model for NETs using the chemical space of related proteins that were selected utilizing similarity networks. Prior proteochemometric models added data from related proteins, but here we use a data-driven approach to select the optimal proteins to add to the modeled data set. After optimizing the data set, the proteochemometric model was optimized using stepwise feature selection. The final model was created using a two-step approach combining several proteochemometric machine learning models through stacking. This model was applied to the extensive virtual compound database of Enamine, from which the top predicted 22,000 of the 600 million virtual compounds were clustered to end up with 46 chemically diverse candidates. A subselection of 32 candidates was synthesized and subsequently tested using an impedance-based assay. There were five hit compounds identified (hit rate 16%) with sub-micromolar inhibitory potencies toward NET, which are promising for follow-up experimental research. This study demonstrates a data-driven approach to diversify known chemical space to identify novel ligands and is to our knowledge the first to select this set based on the sequence similarity of related targets.

## Introduction

Solute carriers (SLCs) are a divergent class of transporters and are understudied compared to other prominent receptor families, such as kinases and G protein-coupled receptors (GPCRs).^1^ However, SLCs can play a critical role in complex diseases and several SLCs are promising drug targets.^2−4^ To further characterize SLCs, the RESOLUTE consortium was founded to develop and distribute biochemical tools and assays for in vitro and in vivo studies of these transporters.^5^ SLC subfamilies recognize highly divergent natural substrates, and their sequence identity is low compared to the sequence identity in other superfamilies.^6^ Therefore, it is challenging to design family-wide studies, for example, kinome-wide studies,^7^ to find new ligands interacting with SLCs. Instead, the focus typically lies on single subfamilies, or even single SLCs, to identify novel compounds as promising candidates for SLC-related diseases.^1^

One such SLC-related disease is major depressive disorder, one of the leading causes of disability. An increasing trend in the worldwide incidence and prevalence of depression has been observed in recent years.^8,9^ Selective serotonin reuptake inhibitors, serotonin-norepinephrine reuptake inhibitors, and selective norepinephrine reuptake inhibitors are established classes of prescription drugs for the first-line treatment of depression that work by targeting SLCs.^10^ Although these drugs improve on the poly-pharmacological profile of tricyclic antidepressants that were used before, the current generation of reuptake inhibitors suffers from partial or nonresponsiveness, relatively low remission rates, slow onset of action, and risk of adverse effects.^11^ The norepinephrine transporter (NET/SLC6A2) is involved in the rapid reuptake of the neurotransmitter norepinephrine (NE) from the synaptic clefts of noradrenergic neurons in the peripheral and central nervous system.^12^ Thus, the identification of NET inhibitors could improve the efficacy of current antidepressants as well as provide scaffolds for alternative methods such as the development of (fluorescent) probes for in vitro imaging.^13^ Here, we aim to find these ligands using a combination of computational and wet lab experiments.

Computational studies, such as statistical modeling and ligand docking, have increased in popularity over the past decades. However, application to SLCs has been relatively limited.^14,15^ A 3D structure (crystal, cryo-EM, or homology-modeling based) of sufficient quality is required to perform structure-based drug discovery.^16^ However, the crystallization of SLCs is complex, given their membrane-bound nature, analogous to GPCRs. Therefore, only a limited number of structures are available for this protein family, limiting the ability to perform the structure-based design of ligands.^17,18^ While advances in cryo-EM and machine learning, such as AlphaFold, are expected to significantly increase the available structures and alleviate some of these issues, the application of AlphaFold in virtual screening remains to be demonstrated.^19−21^ In the absence of structural information, virtual screening can be performed using 2D chemical structures (ligand-based) or proteochemometric models (PCMs) that use both ligand and protein information.^22^ In both cases, machine learning is used to identify a correlation between bioactivity and structural features to screen for novel ligands.

Here, proteochemometric modeling and an impedance-based assay were applied to identify new chemotypes for NETs. While this transporter has been relatively well characterized compared to other SLCs, there is a need for novel ligands that effectively, efficiently, and selectively target NETs.^23,24^ For our PCM approach, we add proteins to the training data set based on sequence similarity. The novelty here is to use a data-driven selection method for the additional targets. This approach has not been reported in the literature to the best of our knowledge. However, it has been shown that expanding the data set with interaction data and protein information from related proteins leads to more predictive models, and it was expected that this equally applies to NETs.^25^ The optimal number of included proteins were sampled using similarity networks (SNs) and phylogenetic trees. PCMs were trained on the extended ligand space using publicly available bioactivity data from ChEMBL.^26^ The final model was subsequently applied to the extensive 600 million make-on-demand compounds in the Enamine REAL database. Finally, a subselection of candidates were synthesized and validated experimentally for sub-micromolar inhibitory potencies toward NETs with a hit rate of 5 out of 32 (16%), identifying diverse and novel chemotypes.

## Methods

### Computational Pipeline

**Figure 1:**
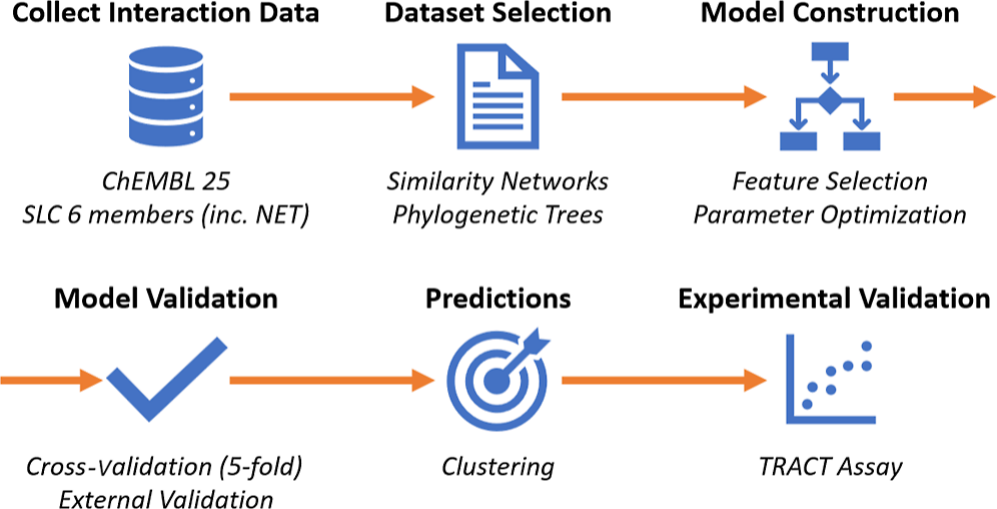
Schema for the sequential computational steps performed in this study. Data was collected from ChEMBL 25, fetching interaction data for NET and related SLC6 members. This data was then filtered for relevance with both SNs and phylogenetic trees to expand the data set with sequence information of the related proteins and the chemical space known for these proteins. The model was subsequently trained and optimized with feature selection and parameter optimization. Cross-validation was performed, alongside an external validation from a test set that was kept separate from optimization (10% of data) to ensure a minimal amount of overfitting. Subsequently, the Enamine database was virtually screened using the optimized model and predictions were clustered based on structural similarity. Finally, cluster centers were chosen for experimental validation via a TRACT assay.

An overview of the full computational pipeline is shown in Figure 1. This workflow can be applied to different targets to enrich the chemical space of a target of interest, provided the sequences and ligands with bioactivity measurements for these targets to be added are known. The steps are described below in further detail.

### Software

Proteochemometric modeling, data curation, feature extraction, and cluster analysis were performed in Pipeline Pilot (version 18^27^). Similarity network construction was done with Cytoscape (version 3.7.1^28^) in RStudio (version 3.6.0^29^). Any seeds used in randomization or model creation/prediction were set to “12345”.

### Bioactivity Data

Bioactivity data were gathered from the ChEMBL database (version 25.0^26^). A data point was defined as a combination of the chemical structure and protein target. Properties included per data point were canonical SMILES for the compound, the protein amino acid sequence of the target, and a pChEMBL value representing the affinity (in −log *M*). If there was more than one pChEMBL unit assigned to a data point, the leftmost of the following ranked units was chosen—*K*_i_ > IC_50_ > EC_50_ > *K*_d_. Duplicate pChEMBL values were averaged so that only a single bioactivity value for each interaction remained. Ultimately, 1,152,765 data points were collected for subsequent selection steps, containing 5,142 proteins and 622,007 compounds.

### Compound Standardization

Pipeline Pilot was used to convert canonical SMILES to structures. Compounds were standardized as done previously by Burggraaf et al.^30^ Steps included removing salts, standardizing stereoisomers/charges, and (de)protonation based on a pH of 7.0.

### Compound Descriptors

Physicochemical properties were calculated using Pipeline Pilot built-in components. Several fingerprints were calculated: estate keys/counts, MDL fingerprints, and a selection of extended-connectivity fingerprints.^31^ A full list of these compound descriptors can be found in Supporting Information Table S1. All these descriptors were used during the feature selection process to identify the optimal performing type.

### Protein Descriptors

Three classes of protein descriptors were tested. The first set of protein descriptors are alignment agonistic and were generated using the PROFEAT interface.^32^ Second, three alignment-based protein descriptors were included as used previously (Z-scales, FASGAI, and BLOSUM).^33^ Finally, a third set of protein descriptors was prepared using an in-house algorithm that samples a selection of protein descriptor generators and returns an autocross-correlated version.^34^ An overview can be found in Supporting Information Table S1. Like the compound descriptors, these were also used in the feature selection part of the process.

### Cross-Term Descriptors

No cross-term descriptors were calculated as it has been shown that these are not required when using nonlinear machine learning methods.^35,36^ Moreover, these cross-terms are generally poorly interpretable compared to chemical and/or protein descriptors. Hence, it was chosen to not use these here.

### Data Set Selection

Both SNs and phylogenetic tree formation were applied to filter the 5,142 available proteins from our initial data set to a relevant subset based on sequence similarity. As it has been shown that expanding the data set with interaction data and protein information from related proteins leads to more predictive models, it was expected that this equally applies to NET.^25^ Substrates of the family share similarities, yet compounds tested on non-NET proteins may not have been tested on the NET itself. addition of targets based on sequence information adds more chemically diverse compounds. The optimal data set from the created data sets based on the SNs and phylogenetic trees was identified using cross-validation. An “out-of-the-box” random forest model was trained on each set. The number of trees was set to 100, the number of descriptors to the square root for each layer, the minimum node size of 1, and no maximum depth for the layers. Then, internal cross-validation was performed in a 5-fold manner, with the *R*^2^ and residual mean-squared error (RMSE) reported for each model. Modeling performance on the data for both the similarity network-based approach as well as the phylogenetic tree-based approach was obtained using a 70/30 target-based data split using PCA-assisted K-means. The *R*^2^ and RMSE were then calculated using 10-fold cross-validation. The data set from the best-performing model was chosen for further follow-up.

### Similarity Networks

SNs were created using RStudio and package “Rcy3” in Cytoscape while displayed using “yFiles”. The full set of 5,142 proteins obtained from ChEMBL was used in the similarity investigations. Proteins were first analyzed using pBLAST, resulting in an all-*versus*-all similarity matrix. Networks were then created using a varying pBLAST threshold; a higher threshold resulted in a higher required similarity for inclusion and hence fewer proteins included for the network. Two networks represented the extremes and limits of the search space. These were a broad network (required similarity ≥ 100) representing multiple SLCs and a narrowed-down network (required similarity ≥ 800) containing only NET homologs.

### Phylogenetic Tree Formation

Phylogenetic trees were created using R packages “msa”, “seqinr”, and “ape”. Alignment was performed using the “msa” implementation of ClustalW. Phylogenetic tree formation ended at the largest network (pBLAST ≥ 100) possible within our available resources as a complete alignment was impossible for the full set of 5.142 proteins. Tree layers were created upward from the small network (pBLAST ≥ 800, NETs only), with each layer above it including the previous layer. Tree creation was stopped when it reached the large network (pBLAST ≥ 100).

### Data Set Pruning

Similarity clusters in the SN were iteratively trimmed by increasing the BLAST score threshold until proteins were separated from the cluster. Six separate clusters were formed from the iterative trimming at thresholds of 850, 650, 550, 350, and 100, again with 100 as the most inclusive and largest cluster. Similarly, a phylogenetic tree was constructed from the most extensive cluster of the SNs. The phylogenetic tree was pruned into subsets to contain progressively fewer proteins. These layers are numbered progressively up from the NET protein and were used to identify proteins similar to each other and NET.

### Model Construction

Models were constructed with three machine learning algorithms: random forest (RF, “ranger” package^37^), gradient boosting (GB, “xgboost” package^38^), and partial least squares (PLS, “pls” package^39^). For each model, optimization was performed for both the ligand and protein descriptors (feature selection) wherein the parameters for the model were found by grid-based parameter optimization (Table 1). Subsequently, stacking was added by running one or two of the algorithms to predict affinity (mean affinity plus standard deviation over 10-fold k-validation) for a given target. These predictions would form a new set of features for a secondary machine learning algorithm that would predict the affinity for the target based on the underlying model predictions. To check for potential overfitting on the data, predictions were performed on a 10% hold-out set. A list of descriptors is shown in Supporting Information Table S1. Optimal descriptors and parameters for each algorithm, as well as the final model, can be found in Supporting Information Table S2.

**Table 1:** Grids Used during the Parameter Optimization Procedure^a^

model	parameter grids	
random forest (ranger)	number of trees	100, 250, 500, 1000
	number of descriptors	Sqrt(D)*, Log2(D)*, fraction: 10%, 50%, 90%
	minimum node size	1, 5, 7
	maximum depth	5, 7, no max
gradient boosting (xgboost)	maximum number of trees	100, 250, 500, 1000
	learning rate	0.1, 0.3, 0.5
	gamma	0, 0.3, 0.5
	maximum depth	5,7
	data fraction	0.1, 0.5, 1.0
	descriptor fraction	0.5, 0.7
partial least squares (pls)	number of variables	100, 200, 300

a For different algorithms, different hyperparameters were sampled. Parameter grids are separated per model. *D represents the number of descriptors.

### Feature Selection

Stepwise feature selection was performed during model optimization in Pipeline Pilot using the “caret” package in R.^40,41^ At each step, the maximum number of iterations was set to 25 and the number of iterations without model improvement was set to 3. Model improvement was defined as an increase in 5-fold cross-validated *R*^2^. The resulting set of descriptors was deemed optimal for that specific type of model and was subsequently used in each model of that type.

### Hyperparameter Optimization

Hyperparameter optimization was performed using a full grid search. Model improvement was defined as an increase in 5-fold cross-validated *R*^2^. Once optimal hyperparameters were determined, these were subsequently used in each model of a given type. Parameter grids were separated per model as shown in Table 1.

### Model Validation

The robustness of our model was subsequently tested using a temporal split validation.^35^ The final data set after target selection and hyperparameter optimization (20,189 data points) was split into entries based on their year of publication according to ChEMBL. The training set contained known interactions before 2010 (15,106 data points), while the test set contained entries from 2010 (5,083 data points) and later as done previously in our lab.^35^

### Predicting and Clustering

Using the final model, predictions were performed on the Enamine REAL data set containing over 600 million compounds. A threshold was set at a predicted NET affinity of 100 nM (−log 7), and only compounds predicted with a better affinity were taken into consideration for follow-up. The resulting set of compounds was then further filtered based on chemical diversity using clustering based on the Tanimoto Similarity. As an additional step after clustering, an identity filter was applied that removed points with either a 90% or higher identity or a 50% or lower identity to compounds found in the training data. This filter ensured that compounds were novel compared to existing ligands, yet did not stray too far from known chemical space to influence model reliability. Clustering was performed using the R package “hdbscan”. Clusters were visualized in Pipeline Pilot. Finally, compounds were ranked within these clusters based on predicted NET affinity, and the top-ranked compound from each cluster was chosen for further experimental validation.

### Chemicals and Reagents

Jump In T-Rex HEK 293 cells modified for doxycycline-inducible overexpression of the wild-type human NET (JumpIn-NET) were provided by CeMM (Research Center for Molecular Medicine, Medical University of Vienna, Austria). JumpIn-NET cells were generated as described previously.^42^ Doxycycline hyclate was purchased from Sigma-Aldrich (St. Louis, MO, USA). Nisoxetine hydrochloride was purchased from Santa Cruz Biotechnology (Dallas, TX, USA). All other chemicals were of analytical grade and obtained from standard commercial sources.

### Cell Culture

JumpIn-NET cells were grown as adherent cells in the culture medium (high-glucose Dulbecco’s modified Eagle’s medium supplemented with 10% (v/v) fetal calf serum, 2 mM Glutamax, 100 IU/mL penicillin, and 100 μg/mL streptomycin) at 37 °C and 7% CO_2_. Cryopreserved cells were thawed and cultured for 1–2 passages in a culture medium. Cells were then cultured for up to 1 week in a culture medium supplemented with 2 mg/mL G418 and 5 μg/mL blasticidin before switching back to the culture medium at least 24 h prior to an experiment. Cell cultures were split twice per week at ratios of 1:8–1:16 in 10 cm plates.

### TRACT Assay

Label-free transport activity through receptor activation (TRACT) assays were performed using the xCELLigence real-time cell analysis (RTCA) platform as described previously.^42^ In short, cells grown on gold-coated electrodes of 96-well E-plates impede the electric current generated on the electrodes. Impedance is measured at 10 kHz and is converted to the dimensionless parameter Cell index (CI) using the following formula: CI = (*Z*_i_ – *Z*_0_) Ω/15 Ω, where *Z*_i_ is the impedance at any given time and *Z*_0_ is the baseline impedance measured at the start of each experiment.

Assays were performed at 37 °C and 5% CO_2_ in 96-well E-plates in a total volume of 100 μL. Background impedance was measured in a 40 μL culture medium. JumpIn-NET cells were seeded in 50 μL at 60,000 cells/well in the presence of 1 μg/mL doxycycline (or no doxycycline for the counter screen). The E-plate was left at room temperature for 30 min before placement in the recording station. Cells were grown for 22 h prior to inhibitor pretreatment. All compound additions were made using a VIAFLO 96 handheld electronic 96-channel pipette (INTEGRA Biosciences, Tokyo, Japan). After 22 h, cells were pretreated for 1 h with either a single concentration (single-point primary screen, 10 μM) or increasing concentrations (full-range concentration–inhibition curves, ranging from 10 pM to 10 μM) of compound or nisoxetine (positive control). Dilutions of compounds were first made in DMSO and then in phosphate-buffered saline (PBS). Vehicle-pretreated cells received only DMSO in PBS. Final amounts of DMSO were kept at 0.1% per well. After 1 h of inhibitor pretreatment, cells were stimulated with either vehicle or 1 μM norepinephrine in PBS containing 1 mM ascorbic acid (final concentration). Impedance was then measured every 15 s for 30 min.

### Data Analysis

Raw data from TRACT assays were recorded using RTCA Software v2.0 or v2.1.1 (ACEA Biosciences). CI values were normalized to the time point prior to substrate addition, obtaining normalized CI (nCI) values to analyze NE-induced cellular responses. Data were exported from RTCA Software and analyzed in GraphPad Prism v8.1.1 (GraphPad Software, San Diego, CA, USA). Per E-plate, nCI values of vehicle-pretreated and vehicle-stimulated cells were subtracted from all other data points to correct for any inhibitor and substrate-independent effects. NE-induced cellular responses were quantified by taking the net area under the curve (AUC) of the first 30 min after NE stimulation. Inhibitory potency (pIC_50_) values of compounds are reported as a concentration-dependent enhancement of the NE-induced response by fitting the AUC data with nonlinear regression to a sigmoidal concentration–inhibition curve with a fixed pseudo-Hill slope of 1. Data are shown as mean ± standard error of the mean (SEM) of three separate experiments each performed in duplicate.

## Results

### Data Set Pruning

**Figure 2:**
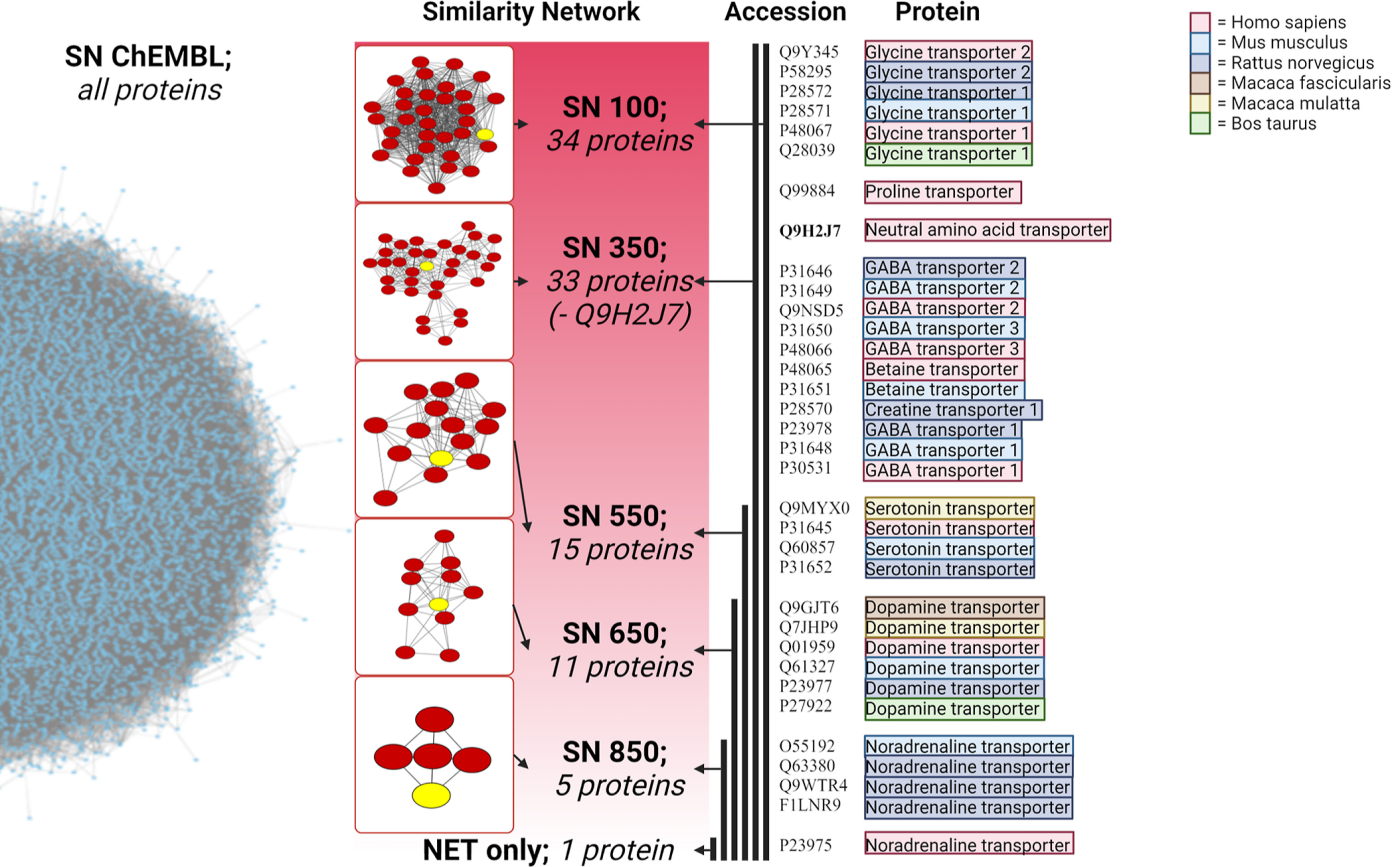
Sequence-based SNs obtained from SLCs in ChEMBL. Displayed are SNs wherein each node represents a single protein and each connection a pBLAST similarity above the chosen cutoff. A node in yellow denotes human NET. SN ChEMBL resulted in one large cluster of all proteins and was discarded (left hand). From there, the following thresholds were used for the SN SN100 (34 proteins), including NETs and related proteins from different species. SN350 (33 proteins), showing a smaller network with a section appearing to nearly dissociate from the main section. SN550 (15 proteins), containing the serotonin and dopamine transporters together with NETs. SN650 (11 proteins) serotonin was absent, and the minimum viable similarity network SN850 (and all SNs above this threshold) contains solely NETs from humans and other species.

**Figure 3:**
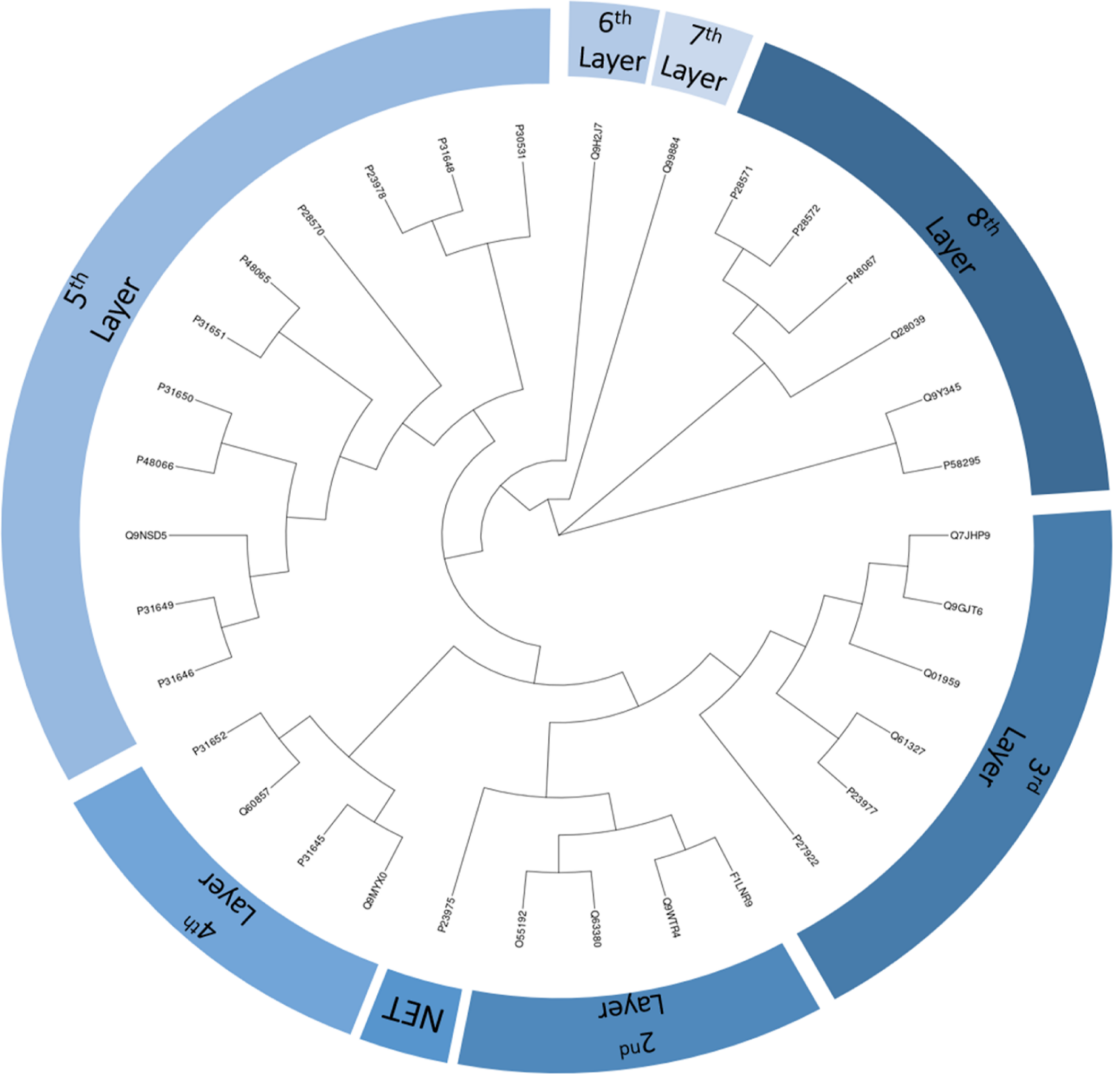
Phylogenetic tree of the maximally viable similarity network (SN100) reveals eight individual layers. Displayed is the phylogenetic tree of the proteins analyzed and colored with the various layers (defined as splits from the root of the tree defined by NET). This resulted in eight layers (including NETs as the first layer).

The full ChEMBL data set (5,142 proteins) had to be pruned as it was too large and contained too many proteins that were not related to NET. From the pruning, both a similarity network (Figure 2) and a phylogenetic tree (Figure 3) were created. Subsequently, the training data sets were formed by combining the chemical space from the proteins in the protein clusters of the SN or layers in the phylogenetic tree. Overlapping cluster-layer pairs were evaluated once, for example, SN850 (SN) overlapped with layer2 (phylogenetic), SN650 overlapped with layer3, SN550 overlapped with layer4, and SN100 overlapped with layer8.

### Final Data Set Selection

**Figure 4:**
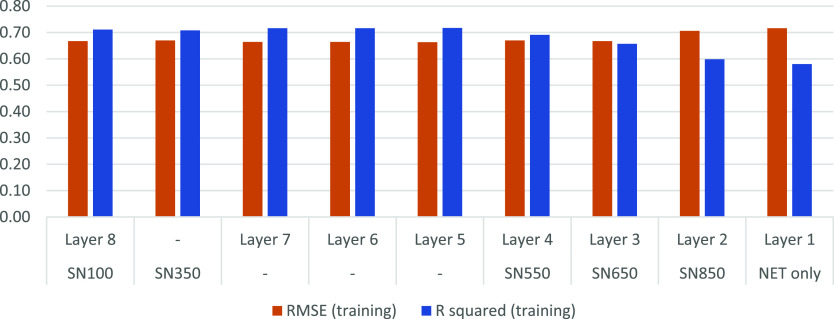
Differences in cross-validated *R*^2^ and RMSE from models trained on the different subsets. Displayed are the *R*^2^ and RMSE values generated during the data set selection process. A high value for *R*^2^ and a low value for RMSE were desired. SN100 was eventually preferred based on the obtained RMSE and *R*^2^ values combined with a larger size compared to the other sets.

The alignment methods resulted in nine extended “layers” for NET. Adding these chemical spaces to the NET data set was empirically tested to find the optimal training subset. To this end, an RF model was created and cross-validated to assess the *R*^2^ and RMSE (Figure 4). Subgroups layer5, layer6, layer7, SN350, and SN100 scored comparable, with an *R*^2^ of 0.71–0.72 and an RMSE of 0.66–0.67. The other sets scored worse, with an *R*^2^ of 0.58–0.62 and an RMSE of 0.66–0.75. Out of these five comparable sets, SN100 was selected as this contained the most data (20.189 data points) and produced top-performing models.

### Optimized Model Creation

**Figure 5:**
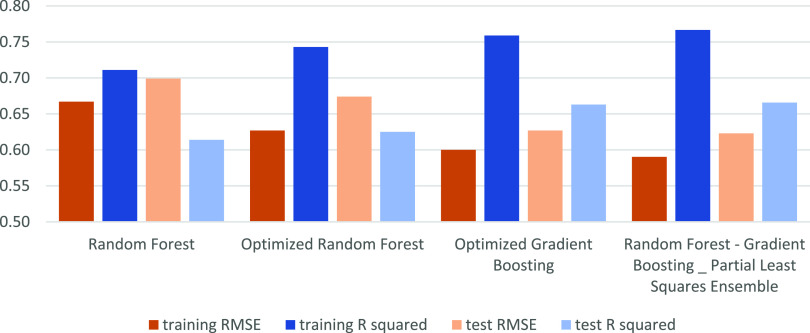
Overview of the performance of selected modeling approaches. Displayed are the internal (training, cross-validation) and external (testing 30% hold-out) statistics. Shown are three intermediate models, an RF model, an optimized RF model, and a GB model (left 3 bar sets); also shown is the final model consisting of an RF plus GB ensemble with a stacked PLS ensemble as the second step (right set of bars). At each step, model performance is improved.^25^

After selecting the optimal amount of proteins and chemical space connected thereto, the best ensembling approach was identified. Three different methods were used for base models: RF, GB, and PLS. These were subsequently tested in an ensemble approach. Finally, the best scoring model of each method was kept for further analysis using a 30% random-split hold-out set of NET interactions in the data set using the *R*^2^ and RMSE. Here, we will report the external (hold-out) set performance; cross-validation performance can be found in Figure 5. Due to the absence of cross-terms, PLS (*R*^2^: 0.28; RMSE: 0.93) underperformed compared to RF (*R*^2^: 0.61; RMSE: 0.70) and GB (*R*^2^: 0.65; RMSE: 0.62). Hence, the latter two were selected for continuation.

Next, stepwise feature selection and parameter optimization were used to fine-tune the models. Optimization of the RF and GB models showed a minor increase in *R*^2^ (final values of 0.62 and 0.66, respectively) and a decrease in RMSE for RF (0.67) (Figure 5). The performance further increased when PLS was stacked as a second model after the RF and GB models, which are used as additional descriptors. This combination performed the best (*R*^2^ 0.66 of and RMSE of 0.62) and will be referred to as the NET model from now on. The best-performing features and parameters can be found in Supporting Information Table S2. Finally, temporal split-based external validation was performed with ChEMBL data using the chosen model configuration, retrained without the temporal hold-out data (Supporting Information Figure S1). The external validation had an *R*^2^ of 0.24 and an RMSE of 1.02, worse than cross-validation but in line with previous examples of a temporal split.^35^ Based on the optimization and temporal validation and our prior experience with the expected performance of models trained on temporal split ChEMBL data, it was concluded that the NET model was robust enough to continue prospectively.

### NET Model Predicted 46 Groups of Compounds as Viable Candidates

**Figure 6:**
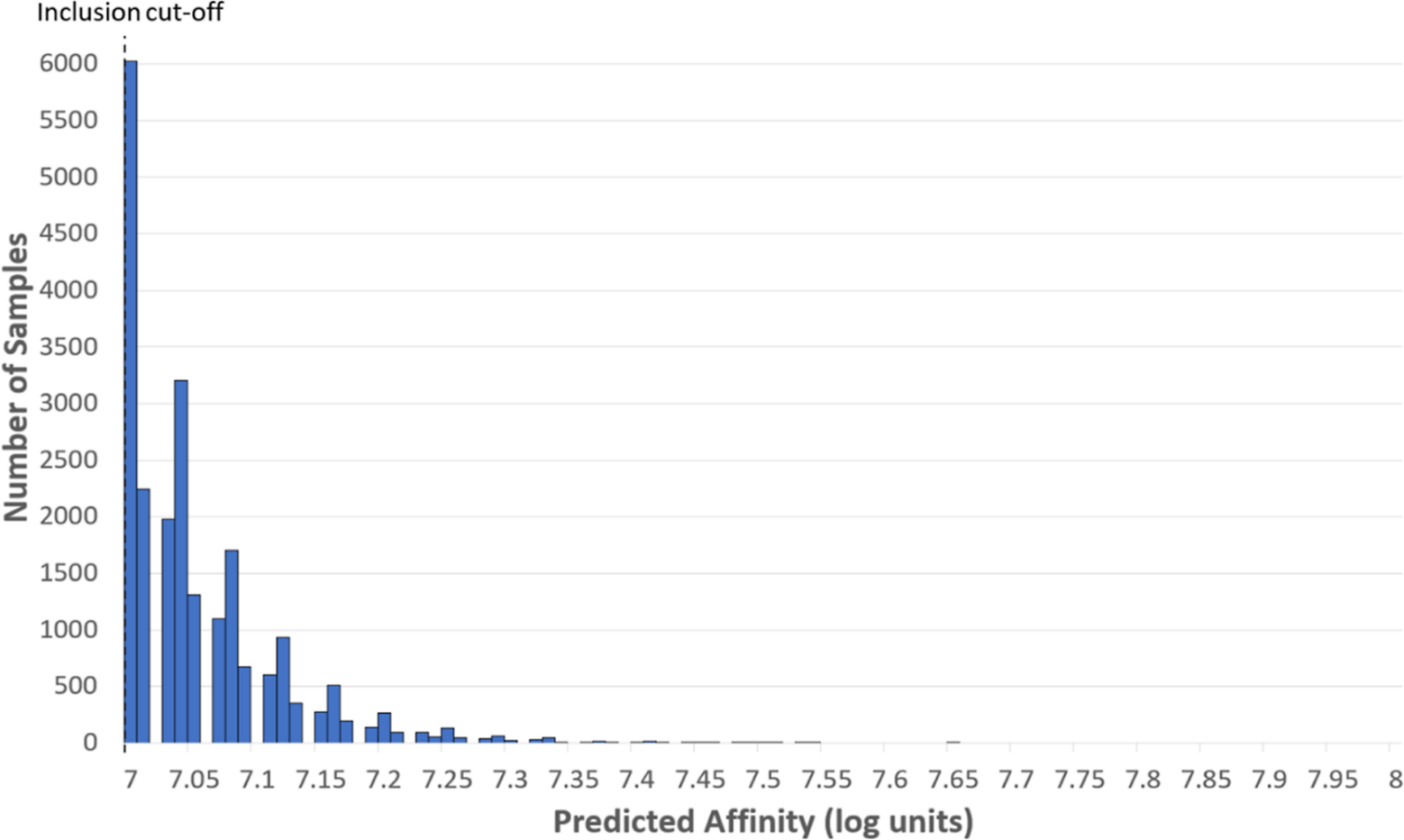
Distribution of all predictions with an affinity above 100 nM. Displayed is a histogram plot of the predicted affinities for the NET based on virtual screening of the Enamine compound database. Only molecules with a predicted affinity better than 100 nM were included (22,206 compounds).

The Enamine database was virtually screened with the optimized NET model to predict the bioactivity of these compounds for NETs. Subsequently, a final selection was made with filtering steps. Only compounds with a predicted affinity toward NETs better than 100 nM (7.00 log units) were considered (Figure 6). This threshold resulted in 22,206 compounds remaining, with the highest predicted affinity reaching 7.65 log units.

**Figure 7:**
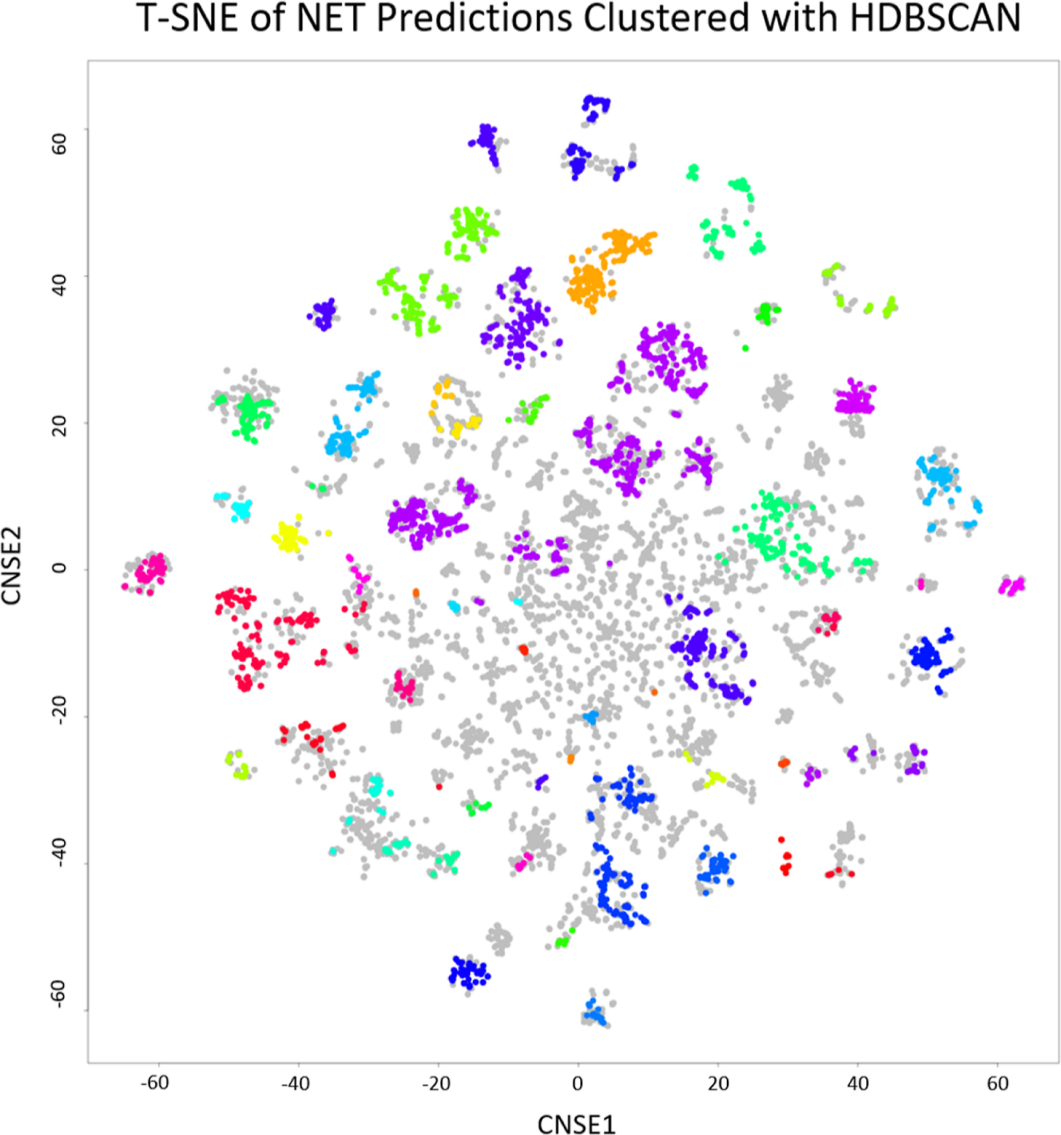
t-SNE of the 22.206 predictions with HDBSCAN designated clusters. The t-SNE displayed was created using 1024 bits of ECFP_6, and HDBSCAN shows 46 distinct clusters with different colors. Gray points were filtered out as they were deemed too similar (<90%) or too dissimilar (>50%) by HDBSCAN. The member of each cluster with the highest predicted activity was used as a representative of that cluster in the prospective validation.

Subsequently, the compounds were clustered using HDBSCAN on structural similarity and visualized with t-SNE using a 1024-bit ECFP-6 fingerprint (Figure 7) as we did previously.^43^ Compounds were first filtered (colored gray) by similarity to the training set, removing entries that either had a 90% or higher similarity or a 50% or lower similarity to the training set to ensure novelty and to stay within the applicability domain. The minimal amount of points in a cluster was set to 19, eliminating smaller clusters and resulting in 46 clusters remaining. Afterward, the compound with the highest predicted affinity within each cluster was selected for a final suggested list of 46 potential NET inhibitors. Of the 46 compounds, 32 could be obtained and tested for NET activity in a label-free impedance-based assay. The selected compounds are shown in Supporting Information Table S3 with analytical spectra available in the Supporting Information. The nearest training molecule based on Tanimoto Similarity is shown for each highest predicted molecule in Supporting Information Table S4. The average Tanimoto Similarity to the training set of these compounds is 0.38 ± 0.14.

### Experimental Validation

**Figure 8:**
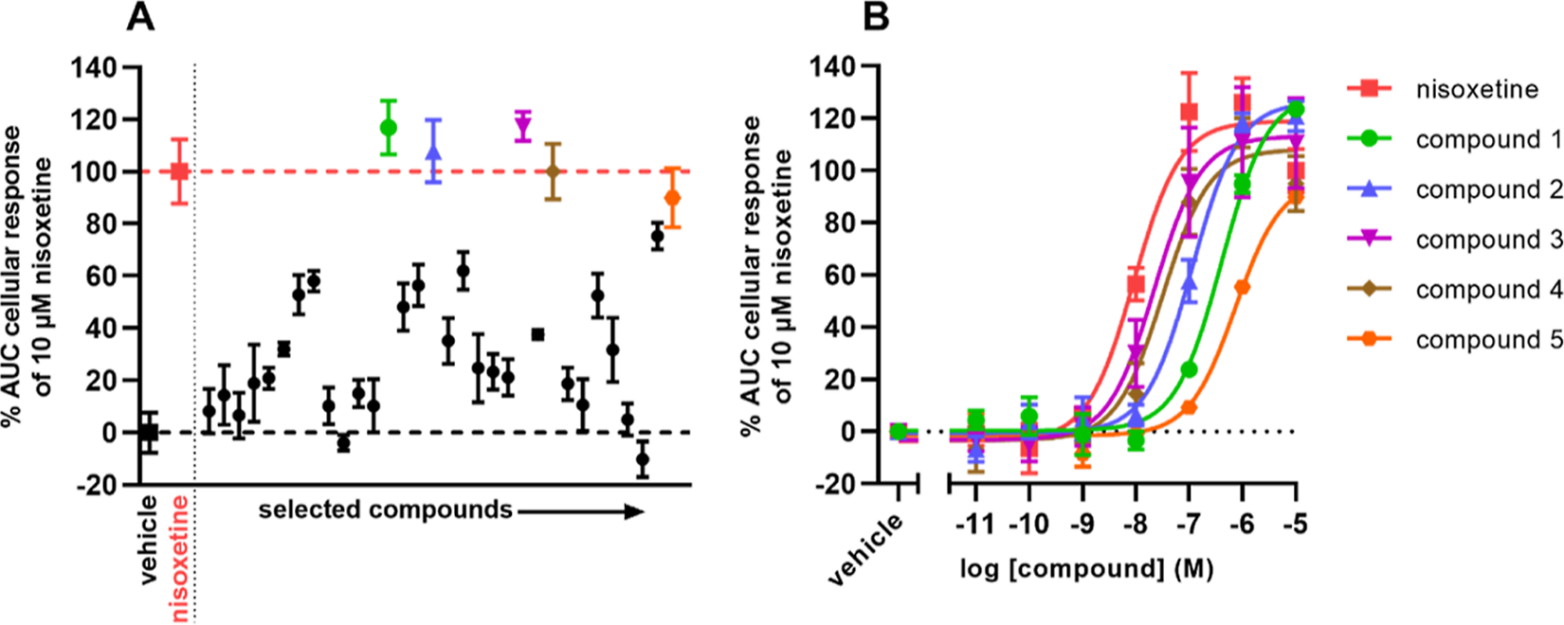
In vitro functional validation of hits in a label-free impedance-based TRACT assay. (a) Single-point screen of 32 hit compounds and (b) full-range concentration–inhibition curves of the top five compounds from the single-point screen. Doxycycline-induced JumpIn-NET cells were pretreated for 1 h with either vehicle or (a) 10 μM or (b) increasing concentrations of nisoxetine or hit compound. Subsequently, cells were stimulated with 1 μM NE and the CI was measured for 30 min. Cellular responses are expressed as the net AUC of the first 30 min after stimulation with NE. Data were normalized to the response of NE only (vehicle, 0%) and the response of NE in the presence of 10 μM nisoxetine (100%). Data are shown as the mean ± SEM of three separate experiments each performed in duplicate.

An impedance-based TRACT assay was performed to validate if predicted actives were biologically active.^42,44^ Here, a HEK293 cell line with inducible expression of NETs was used and the activation of endogenously expressed alpha-2 adrenergic receptors by NE was measured as a cellular response. A compound was considered a NET inhibitor if the compound was able to significantly enhance the NE-induced cellular response in a concentration-dependent manner. A single-point primary screen was performed with a 10 μM test compound, using the reference NET inhibitor nisoxetine as a positive control (Figure 8A; Supporting Information Table S5). Five of the 32 tested compounds were able to enhance the NE-induced response to a similar level as nisoxetine, indicating that the compounds inhibited NETs with high potency. None of the five compounds showed modulation of the NE response in cells lacking NETs (Supporting Information Figure S2), confirming that the enhanced NE-induced response was specific to NETs. To further characterize the most potent inhibitors, full-range concentration–inhibition curves were obtained for the top five compounds and inhibitory potency (pIC_50_) values were determined (Figure 8B; Table 2). The compounds on their own did not induce substantial cellular responses during pretreatment (Supporting Information Figure S3). All tested compounds showed concentration-dependent enhancement of the NE response with sub-micromolar inhibitory potencies (Figure 8B; Supporting Information Figure S5). Compounds 3 and 4 showed the highest pIC_50_ values (7.6 ± 0.1 and 7.5 ± 0.2, respectively), which were in the range of the pIC_50_ of nisoxetine (8.0 ± 0.0). These results demonstrate that at least five of the 32 tested compounds were biologically active NET inhibitors in a label-free TRACT assay.

**Table 2:**
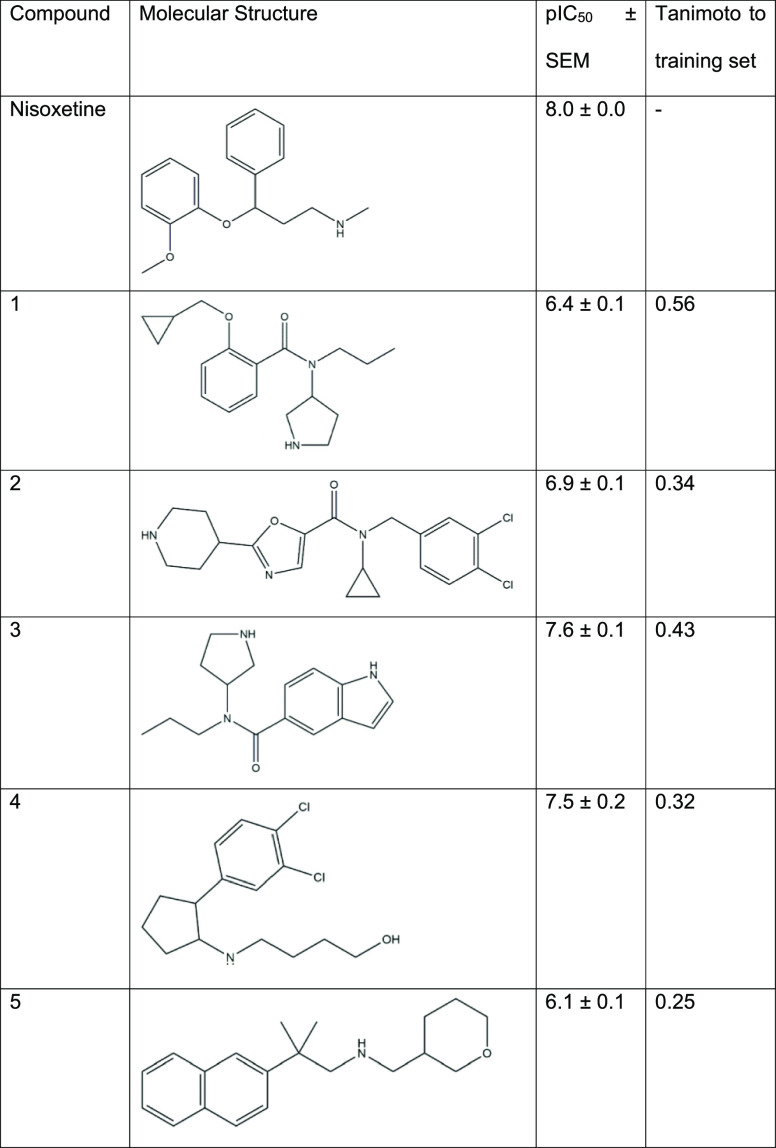
Inhibitory Potency (pIC_50_) Values of Tested Compounds as Determined in the Impedance-Based TRACT Assay^a^

a Data are reported as the mean ± SEM of three individual experiments, each performed in duplicate. Tanimoto similarity was calculated using ECFP_6 fingerprints.

## Discussion

In this study, an ML model that can identify novel inhibitors for human NETs was developed and validated. After the virtual screening of the Enamine database with this model, 46 diverse compounds were selected using clustering that were predicted to be highly active. Subsequently, these were submitted for experimental validation using a live cell, impedance-based TRACT assay. In the end, five novel inhibitors for NETs were identified.

### Data Selection

Here, we introduce a method to determine an optimal set of related proteins to include in a PCM, improving performance over single target models due to the inclusion of more data.^45^ Prior work in the area has primarily focused on small conserved families or very large protein superfamilies, and the ability to tune target inclusion as a parameter offers new possibilities.^36,46^ The optimal number of included similar sequences depends on the similarity, chemical diversity, and the number of data points per target. Therefore, the optimal amount is data set-dependent and should be optimized rather than giving a guideline.

ML models generally increase performance using more or better-quality data. In this research, ChEMBL version 25 was used, but recently, a comprehensive data set called Papyrus was released. Papyrus combines several data sets and is annotated and standardized for compatibility.^43^ Future work could benefit from using this larger set of bioactivity data and the inclusion of the experimental results here in the training set.

### Optimization of the Models

After optimizing our prediction models using R^2^ and RMSE, it was concluded that the ensemble-stacking model that combined all three methods performed the best. A stacking model was implemented using a combination of the RF and GB activity predictions that formed descriptors for a PLS model. However, *R*^2^ and RMSE values obtained from different combinations of these methods were very close. In the end, an ensemble-stacking model was used as earlier work concluded that these models tend to work better compared to single models.^35^ As demonstrated in our earlier work, deep learning could improve our model even further, but this will likely require the use of more data.^47^

### Similarity Networks

Phylogenetic trees and SNs were created to identify the optimal selection of proteins. In previously published comparisons between SNs and tree-based approaches, often used in metabolic pathway studies, both Oh et al. and Zhou et al.^48,49^ concluded that phylogenetic trees were less adaptable than the networks due to the inability to tune the threshold in trees. For networks, however, this similarity threshold can be tuned, as shown by changing the pBLAST score threshold, which allowed variation of the data set.^33,34^ SNs have also been used in similar research, for example, to visualize enzyme function using the protein sequence, to visualize relationships between protein superfamilies, or to find similarities using gene ontology databases.^50−52^ Applying insights from these studies to the networks could create a higher-quality network. Moreover, the here-introduced approach can be the subject of follow-up work to test different methods than SNs or phylogenetic trees to select related targets.

### NET Inhibitor Candidates’ Selection

For clustering, only compounds with a predicted affinity of 100 nM or better were included (resulting in a set of 22,206 compounds). Lowering that threshold required clustering that was too computationally expensive, and the interest was in finding novel high-affinity ligands. In follow-up work, other dimensionality reduction methods or an increase in computational power could reveal other promising chemical clusters. In addition, exploring analogues of the selected inhibitors and 14 cut candidates or centers from the smaller clusters could lead to additional hits.

### Experimental Validation

After clustering, 32 compounds were screened for their activity on NETs using an impedance-based TRACT assay.^42,44^ 11 out of the 32 compounds displayed more than 50% enhancement of the NE-induced response at 10 μM, which is substantial considering that these compounds are structurally distinct from known ligands. This was also apparent from the five hit compounds, which all display sub-micromolar potencies toward NETs. Although all compounds contain structural elements that are key to interacting with the sub-pockets of the norepinephrine binding site, such as a secondary amine and a substituted aromatic moiety, the scaffolds vary significantly in the substitution and size of aliphatic groups or the presence of an amide moiety (Table 2).^53^ Moreover, the hits had an average Tanimoto similarity of 0.38 ± 0.12 to the training set, confirming their novelty. Thus, these scaffolds could provide a starting point for the design and synthesis of derivatives, quantitative structure–activity relationships, and subsequent hit optimizations of novel NET inhibitors.

## Conclusions

Here, we introduce a method to identify novel protein inhibitors using a combination of machine learning techniques. In contrast to prior work, an optimal set of related targets for the PCM were determined dynamically based on data analysis and subsequent modeling. To the best of our knowledge, dynamically determining the optimal number of related proteins has never been done in a PCM setting. This approach was then applied to identify novel NET inhibitors, which were found by virtually screening a database containing virtual molecules that were synthesized on demand. From this screen, 32 compounds were ordered and 5 out of 32 compounds (16% hit rate) showed a similar affinity as the reference high-affinity NET inhibitor nisoxetine. Moreover, 11 out of 32 (34%) displayed >50% inhibition in our single-point screen at 10 μM.

## Supporting Information Available

Predicted pChEMBL values of temporal split correlated with the observed values, counter screen of the five hit compounds in a label-free impedance-based TRACT assay, representative xCELLigence traces of JumpIn-NET cells during inhibitor pretreatment in a label-free impedance-based TRACT assay, representative xCELLigence traces of JumpIn-NET cells during NE stimulation in a label-free impedance-based TRACT assay, molecular and protein descriptors used throughout the model building process, optimal settings obtained from stepwise feature selection and grid parameter optimization, cluster centers that were chosen for experimental follow-up, selected cluster compounds and the nearest structurally similar compound in the training set, single-point screen compounds and their inhibitory activity at 10 μM, and analytical spectra for the 32 synthesized compounds selected for experimental follow-up (PDF)

## Notes

The code, the training sets, as well as the consolidated predictions made on the Enamine set, needed to reproduce the conclusions of this study, are available on the following open 4TU repository: https://doi.org/10.4121/19618068. Additionally, the code used for creating the model can be found at https://github.com/CDDLeiden/NETPredictions. The full set of raw predictions can be shared upon request (∼60 GB). Proteochemometric modeling, data curation, feature extraction, and cluster analysis were performed in Pipeline Pilot (version 18^27^). Similarity network construction was done with Cytoscape (version 3.7.1^28^) in RStudio (version 3.6.0^29^). Bioactivity data were gathered from the ChEMBL database (version 25.0^26^).
