# Supplementary material for: Proteochemometric Modeling Identifies Chemically Diverse Norepinephrine Transporter Inhibitors
Source: J Chem Inf Model. 2023 Mar 16;63(6):1745–55. doi: 10.1021/acs.jcim.2c01645 (PMC10052348; doi:10.1021/acs.jcim.2c01645)
Supplement: Supplementary file 1 — ci2c01645_si_001.pdf [file ci2c01645_si_001.pdf]

Supporting Information

Proteochemometric Modeling Identifies Chemically Diverse Norepinephrine Transporter Inhibitors

B.J. Bongers[1†], H.J. Sijben[1†], P.B.R. Hartog[1], A. Tarnovskiy[3] A.P. IJzerman[1], L.H. Heitman[1,2], G.J.P van Westen[1].

[1] Division of Drug Discovery and Safety, Leiden Academic Centre for Drug Research, Leiden University, Wassenaarseweg 76, 2333 AL Leiden, The Netherlands

[2] Oncode Institute, Jaarbeursplein 6, 3521 AL Utrecht, The Netherlands

[3] Enamine Ltd., Chervonotkatska St, 67, Kyiv, Ukraine, 02094

† These authors contributed equally

Corresponding author is G.J.P. van Westen (gerard@lacdr.leidenuniv.nl)

Table of Contents	Page
Figure S1: Predicted pChEMBL values of temporal split correlated to the observed values.	2
Figure S2: Counter screen of the five hit compounds in a label-free impedance-based TRACT assay.	3
Figure S3: Representative xCELLigence traces of JumpIn-NET cells during inhibitor pretreatment in a label-free impedance-based TRACT assay.	4
Figure S4: Representative xCELLigence traces of JumpIn-NET cells during norepinephrine (NE) stimulation in a label-free impedance-based TRACT assay.	5
Table S1: Molecular and Protein descriptors used throughout the model building process.	6
Table S2: Optimal settings obtained from stepwise feature selection and grid parameter optimization.	7
Table S3: Cluster centers that were chosen for experimental follow-up.	8
Table S4: Selected cluster compounds and nearest structurally similar compound in the training set.	10
Table S5: Single Point screen compounds and their inhibitory activity at 10 µM.	22
Analytical spectra for the 32 synthesized compounds selected for experimental follow-up.	28

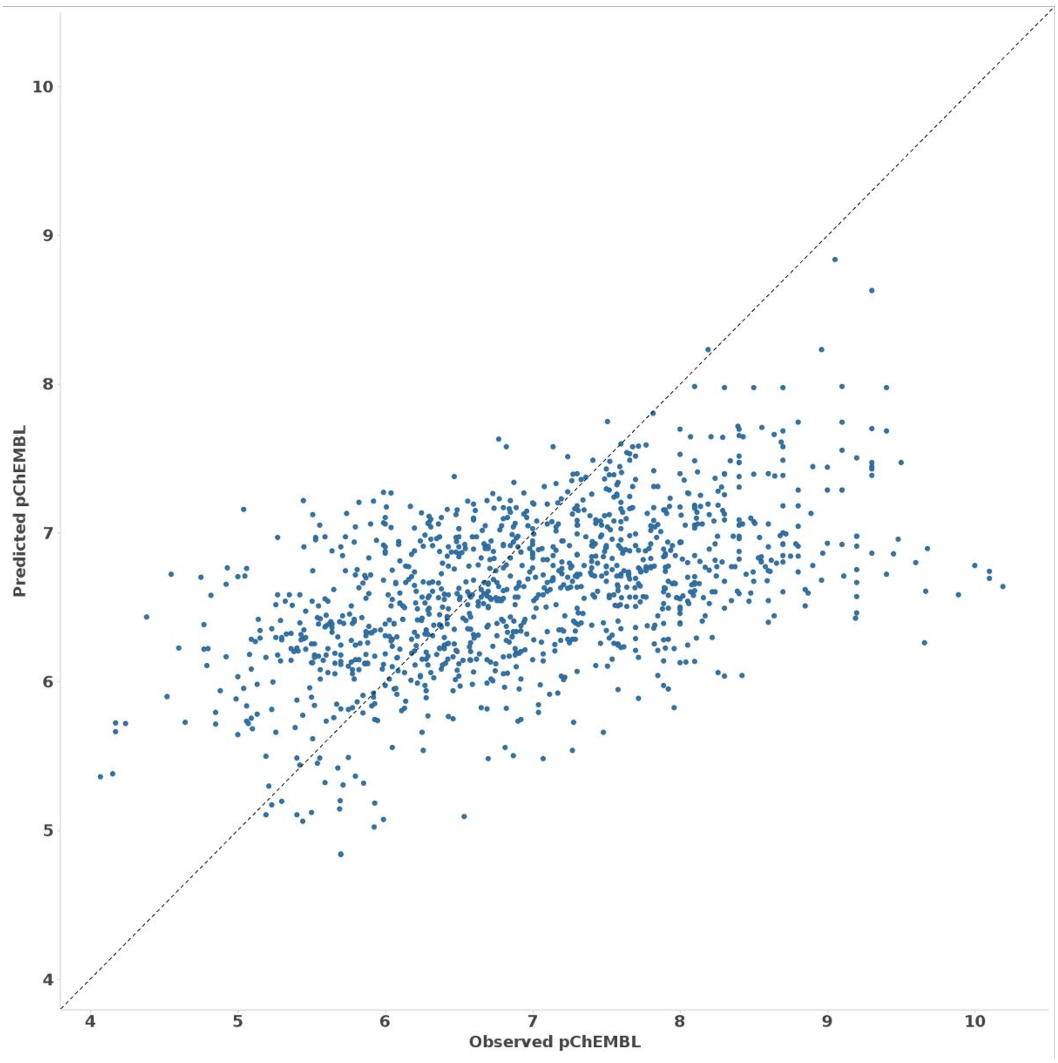

Figure S1. Predicted pChEMBL values of temporal split correlated to the observed values. Temporal split prediction where all known interactions of ChEMBL of 2010 and before were used as training set (15.106 data points) and those of 2011 and later (5.083 data points) were used as the test set. R^2 was 0.24 and RMSE was 1.02.

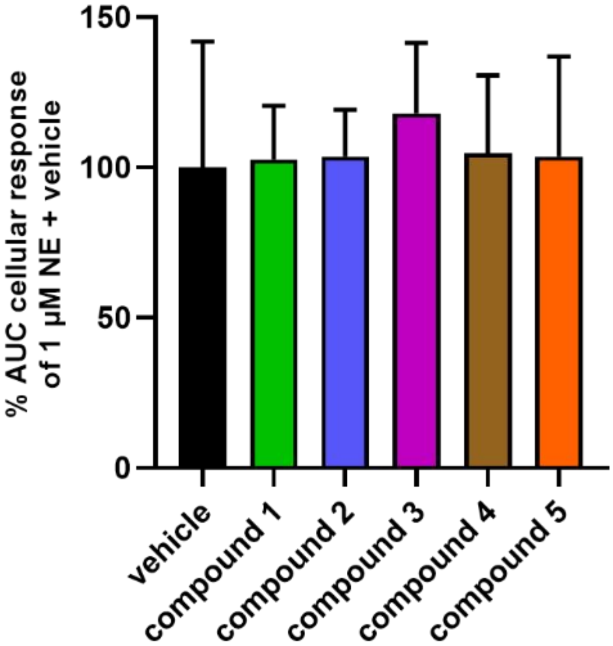

Figure S2. Counter screen of the five hit compounds in a label-free impedance-based TRACT assay. JumpIn-NET were not induced with doxycycline and as such did not express NET. Cells were pretreated for 1 h with either vehicle or 10 µM of the hit compound. Subsequently, cells were stimulated with 1 µM norepinephrine (NE) and Cell Index (CI) was measured for 30 min. Cellular responses are expressed as the net area under the curve (AUC) of the first 30 minutes after stimulation with NE. Data were normalized to the response of NE only (vehicle, 100%). Data are shown as the mean +/- SD of two separate experiments each performed in duplicate.

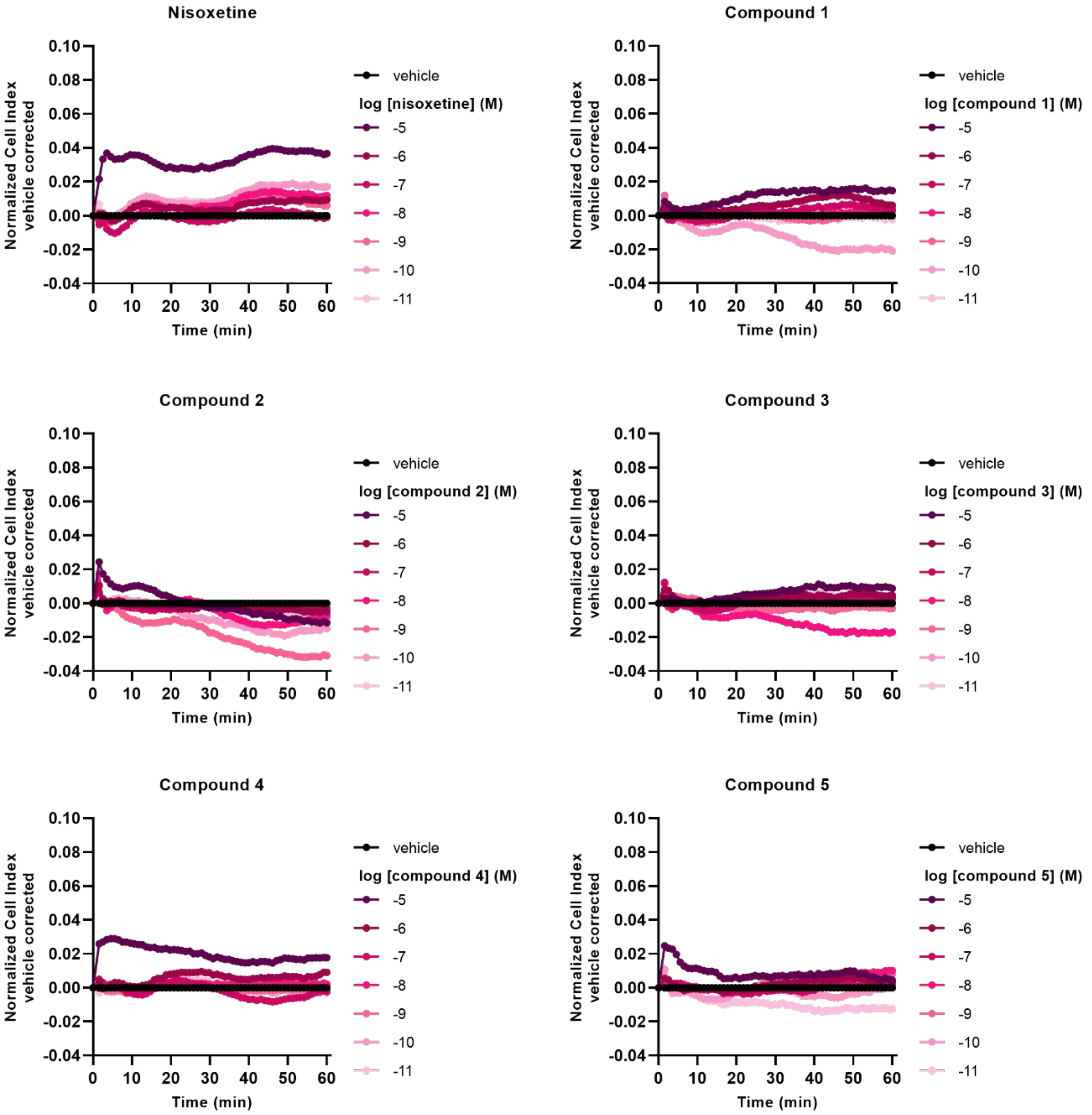

Figure S3. Representative xCELLigence traces of JumpIn-NET cells during inhibitor pretreatment in a label-free impedance-based TRACT assay. Cells were pretreated for 1 h with either vehicle or increasing concentrations of nisoxetine or hit compound. Cell Index was normalized to the time point prior to inhibitor addition (t = 0 min). Data are shown as the mean of a representative experiment.

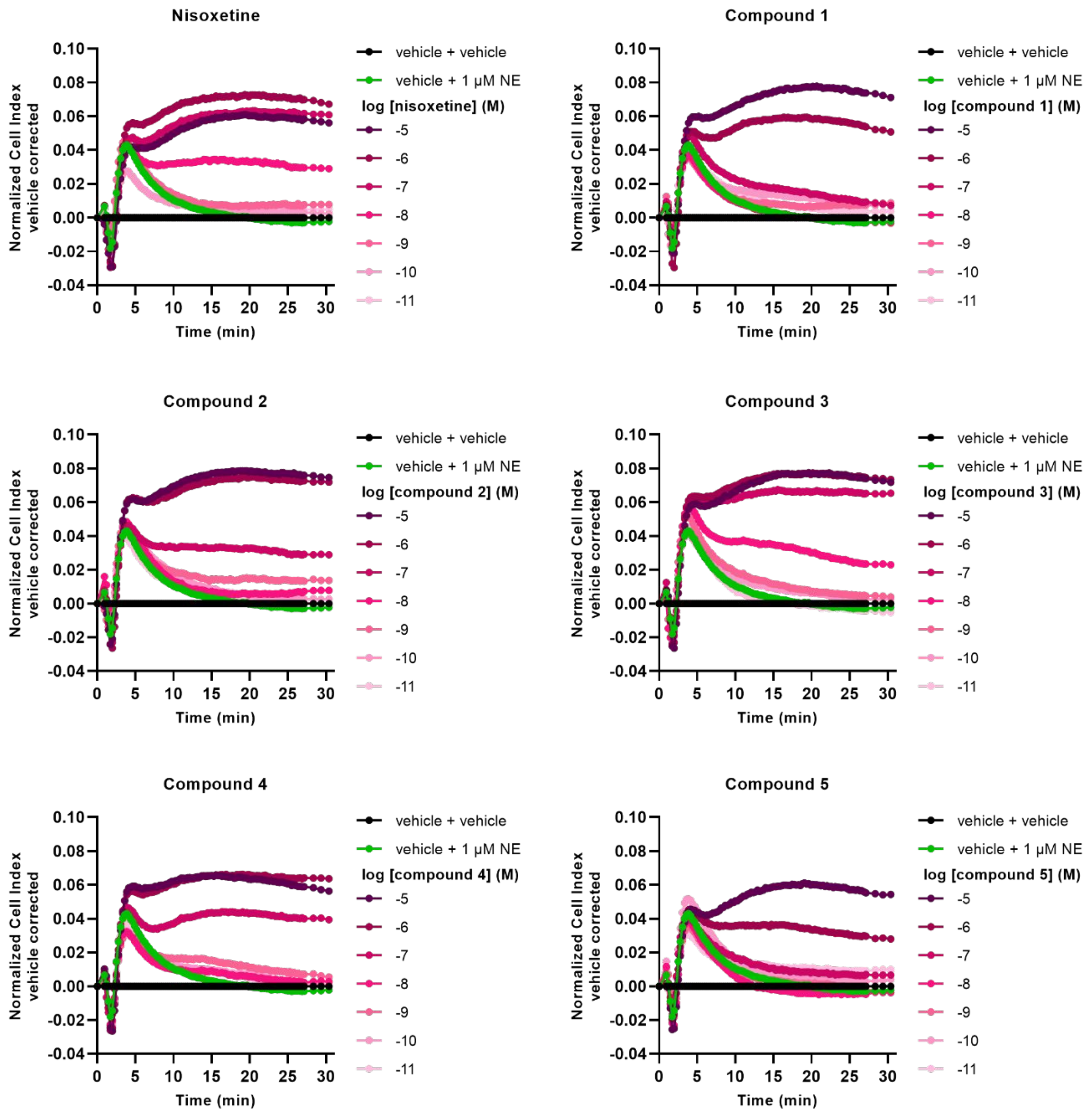

Figure S4. Representative xCELLigence traces of JumpIn-NET cells during norepinephrine (NE) stimulation in a label-free impedance-based TRACT assay. Cells were pretreated for 1 h with either vehicle or increasing concentrations of nisoxetine or hit compound, and subsequently stimulated with vehicle or 1 μM NE. Cell Index was normalized to the time point prior to NE addition (t = 0 min). Data are shown as the mean of a representative experiment.

Table S1. Molecular and Protein descriptors used throughout the model building process.

Molecular Descriptors		Protein Descriptors	
ALogP	SCFC 6	Amino Acid Composition	ACC c scales
Molecular Weight	FPFC 6	Dipeptide Composition	ACC DPPS
Number of Hydrogen Donors	EPFC 6	Auto Correlation Descriptors	ACC E scales
Number of Hydrogen Acceptors	LPFC 6	Composition Transition Distribution	ACC G scales
Number of Rotatable Bonds	SPFC 6	Quasi Sequence Order Descriptors	ACC HESH
Number of Bridge Bonds	FEFC 6	Pseudo Amino Acid Composition	ACC HSEHPCSV
Number of Atoms	EEFC 6	Amphiphilic Pseudo Amino Acid Composition	ACC Norinder
Number of Rings	LEFC 6	Total Amino Acid Properties	ACC Kidera
Number of Aromatic Rings	SEFC 6	Aligned Z scales Sandberg	ACC P scales
Number of Fragments	FHFC 6	Aligned FASGAI	ACC QCP
N Plus O Count	EHFC 6	Aligned BLOSUM	ACC Sneath
Molecular Solubility	LHFC 6	ACC Z scales Hellberg	ACC SVEEVA
Molecular Surface Area	SHFC 6	ACC Z scales Jonsson	ACC SVHEHS
Molecular Polar Surface Area	FCFP 6	ACC Z scales Sandberg	ACC SVRG
Molecular Polar Solvent-Accessible Surface Area (SASA)	ECFP 6	ACC Z scales binary	ACC SVWG
Estate Keys	LCFP 6	ACC T scales	ACC V scales
Estate Counts	SCFP 6	ACC ST scales	ACC VSGETAWAY
MDLPublicKeys	FPFP 6	ACC VHSE	ACC VSTPV
MDL2DKeys960	EPFP 6	ACC ISA ECI	ACC VSW
MDL2DKeys166	LPFP 6	ACC GRID t-score	ACC VTSA
PHFP 2-4	SPFP 6	ACC VSTV	ACC SVGER
PHRFP 2-4	FEFP 6	ACC MSWHIM	ACC PSM
PHPFP 2-4	EEFP 6	ACC_FASGAI	ACC SSIA AM1
PHFC 2-4	LEFP 6	ACC_BLOSUM	ACC SSIA PM3
PHPFC 2-4	SEFP 6	ACC_VARIMAX	ACC SSIA HF
PHRFC 2-4	FHFP 6	ACC Protein fingerprint numerical	ACC SSIA DFT
FCFC 6	EHFP 6	ACC Protein fingerprint hash	
ECFC 6	LHFP 6		
LCFC 6	SHFP 6		

Table S2. Optimal settings obtained from stepwise feature selection and grid parameter optimization. *Displayed are the optimal settings for random forest, gradient boosting, partial least squares, and the final model which contained an ensemble of random Forest and gradient boosting, with a partial least squares stack on top of it.*

Optimal Model	Optimal Features	Optimal Parameters	
**Random Forest**	Num_H_Donors<br>Num_RotatableBonds<br>Num_Atoms<br>Molecular_Solubility<br>Molecular_SurfaceArea<br>PHPFC2<br>PHRFC2<br>SCFC6<br>G9TotalAminoAcidProperties<br>ACC_ISA_ECI<br>ACC_ProtFP_hash	Number of Trees<br>Number of Descriptors<br>Minimum Node Size<br>Maximum Depth	1000<br>Sqrt(D)*<br>1<br>None
**Gradient Boosting**	MDLPublicKeys<br>MDL2DKeys960<br>G4CompositionTransitionDistribution<br>Aligned_FASGAI<br>ACC_VARIMAX<br>ACC_ProtFP_numerical<br>ACC_DPPS<br>ACC_HESH<br>ACC_SVHEHS<br>ACC_VSTPV	Maximum number of Trees<br>Learning Rate<br>Gamma<br>Maximum Depth<br>Data Fraction<br>Descriptor Fraction	1000<br>0.1<br>0.3<br>7<br>1.0<br>0.5
**Partial Least Squares**	MDL2DKeys960<br>LEFC6<br>SCFP6<br>FPFP6<br>FEFP6<br>G1AminoAcidComposition<br>ACC_FASGAI<br>ACC_Norinder<br>ACC_SVRG<br>ACC_SVGER	Number of Variables	200
**1st stack Random Forest & Gradient Boost**<br>**2nd Stack Partial Least Squares**	AlogP<br>Num_H_Acceptors<br>Num_BridgeBonds<br>Molecular_Solubility<br>Estate_Counts<br>PHPFC2<br>G1AminoAcidComposition<br>G9TotalAminoAcidProperties<br>ACC_ISA_ECI<br>Gradient Boost Preditions<br>Random Forest Predictions<br>Random Forest Predictions StdDev	Number of Variables	100

Table S3. Cluster centers that were chosen for experimental follow-up (46 centers). *Of these, 32 were able to be synthesized, as shown in the right-most column.*

Enamine ID Number	SMILES	Synthesized
Z2393213853	CCCN(C(=O)C1=CC=CC=C1OCC1CC1)C1CCNC1	Yes
Z2444727652	CC1=NC=CC=C1CN(CC1=CC=CC=C1)C1CCCNC1	No
Z2769565776	CN(C)C1=CC=C(F)C=C1CNCC1(O)CC2=CC=CC=C2C1	Yes
Z1986623199	CC(N[C@H]1C[C@H](C2=CC=C(Cl)C(Cl)=C2)C1)C1CCOCC1	Yes
Z1904820040	OCCCCNC1CCCC1C1=CC=C(Cl)C(Cl)=C1	Yes
PV-001918160326	CCOC(CN1CC=C(C2=C[NH]C3=CC(F)=CC=C23)CC1)C1CC1	Yes
Z2569757498	FC1=CC=CC([C@@H]2CCNC[C@@H]2NCC2=CC=C3OCCOC3=C2)=C1	Yes
PV-001919825446	OC(CNC(C1=CC=C(F)C(Cl)=C1)C1CC1)COC1=CC=CC2=C1C=C[NH]2	No
Z1083362508	O=C(C1CC1C1=CC=C(Cl)C(Cl)=C1)N1CCCC(OCC(F)(F)F)C1	Yes
Z1783772638	CC(C)(CNCCC1CCOC1)C1=CC=C(Cl)C(Cl)=C1	Yes
Z2444828348	CC1(CN2CCOC(C3=CC=C(Cl)C(Cl)=C3)C2)CCNC1	Yes
Z2698567135	CC(C)(CNCC1CCCOC1)C1=CC=C2C=CC=CC2=C1	Yes
Z1663514926	CC(C)C(NCCC1CCOC1)C1=CC=C(Cl)C(Cl)=C1	Yes
PV-000916662553	C#CCCNCC1CCCN1C(=O)C1=CC=CC2=C1OCC2(C)C	No
PV-001921689112	CCN1CCC(N[C@@H]2CCO[C@H]2C2=CC=C(Cl)C(F)=C2)C1=O	Yes
Z2447436630	ClC1=CC=CC(C2=NC(CN(C3CC3)C3CCNC3)=CO2)=C1	Yes
Z2843312692	CN1C=C(C2CC3CCC(C2)N3CC(O)C2=CC=C(Cl)C(F)=C2)C=N1	Yes
PV-001918950467	FC1=CC=CC(CCN2CCN(C3=C(Cl)C=CC=C3Cl)CC2)=C1	Yes
PV-001919382948	CC1(C2=CC=CC(Cl)=C2)CCN(CC(O)COC2=CC=CC3=C2C=C[NH]3)CC1	No
PV-001919716605	CCC1=CC=C(N2CCN(CC(O)COC3=CC=CC4=C3C=C[NH]4)CC2)C=C1Cl	No
Z2444770829	FC(F)(F)C1=CC(CN(C2CC2)C2CCNC2)=CC=C1Cl	No
Z2445178480	ClC1=CC=C(CCC2=NC(C3CCNCC3)=NO2)C=C1Cl	No
Z2617873335	ClC1=CC=CC(OCC2=NOC(C[C@H]3CCNC3)=N2)=C1Cl	Yes
Z2846041003	CC(C1=CC=C(Cl)C(Cl)=C1)N1C=C(C2CCCNC2)N=N1	Yes
Z2488796202	CCC1(C(=O)N2CC=C(C3=C[NH]C4=CC(F)=CC=C34)CC2)CCCN1	Yes
Z2354724931	COC1=CC=C(CNC2CNCCC2(F)F)C=C1OCC1=CC=CC=C1	No
PV-000923491934	CCCNCC1(C)CCN(C(=O)C2=C[NH]C3=CC=C(F)C=C23)C1	No
PV-001917796783	CCN(CC1(C2=CC=C(Cl)C(Cl)=C2)CCC1)C1CC1	No
Z1421251686	OC(COC1=CC=CC2=C1C=C[NH]2)CN1CCC(C2=CC=C(F)C(F)=C2)CC1	No
Z2436746713	O=C(CCC1CCNC1)N1CCOC(C2=CC=C(Cl)C(Cl)=C2)C1	Yes
PV-001930327687	ClC1=CC=C(C2CCCC2NC[C@@H]2CCCO2)C=C1Cl	No
PV-001923897672	CCC1CC(NCC(C)(C)C2=CC=C(Cl)C(Cl)=C2)CCO1	Yes
Z1505378916	C1=CC=C(C2CN(CCN3C=CN=C3)CC3=CC=CC=C32)C=C1	No
Z2201777668	OCC(NC1CCN(CC2=CC=CC=C2Cl)CC1)C1=CC=C(Br)C(F)=C1	Yes
PV-001918897538	CC1CC(CN(CCO)CC2=CC=C(Cl)C(Cl)=C2)C1	Yes
PV-001917367008	ClC1=CC=C(C2CN(CC[C@@H]3C[C@H]3C3CC3)CCO2)C=C1Cl	Yes
Z2361435743	CC(C)(CNC(=O)NCC1CNCCO1)C1=CC=C(Cl)C(Cl)=C1	Yes
Z1485114833	OC(COC1=CC=CC=C1F)CN1CC=C(C2=C[NH]C3=CC(F)=CC=C23)CC1	Yes
Z3045122589	OC(CNCC1CSC2=CC=CC=C2O1)C1=CC=C(F)C(F)=C1	Yes
PV-001926691632	CC1=CC(Cl)=CC=C1OC1=CC=C(F)C=C1CNC[C@H]1C[C@H](O)C1	Yes

PV-001919611983	CC(C)NC(=O)OCC1CCCCN1C[C@H](O)C1=CC=C(Cl)C(Cl)=C1	Yes
Z2722622816	OC(CNC1CCN(C2=CC=C(Cl)C=C2F)C1)C1=CC=CC(F)=C1	Yes
Z2444696415	CCCN(CC1=CC=C(F)C=C1C(F)(F)F)C1CCNCC1	No
Z3100431738	O=C(C1=CN=C(C2CCNCC2)O1)N(CC1=CC=C(Cl)C(Cl)=C1)C1CC1	Yes
PV-001925822957	CC1=CC(Cl)=CC=C1OC1=CC=C(F)C=C1CNCC(C)CN(C)C	Yes
Z2488563933	CCCN(C(=O)C1=CC=C2[NH]C=CC2=C1)C1CCNC1	Yes

Table S4: Selected cluster compounds and their nearest structurally similar compound in the training set. *Based on Tanimoto Similarity (ECFP_6). The Enamine 'Q-Code' refers to the codes found in Table S6.*

Cluster Molecule		Nearest Training Molecule	
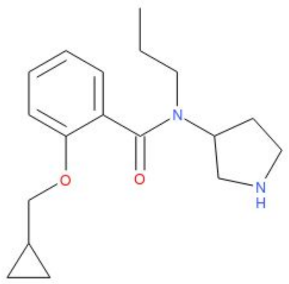	**Cluster SMILES**<br>CCCN(C(=O)C1=CC=CC=C1OCC1CC1)C1CCNC1<br>**Enamine ID**<br>Z2393213853<br>**Enamine Q-Code**<br>Q754936$1	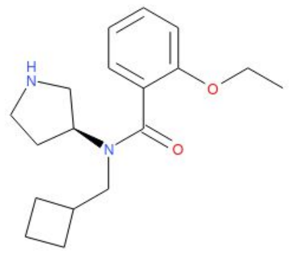	**Training SMILES**<br>CCOc1ccccc1C(=O)N(CC2CCC2)[C@H]3CCNC3<br>**ChEMBL ID**<br>CHEMBL551471<br>**Tanimoto Similarity**<br>0.557
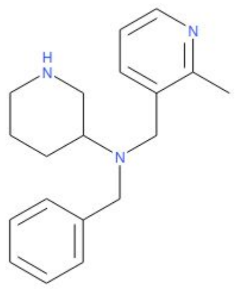	**Cluster SMILES**<br>CC1=NC=CC=C1CN(CC1=CC=CC=C1)C1CCCNC1<br>**Enamine ID**<br>Z2444727652<br>**Enamine Q-Code**<br>n/a	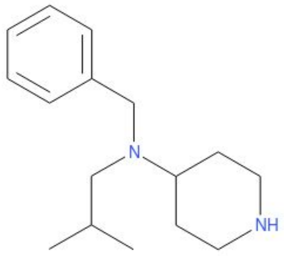	**Training SMILES**<br>CC(C)CN(Cc1ccccc1)C2CCNCC2<br>**ChEMBL ID**<br>CHEMBL207837<br>**Tanimoto Similarity**<br>0.333
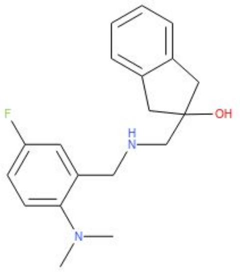	**Cluster SMILES**<br>CN(C)C1=CC=C(F)C=C1CNCC1(O)CC2=CC=CC=C2C1<br>**Enamine ID**<br>Z2769565776<br>**Enamine Q-Code**<br>Q754914$1	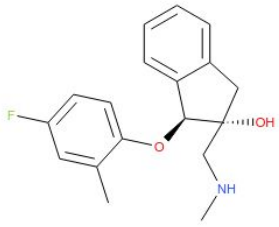	**Training SMILES**<br>CNC[C@]1(O)Cc2ccccc2[C@@H]1Oc3ccc(F)cc3C **ChEMBL ID**<br>CHEMBL506137<br>**Tanimoto Similarity**<br>0.245
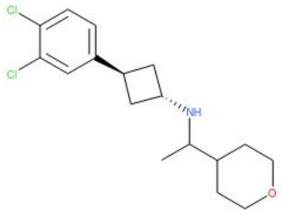	**Cluster SMILES**<br>CC(N[C@H]1C[C@H](C2=CC=C(Cl)C(Cl)=C2)C1)C1CCOCC1<br>**Enamine ID**<br>Z1986623199<br>**Enamine Q-Code**<br>Q754932$4	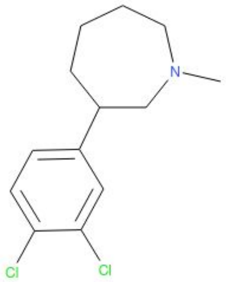	**Training SMILES**<br>CN1CCCCC(C1)c2ccc(Cl)c(Cl)c2<br>**ChEMBL ID**<br>CHEMBL2326691<br>**Tanimoto Similarity**<br>0.311

	Cluster SMILES	Enamine ID	Enamine Q-Code		Training SMILES	ChEMBL ID	Tanimoto Similarity
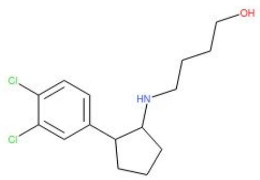	OCCCCNC1CCCC1C1=CC=C(Cl)C(Cl)=C1	Z1904820040	Q754913$15	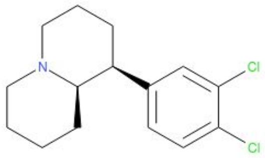	Clc1ccc(cc1Cl)[C@@H]2CCCN3CCCC[C@H]23	CHEMBL559186	0.320
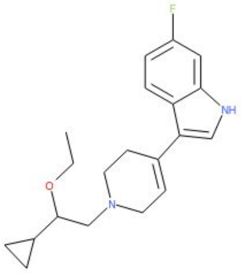	CCOC(CN1CC=C(C2=C[NH]C3=CC(F)=CC=C23)CC1)C1CC1	PV-001918160326	Q754917$41	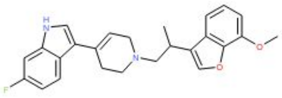	COc1cccc2c(coc12)C(C)CN3CCC(=CC3)c4c[nH]c5cc(F)ccc4 5	CHEMBL597479	0.500
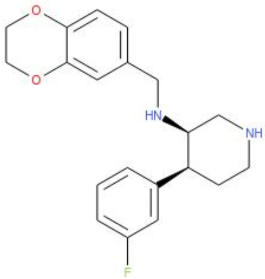	FC1=CC=CC([C@@H]2CCNC[C@@H]2NCC2=CC=C3OCCOC3=C2)=C1	Z2569757498	Q754942$4	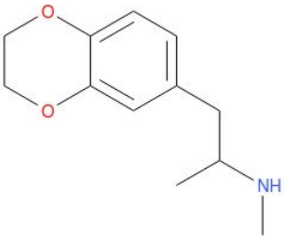	CNC(C)Cc1ccc2OCCOc2c1	CHEMBL3603954	0.287
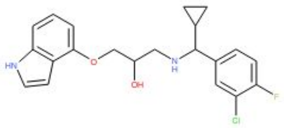	OC(CNC(C1=CC=C(F)C(Cl)=C1)C1CC1)COC1=CC=CC2=C1C=C[NH]2	PV-001919825446	n/a	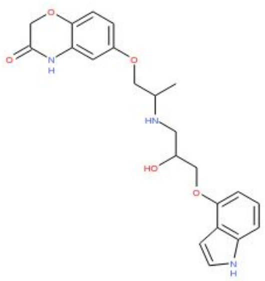	CC(COc1ccc2OCC(=O)Nc2c1)NCC(O)COc3cccc4[nH]ccc34	CHEMBL183921	0.397

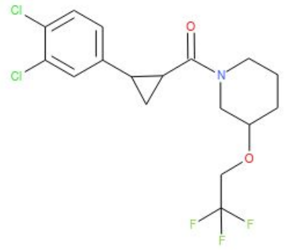

Cluster SMILES

O=C(C1CC1C1=CC=
C(Cl)C(Cl)=C1)N1CC
CC(OCC(F)(F)F)C1

Enamine ID

Z1083362508

Enamine Q-Code

Q754940$2

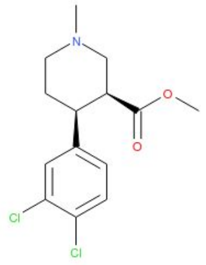

Training SMILES

COC(=O)[C@@H]1
CN(C)CC[C@@H]1c
2ccc(Cl)c(Cl)c2

ChEMBL ID

CHEMBL397490

Tanimoto Similarity

0.333

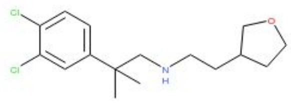

Cluster SMILES

CC(C)(CNCCC1CCO
C1)C1=CC=C(Cl)C(Cl
)=C1

Enamine ID

Z1783772638

Enamine Q-Code

Q754928$34

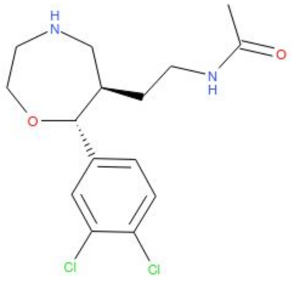

Training SMILES

CC(=O)NCC[C@@H]
1CNCCO[C@H]1c2
ccc(Cl)c(Cl)c2

ChEMBL ID

CHEMBL3593401

Tanimoto Similarity

0.206

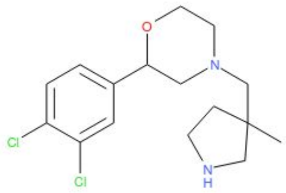

Cluster SMILES

CC1(CN2CCOC(C3=
CC=C(Cl)C(Cl)=C3)C
2)CCNC1

Enamine ID

Z2444828348

Enamine Q-Code

Q754918$1

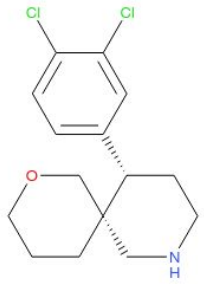

Training SMILES

Clc1ccc(cc1Cl)[C@
@H]2CCNC[C@@]2
3CCCOC3 **ChEMBL ID**

CHEMBL1173607

Tanimoto Similarity

0.286

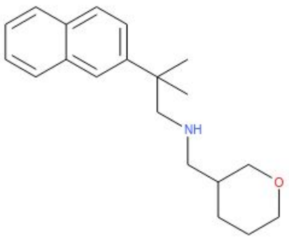

Cluster SMILES

CC(C)(CNCC1CCCO
C1)C1=CC=C2C=CC
=CC2=C1

Enamine ID

Z2698567135

Enamine Q-Code

Q905064$2

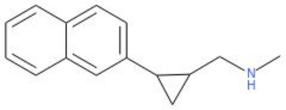

Training SMILES

CNCC1CC1c2ccc3cc
ccc3c2

ChEMBL ID

CHEMBL249331

Tanimoto Similarity

0.253

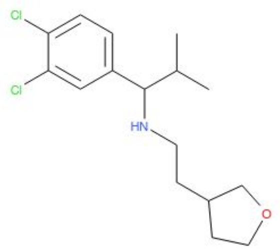

Cluster SMILES

CC(C)C(NCCC1CCOC1)C1=CC=C(Cl)C(Cl)=C1

Enamine ID

Z1663514926

Enamine Q-Code

Q754931$3

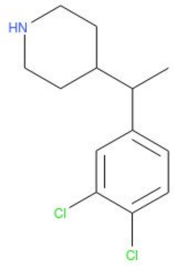

Training SMILES

CC(C1CCNCC1)c2ccc(Cl)c(Cl)c2

ChEMBL ID

CHEMBL2407334

Tanimoto Similarity

0.288

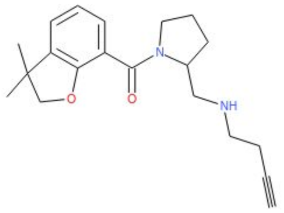

Cluster SMILES

C#CCCNCC1CCCN1C(=O)C1=CC=CC2=C1OCC2(C)C

Enamine ID

PV-000916662553

Enamine Q-Code

n/a

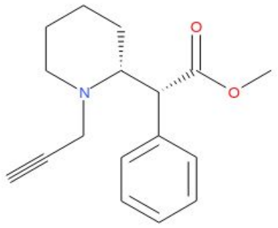

Training SMILES

COC(=O)[C@@H]([C@H]1CCCCN1CC#C)c2ccccc2

ChEMBL ID

CHEMBL1254282

Tanimoto Similarity

0.168

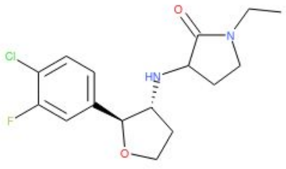

Cluster SMILES

CCN1CCC(N[C@@H]2CCO[C@H]2C2=CC=C(Cl)C(F)=C2)C1=O

Enamine ID

PV-001921689112

Enamine Q-Code

Q754922$1

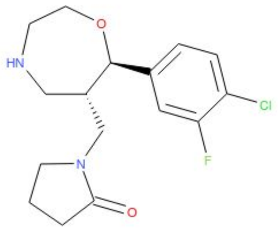

Training SMILES

Fc1cc(ccc1Cl)[C@@H]2OCCNC[C@H]2CN3CCCC3=O

ChEMBL ID

CHEMBL3818953

Tanimoto Similarity

0.347

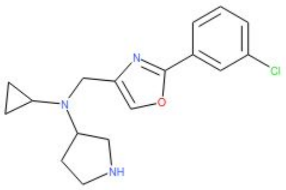

Cluster SMILES

ClC1=CC=CC(C2=NC(CN(C3CC3)C3CCNC3)=CO2)=C1

Enamine ID

Z2447436630

Enamine Q-Code

Q754948$1

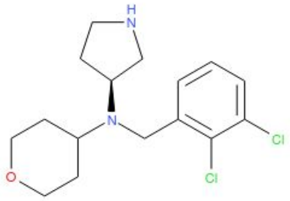

Training SMILES

Clc1cccc(CN(C2CCOCC2)[C@H]3CCNC3)c1Cl

ChEMBL ID

CHEMBL245880

Tanimoto Similarity

0.333

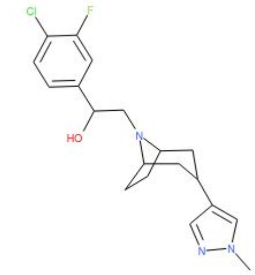	**Cluster SMILES**<br>CN1C=C(C2CC3CCC(C2)N3CC(O)C2=CC=C(Cl)C(F)=C2)C=N1<br>**Enamine ID**<br>Z2843312692<br>**Enamine Q-Code**<br>Q754923$6	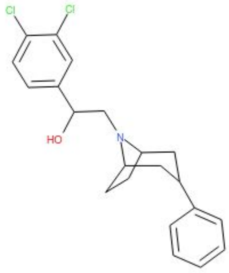	**Training SMILES**<br>OC(CN1C2CCC1CC(C2)c3ccccc3)c4ccc(Cl)c(Cl)c4 **ChEMBL ID**<br>CHEMBL127013<br>**Tanimoto Similarity**<br>0.476
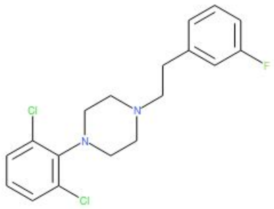	**Cluster SMILES**<br>FC1=CC=CC(CCN2CCN(C3=C(Cl)C=CC=C3Cl)CC2)=C1<br>**Enamine ID**<br>PV-001918950467<br>**Enamine Q-Code**<br>Q754934$8	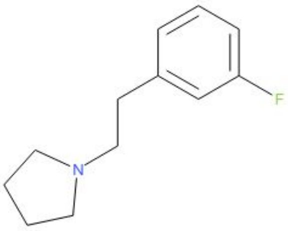	**Training SMILES**<br>Fc1cccc(CCN2CCCC2)c1<br>**ChEMBL ID**<br>CHEMBL2030636<br>**Tanimoto Similarity**<br>0.561
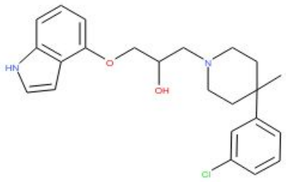	**Cluster SMILES**<br>CC1(C2=CC=CC(Cl)=C2)CCN(CC(O)COC2=CC=CC3=C2C=C[NH]3)CC1<br>**Enamine ID**<br>PV-001919382948<br>**Enamine Q-Code**<br>n/a	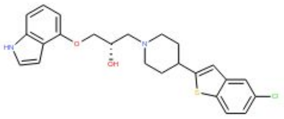	**Training SMILES**<br>O[C@H](COc1cccc2[nH]ccc12)CN3CCC(CC3)c4cc5cc(Cl)ccc5s4<br>**ChEMBL ID**<br>CHEMBL56303<br>**Tanimoto Similarity**<br>0.505
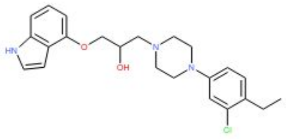	**Cluster SMILES**<br>CCC1=CC=C(N2CCN(CC(O)COC3=CC=CC4=C3C=C[NH]4)CC2)C=C1Cl<br>**Enamine ID**<br>PV-001919716605<br>**Enamine Q-Code**<br>n/a	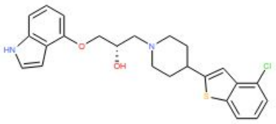	**Training SMILES**<br>O[C@H](COc1cccc2[nH]ccc12)CN3CCC(CC3)c4cc5c(Cl)cccc5s4<br>**ChEMBL ID**<br>CHEMBL57296<br>**Tanimoto Similarity**<br>0.495

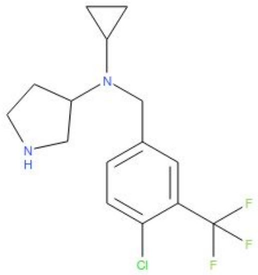

Cluster SMILES

FC(F)(F)C1=CC(CN(C2CC2)C2CCNC2)=CC=C1Cl

Enamine ID

Z2444770829

Enamine Q-Code

n/a

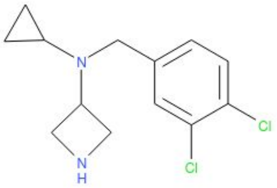

Training SMILES

Clc1ccc(CN(C2CC2)C3CNC3)cc1Cl

ChEMBL ID

CHEMBL3331484

Tanimoto Similarity

0.469

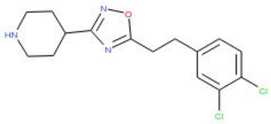

Cluster SMILES

ClC1=CC=C(CCC2=NC(C3CCNCC3)=NO2)C=C1Cl

Enamine ID

Z2445178480

Enamine Q-Code

n/a

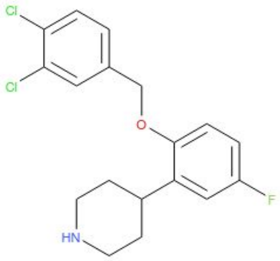

Training SMILES

Fc1ccc(OCc2ccc(Cl)c(Cl)c2)c(c1)C3CCNCC3

ChEMBL ID

CHEMBL573971

Tanimoto Similarity

0.318

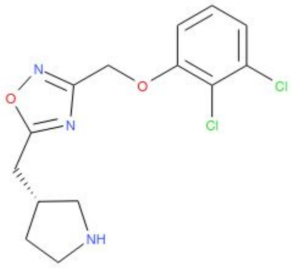

Cluster SMILES

ClC1=CC=CC(OCC2=NOC(C[C@H]3CCNC3)=N2)=C1Cl

Enamine ID

Z2617873335

Enamine Q-Code

Q805275$4

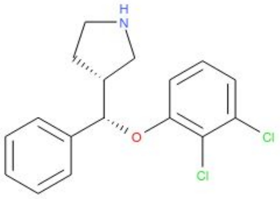

Training SMILES

Clc1cccc(O[C@@H]([C@@H]2CCNC2)c3ccccc3)c1Cl

ChEMBL ID

CHEMBL2337603

Tanimoto Similarity

0.293

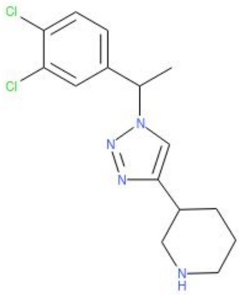

Cluster SMILES

CC(C1=CC=C(Cl)C(Cl)=C1)N1C=C(C2CCCNC2)N=N1

Enamine ID

Z2846041003

Enamine Q-Code

Q754915$3

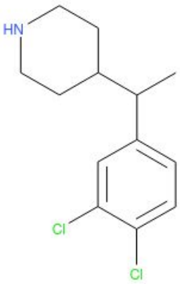

Training SMILES

CC(C1CCNCC1)c2ccc(Cl)c(Cl)c2

ChEMBL ID

CHEMBL2407334

Tanimoto Similarity

0.312

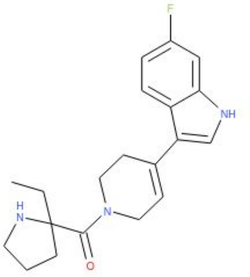	**Cluster SMILES**<br>CCC1(C(=O)N2CC=C(C3=C[NH]C4=CC(F)=CC=C34)CC2)CCCN1<br>**Enamine ID**<br>Z2488796202<br>**Enamine Q-Code**<br>Q754937$42	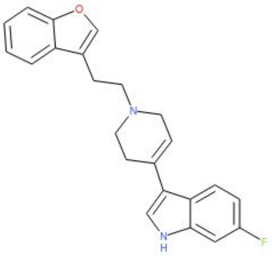	**Training SMILES**<br>Fc1ccc2c(c[nH]c2c1)C3=CCN(CCc4coc5ccccc45)CC3<br>**ChEMBL ID**<br>CHEMBL599985<br>**Tanimoto Similarity**<br>0.387
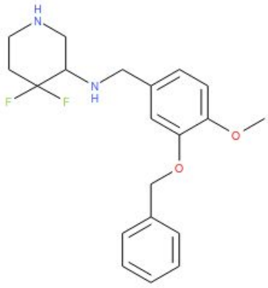	**Cluster SMILES**<br>CCCN(C(=O)C1=CC=CC=C1OCC1CC1)C1CCNC1<br>**Enamine ID**<br>Z2354724931<br>**Enamine Q-Code**<br>n/a	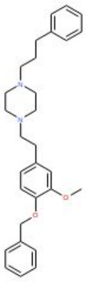	**Training SMILES**<br>COc1cc(CCN2CCN(CCCc3ccccc3)CC2)ccc1OCc4ccccc4<br>**ChEMBL ID**<br>CHEMBL551471<br>**Tanimoto Similarity**<br>0.557
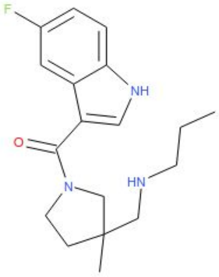	**Cluster SMILES**<br>CCCNCC1(C)CCN(C(=O)C2=C[NH]C3=CC=C(F)C=C23)C1<br>**Enamine ID**<br>PV-000923491934<br>**Enamine Q-Code**<br>n/a	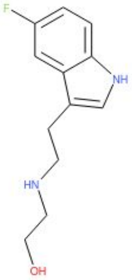	**Training SMILES**<br>OCCNCCc1c[nH]c2ccc(F)cc12<br>**ChEMBL ID**<br>CHEMBL2347166<br>**Tanimoto Similarity**<br>0.266
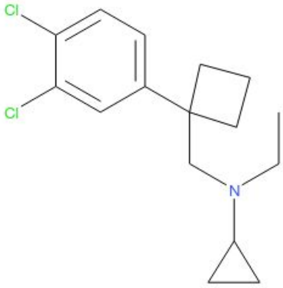	**Cluster SMILES**<br>CCN(CC1(C2=CC=C(Cl)C(Cl)=C2)CCC1)C1CC1<br>**Enamine ID**<br>PV-001917796783<br>**Enamine Q-Code**<br>n/a	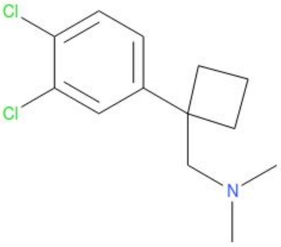	**Training SMILES**<br>CN(C)CC1(CCC1)c2ccc(Cl)c(Cl)c2<br>**ChEMBL ID**<br>CHEMBL1683883<br>**Tanimoto Similarity**<br>0.596

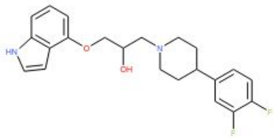	**Cluster SMILES**<br>OC(COC1=CC=CC2=C1C=C[NH]2)CN1CCC(C2=CC=C(F)C(F)=C2)CC1<br>**Enamine ID**<br>Z1421251686<br>**Enamine Q-Code**<br>n/a	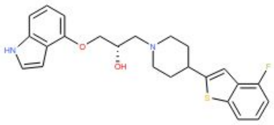	**Training SMILES**<br>O[C@H](COc1cccc2[nH]ccc12)CN3CCC(CC3)c4cc5c(F)cccc5s4<br>**ChEMBL ID**<br>CHEMBL301677<br>**Tanimoto Similarity**<br>0.616
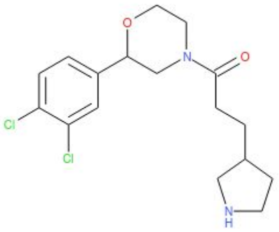	**Cluster SMILES**<br>O=C(CCC1CCNC1)N1CCOC(C2=CC=C(Cl)C(Cl)=C2)C1<br>**Enamine ID**<br>Z2436746713<br>**Enamine Q-Code**<br>Q754947$2	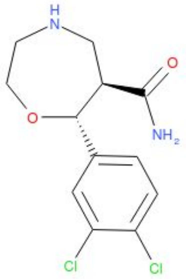	**Training SMILES**<br>NC(=O)[C@@H]1CNCCO[C@H]1c2ccc(Cl)c(Cl)c2 **ChEMBL ID**<br>CHEMBL3593397<br>**Tanimoto Similarity**<br>0.308
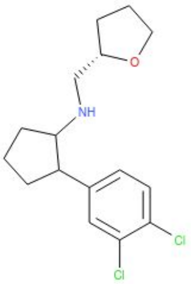	**Cluster SMILES**<br>ClC1=CC=C(C2CCCC2NC[C@@H]2CCCO2)C=C1Cl<br>**Enamine ID**<br>PV-001930327687<br>**Enamine Q-Code**<br>n/a	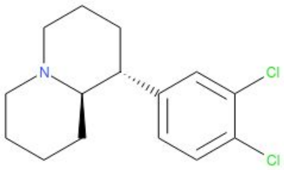	**Training SMILES**<br>Clc1ccc(cc1Cl)[C@H]2CCCN3CCCC[C@H]23<br>**ChEMBL ID**<br>CHEMBL556365<br>**Tanimoto Similarity**<br>0.308
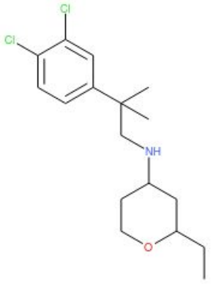	**Cluster SMILES**<br>CCC1CC(NCC(C)(C)C2=CC=C(Cl)C(Cl)=C2)CCO1<br>**Enamine ID**<br>PV-001923897672<br>**Enamine Q-Code**<br>Q754950$3	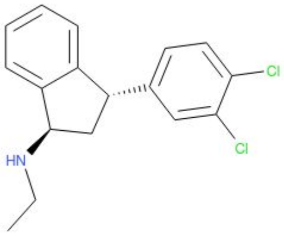	**Training SMILES**<br>CCN[C@@H]1C[C@@H](c2ccc(Cl)c(Cl)c2)c3ccccc13<br>**ChEMBL ID**<br>CHEMBL147983<br>**Tanimoto Similarity**<br>0.216

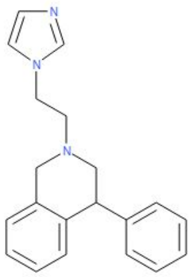

Cluster SMILES

C1=CC=C(C2CN(CC
N3C=CN=C3)CC3=C
C=CC=C32)C=C1

Enamine ID

Z1505378916

Enamine Q-Code

n/a

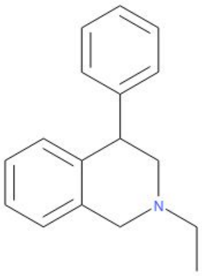

Training SMILES

CCN1CC(c2ccccc2)c
3ccccc3C1

ChEMBL ID

CHEMBL286455

Tanimoto Similarity

0.529

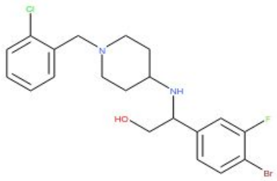

Cluster SMILES

OCC(NC1CCN(CC2=
CC=CC=C2Cl)CC1)C
1=CC=C(Br)C(F)=C1

Enamine ID

Z2201777668

Enamine Q-Code

Q754941$1

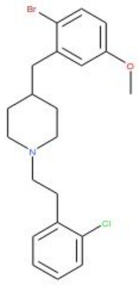

Training SMILES

COc1ccc(Br)c(CC2C
CN(CCc3ccccc3Cl)C
C2)c1

ChEMBL ID

CHEMBL3913057

Tanimoto Similarity

0.267

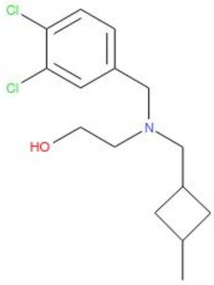

Cluster SMILES

CC1CC(CN(CCO)CC2
=CC=C(Cl)C(Cl)=C2)
C1

Enamine ID

PV-001918897538

Enamine Q-Code

Q754938$4

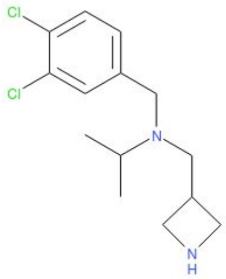

Training SMILES

CC(C)N(CC1CNC1)C
c2ccc(Cl)c(Cl)c2

ChEMBL ID

CHEMBL3331503

Tanimoto Similarity

0.557

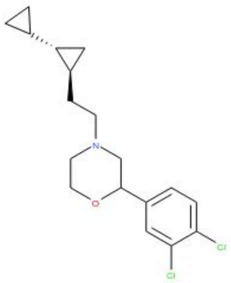

Cluster SMILES

ClC1=CC=C(C2CN(C
C[C@@H]3C[C@H]
3C3CC3)CCO2)C=C1
Cl

Enamine ID

PV-001917367008

Enamine Q-Code

Q754916$4

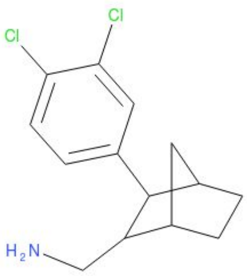

Training SMILES

NCC1C2CCC(C2)C1c
3ccc(Cl)c(Cl)c3

ChEMBL ID

CHEMBL172523

Tanimoto Similarity

0.262

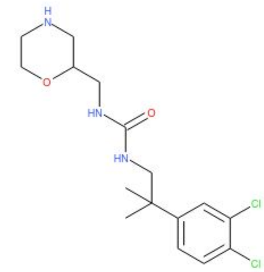

Cluster SMILES

CC(C)(CNC(=O)NCC1CNCCO1)C1=CC=C(Cl)C(Cl)=C1

Enamine ID

Z2361435743

Enamine Q-Code

Q754949$3

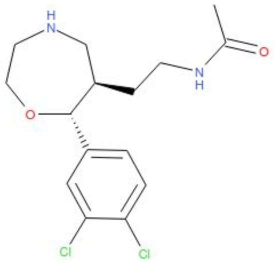

Training SMILES

CC(=O)NCC[C@@H]1CNCCO[C@H]1c2ccc(Cl)c(Cl)c2

ChEMBL ID

CHEMBL3593401

Tanimoto Similarity

0.326

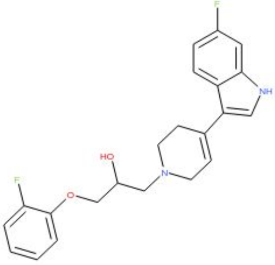

Cluster SMILES

OC(COC1=CC=CC=C1F)CN1CC=C(C2=C[NH]C3=CC(F)=CC=C23)CC1

Enamine ID

Z1485114833

Enamine Q-Code

Q754935$2

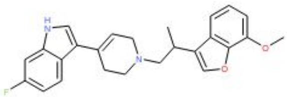

Training SMILES

COc1cccc2c(coc12)C(C)CN3CCC(=CC3)c4c[nH]c5cc(F)ccc4 5

ChEMBL ID

CHEMBL597479

Tanimoto Similarity

0.491

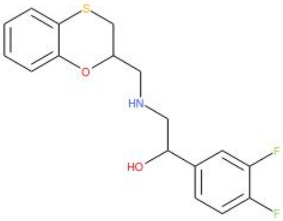

Cluster SMILES

OC(CNCC1CSC2=CC=CC=C2O1)C1=CC=C(F)C(F)=C1

Enamine ID

Z3045122589

Enamine Q-Code

Q754924$1

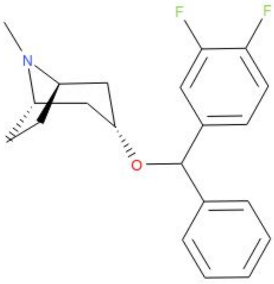

Training SMILES

CN1[C@@H]2CC[C@H]1C[C@H](C2)OC(c3ccccc3)c4ccc(F)c(F)c4

ChEMBL ID

CHEMBL3084908

Tanimoto Similarity

0.196

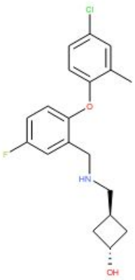

Cluster SMILES

CC1=CC(Cl)=CC=C1OC1=CC=C(F)C=C1CNC[C@H]1C[C@H](O)C1

Enamine ID

PV-001926691632

Enamine Q-Code

Q753945$26

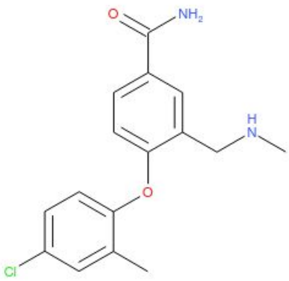

Training SMILES

CNCc1cc(ccc1Oc2ccc(Cl)cc2C)C(=O)N

ChEMBL ID

CHEMBL401819

Tanimoto Similarity

0.388

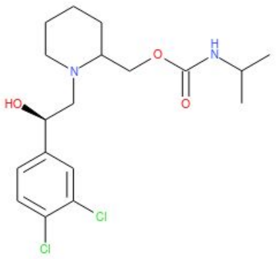

Cluster SMILES

CC(C)NC(=O)OCC1CCCN1C[C@H](O)C1=CC=C(Cl)C(Cl)=C1

Enamine ID

PV-001919611983

Enamine Q-Code

Q754930$6

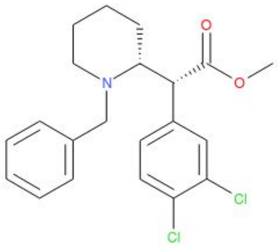

Training SMILES

COC(=O)[C@@H]([C@H]1CCCCN1Cc2ccccc2)c3ccc(Cl)c(Cl)c3

ChEMBL ID

CHEMBL1254674

Tanimoto Similarity

0.314

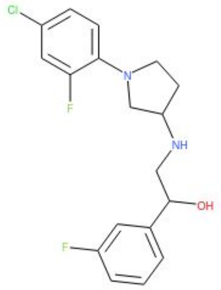

Cluster SMILES

OC(CNC1CCN(C2=CC=C(Cl)C=C2F)C1)C1=CC=CC(F)=C1

Enamine ID

Z2722622816

Enamine Q-Code

Q754933$1

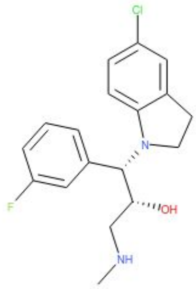

Training SMILES

CNC[C@@H](O)[C@@H](N1CCc2cc(Cl)ccc12)c3cccc(F)c3

ChEMBL ID

CHEMBL598168

Tanimoto Similarity

0.327

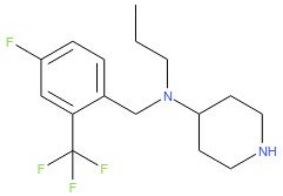

Cluster SMILES

CCCN(CC1=CC=C(F)C=C1C(F)(F)F)C1CCNCC1

Enamine ID

Z2444696415

Enamine Q-Code

n/a

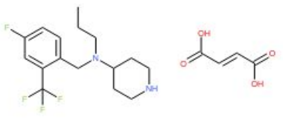

Training SMILES

CCCN(Cc1ccc(F)cc1C(F)(F)F)C2CCNCC2.OC(=O)\C=C\C(=O) O

ChEMBL ID

CHEMBL207854

Tanimoto Similarity

0.833

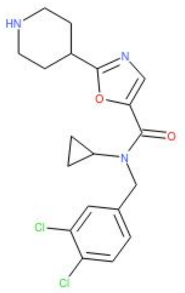

Cluster SMILES

O=C(C1=CN=C(C2CCNCC2)O1)N(CC1=CC=C(Cl)C(Cl)=C1)C1CC1

Enamine ID

Z3100431738

Enamine Q-Code

Q754929$5

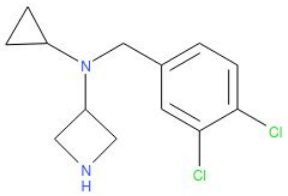

Training SMILES

Clc1ccc(CN(C2CC2)C3CNC3)cc1Cl

ChEMBL ID

CHEMBL3331484

Tanimoto Similarity

0.341

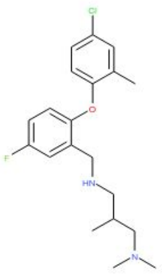

Cluster SMILES

CC1=CC(Cl)=CC=C1
OC1=CC=C(F)C=C1C
NCC(C)CN(C)C

Enamine ID

PV-001925822957

Enamine Q-Code

Q754951$48

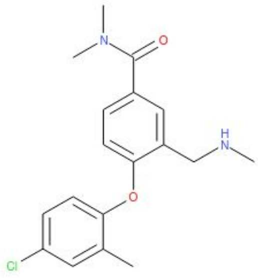

Training SMILES

CNCc1cc(ccc1Oc2cc
c(Cl)cc2C)C(=O)N(C)C

ChEMBL ID

CHEMBL272493

Tanimoto Similarity

0.407

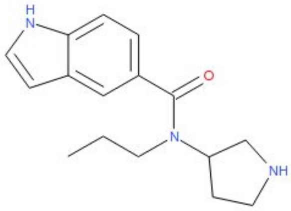

Cluster SMILES

CCCN(C(=O)C1=CC=
C2[NH]C=CC2=C1)C
1CCNC1

Enamine ID

Z2488563933

Enamine Q-Code

Q754912$22

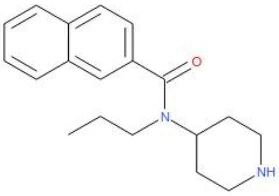

Training SMILES

CCCN(C1CCNCC1)C(=
O)c2ccc3ccccc3c2

ChEMBL ID

CHEMBL565638

Tanimoto Similarity

0.434

Table S5. Single Point screen compounds and their inhibitory activity at 10 µM. *Nisoxertine was set as the baseline 100% (pIC$_{50}$ of 8.0). These values correspond to Figure 8 in the main Manuscript. Data are reported as the mean (in %) ± SEM of three individual experiments each performed in duplicate. Structures shown here might slightly deviate in orientation compared to the main manuscript.*

Enamine ID	ID	Structure (based on SMILES)	Mean (%)	± SEM
**Nisoxetine**		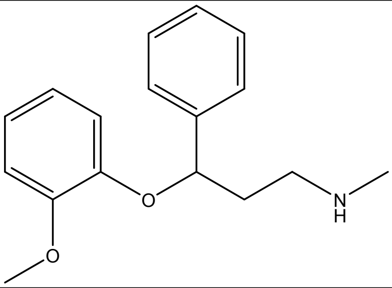	100	12
PV-001921689112	01	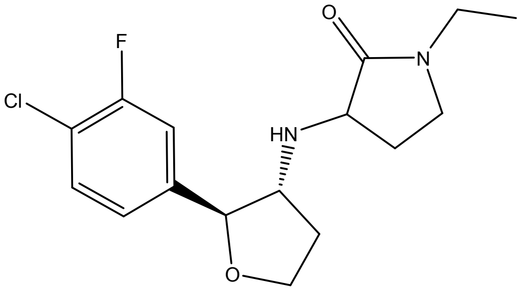	8	9
PV-001919611983	02	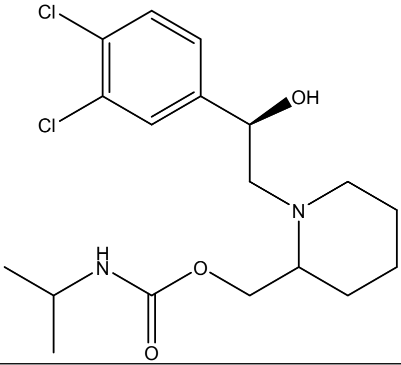	14	11
PV-001917367008	03	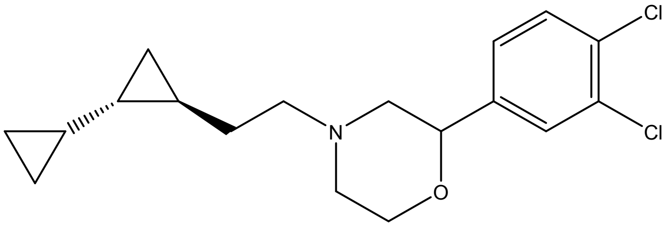	7	9
Z1083362508	04	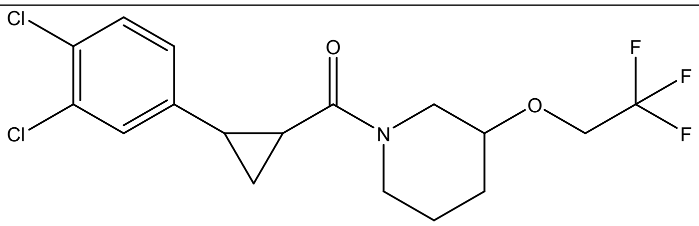	19	15

Z1485114833	05	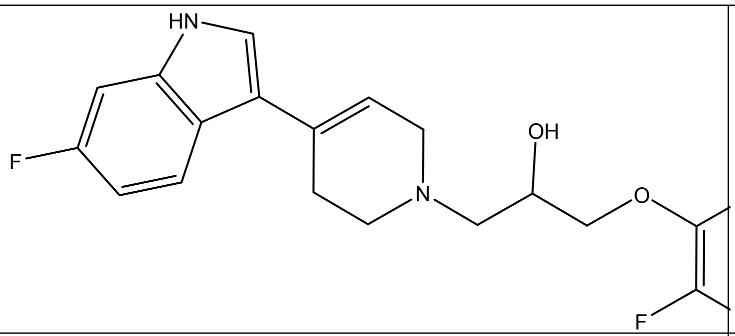	21	4
PV-001918897538	06	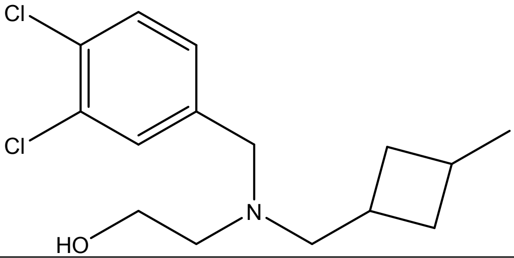	32	3
Z2843312692	07	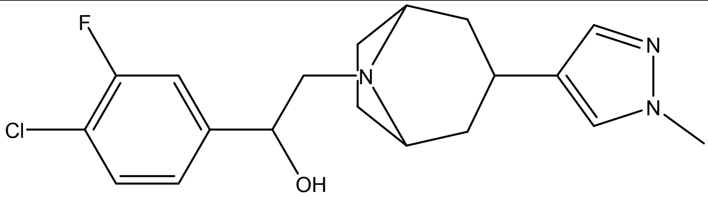	53	7
Z2444828348	08	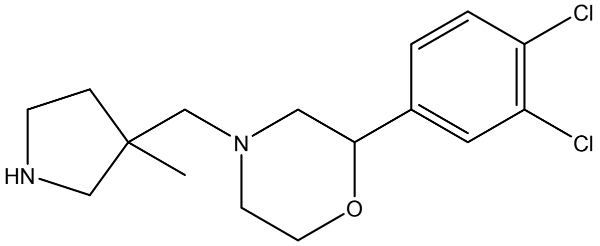	58	4
Z2722622816	09	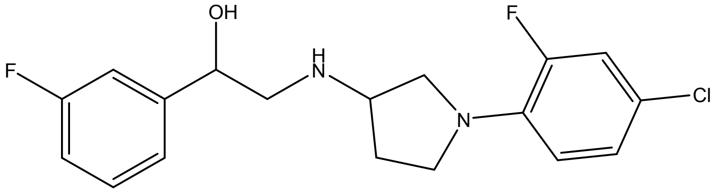	10	7
Z2769565776	10	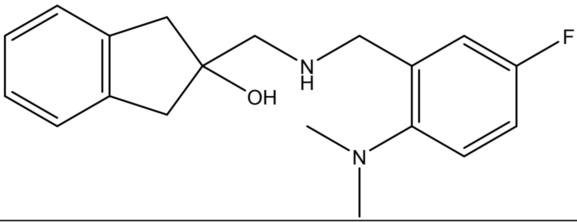	-4	3
Z1986623199	11	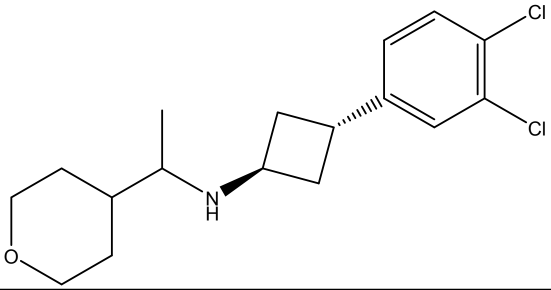	15	5

Z3045122589	12	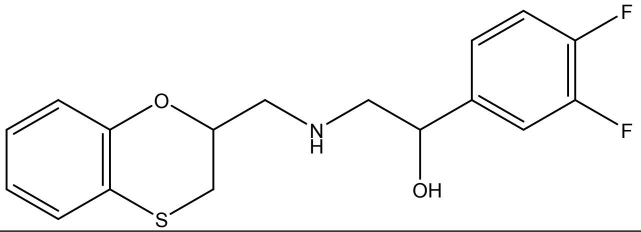	10	10
Z2393213853	13	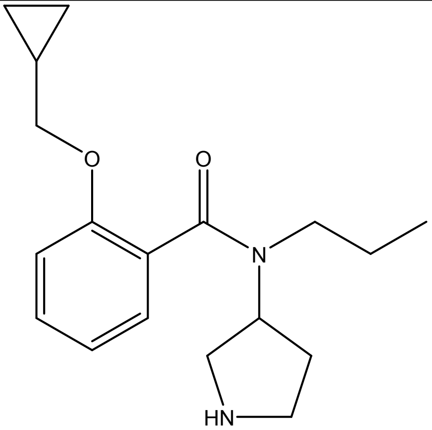	117	10
Z2569757498	14	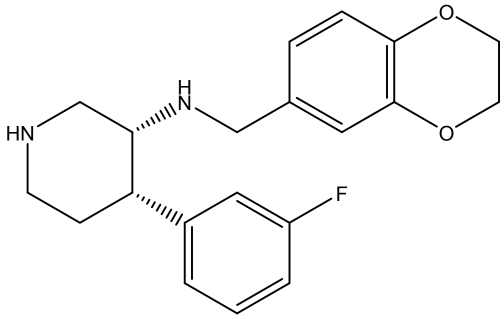	48	9
Z2846041003	15	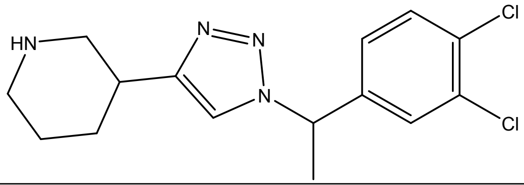	56	8
Z3100431738	16	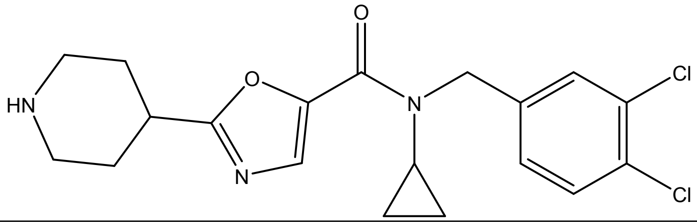	108	12
Z2436746713	17	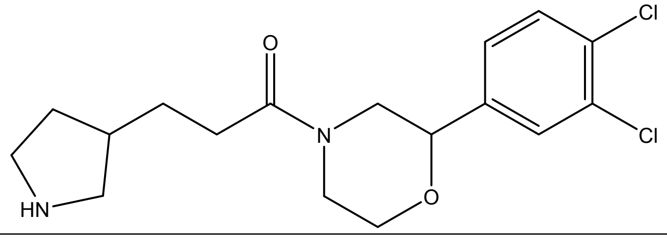	35	9

Z1663514926	18	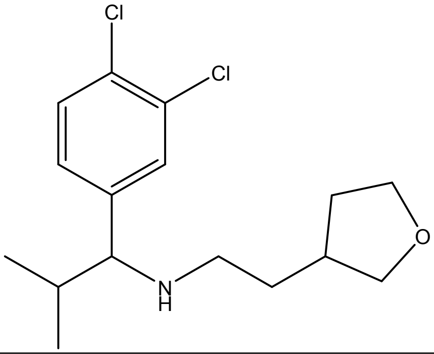	62	7
Z2201777668	19	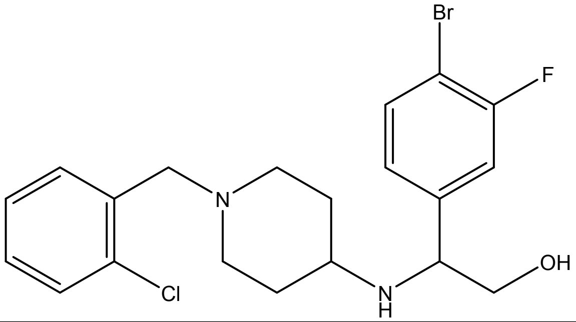	25	13
Z2447436630	20	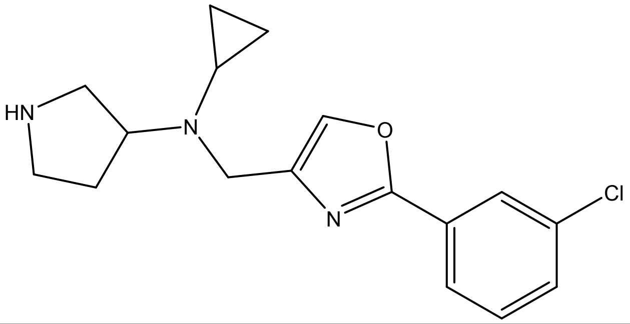	23	7
PV-001918950467	21	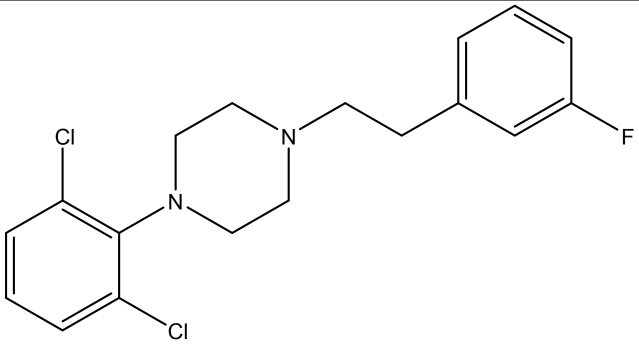	21	7
Z2488563933	22	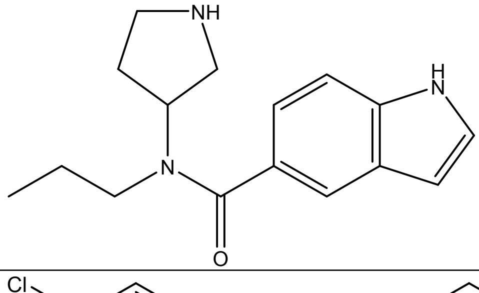	117	5
PV-001923897672	23	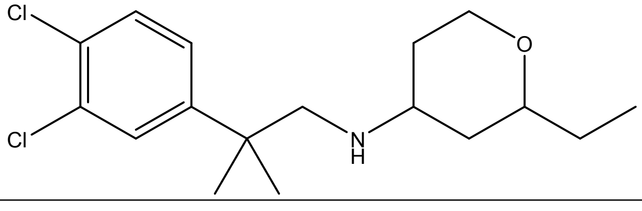	38	2

Z1904820040	24	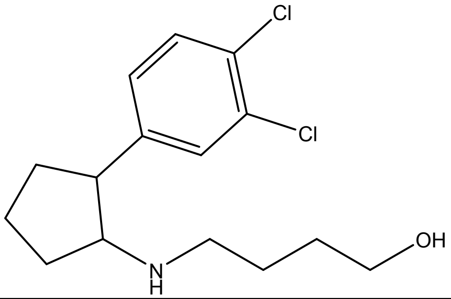	100	11
PV-001926691632	25	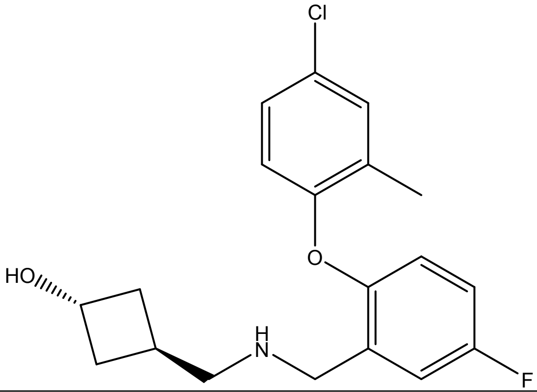	19	6
Z2488796202	26	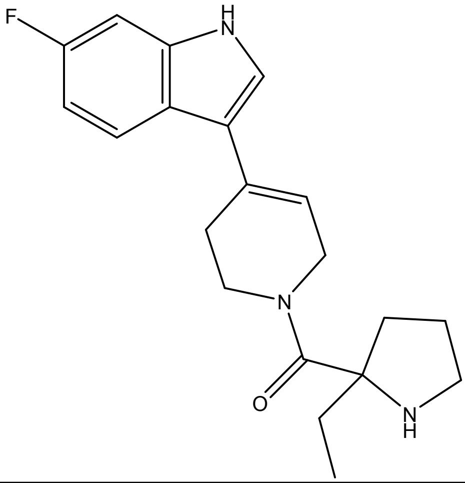	11	10
Z1783772638	27	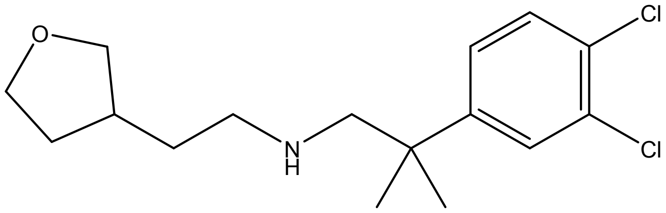	52	8
PV-001925822957	28	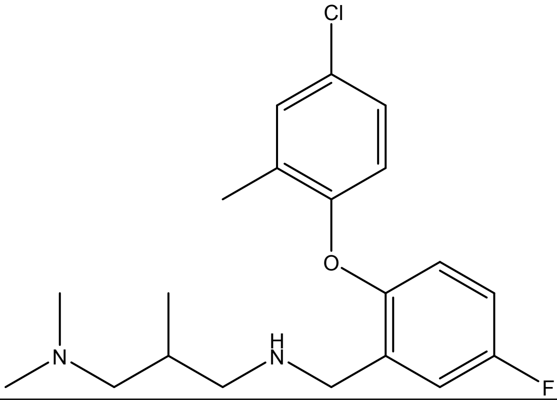	32	12

Z2617873335	29	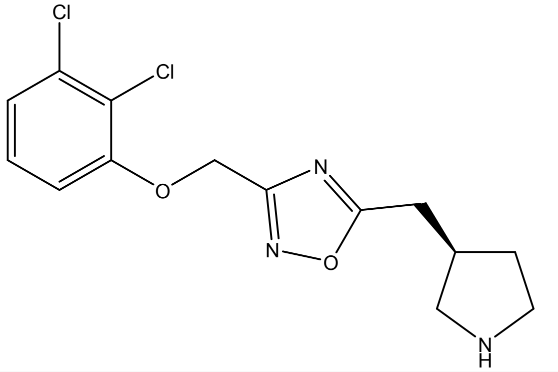	5	6
PV-001918160326	30	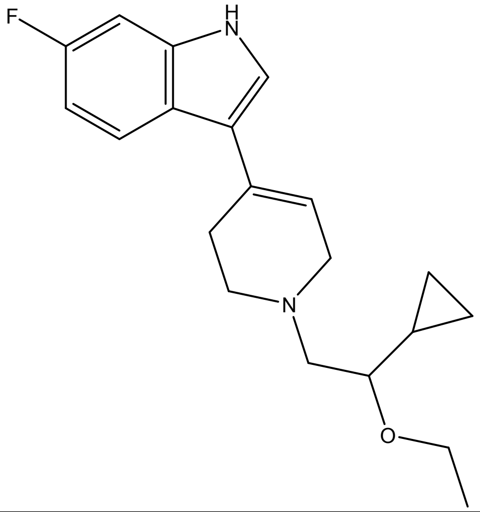	-10	7
Z2361435743	31	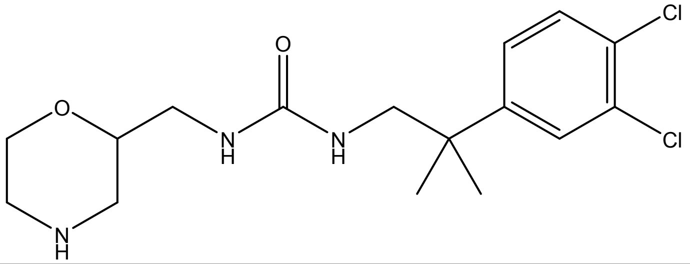	75	5
Z2698567135	32	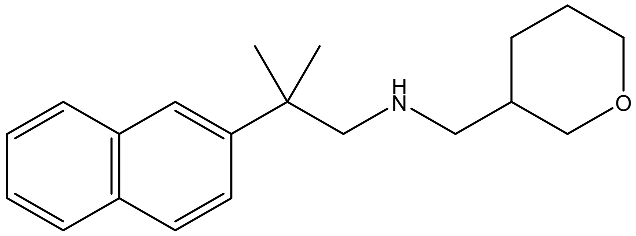	90	11

***S6.* Analytical spectra for the 32 synthesized compounds selected for experimental follow-up.** Data provided by Enamine.

MaxPeak: 100.00%
Ret_Time: 1.248 min

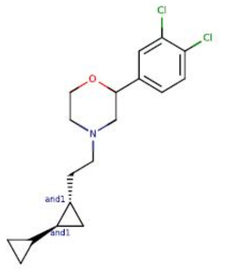

Mol Wt 340.29
Exact Mass 339.16

#	Time	Area%
1	1.248	100.00

RT 1.276

RT 1.282

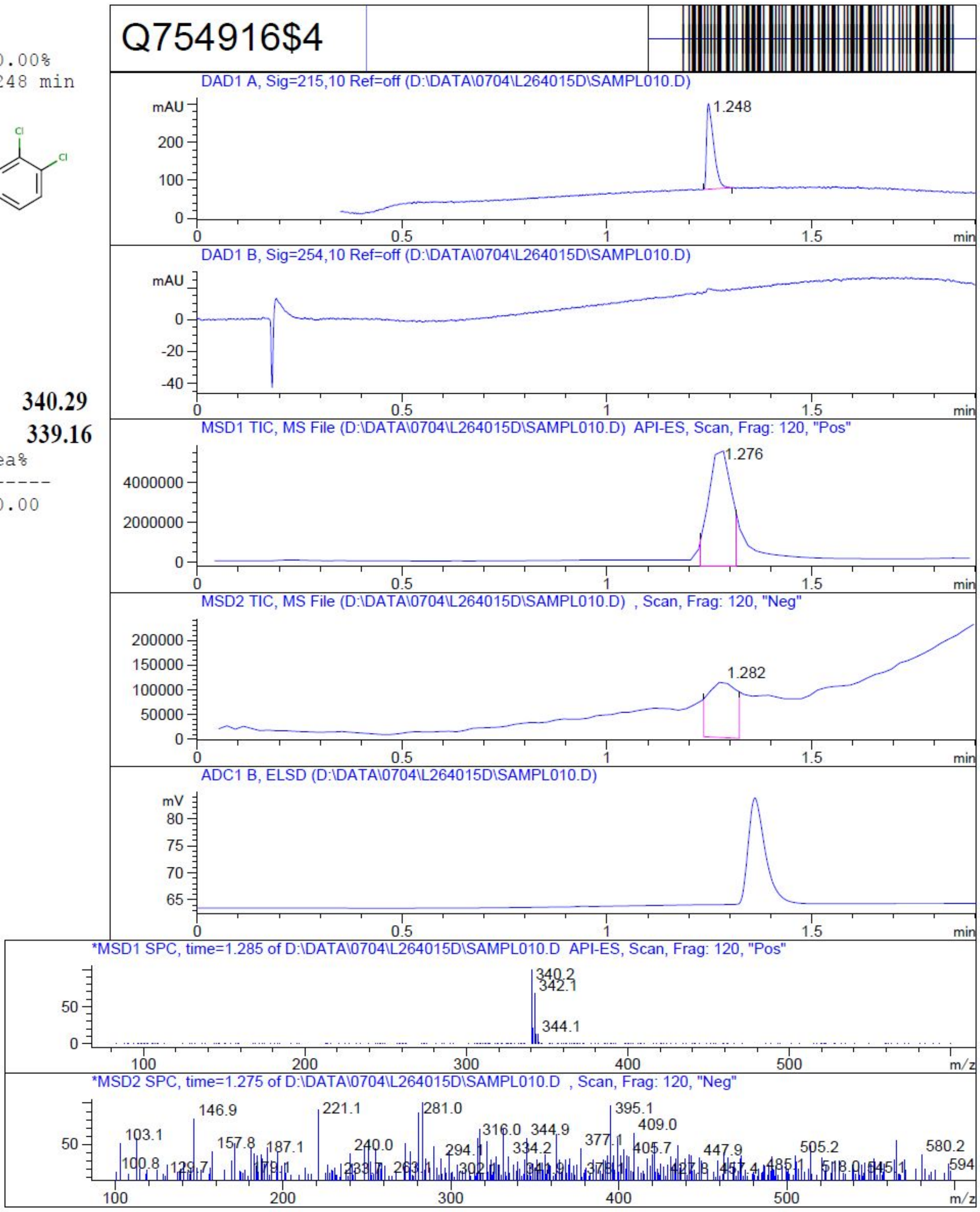

Enamine ID: PV-001917367008

MaxPeak: 96.65%
Ret_Time: 0.956 min

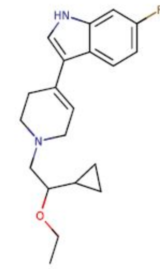

Mol Wt 328.42
Exact Mass 328.24

#	Time	Area%
1	0.956	96.65
2	0.986	3.35

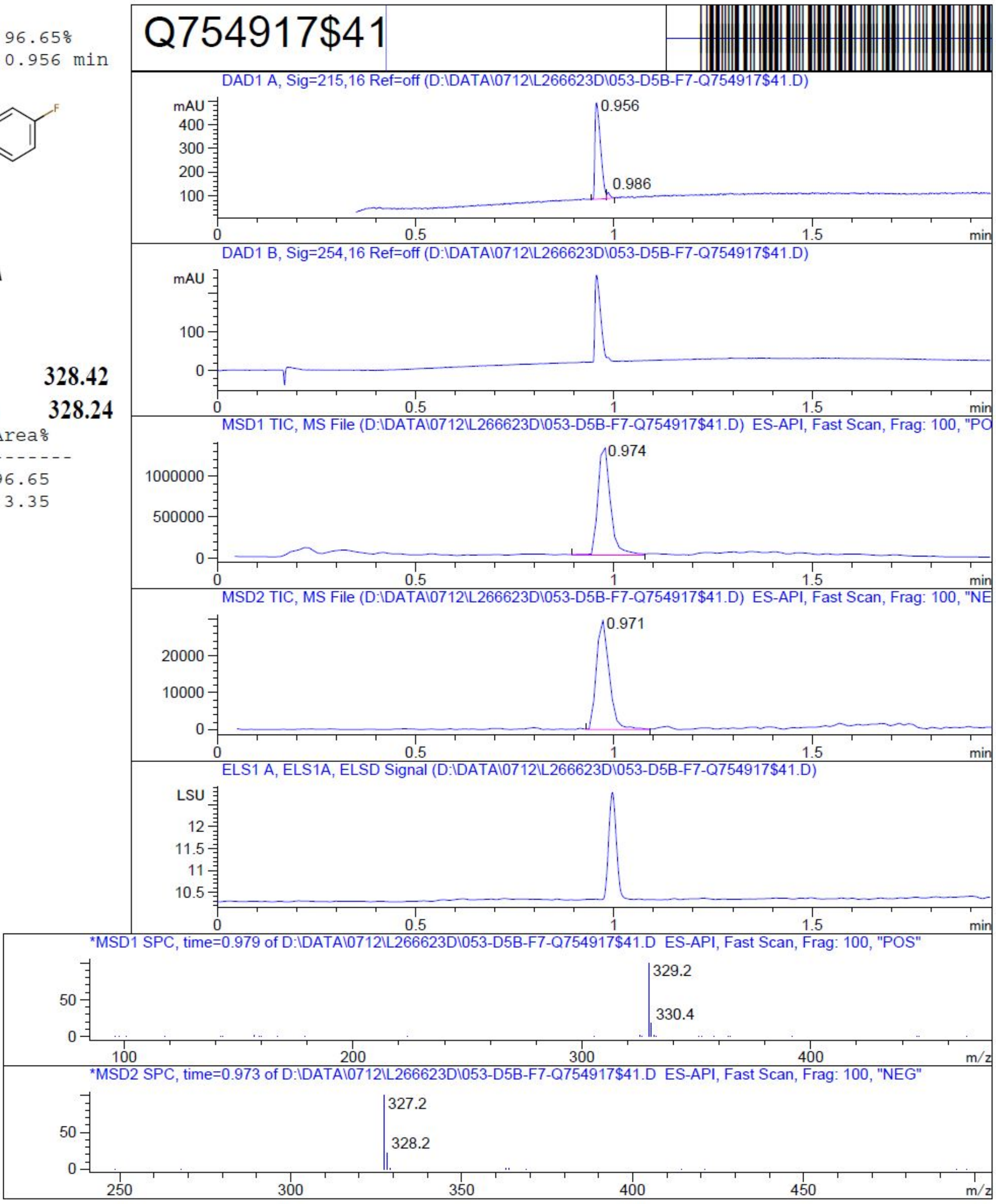

RT 0.974

RT 0.971

Enamine ID: PV-001918160326

MaxPeak: 100.00%
Ret_Time: 1.156 min

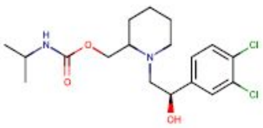

Mol Wt 389.32
Exact Mass 388.17

#	Time	Area%
1	1.156	100.00

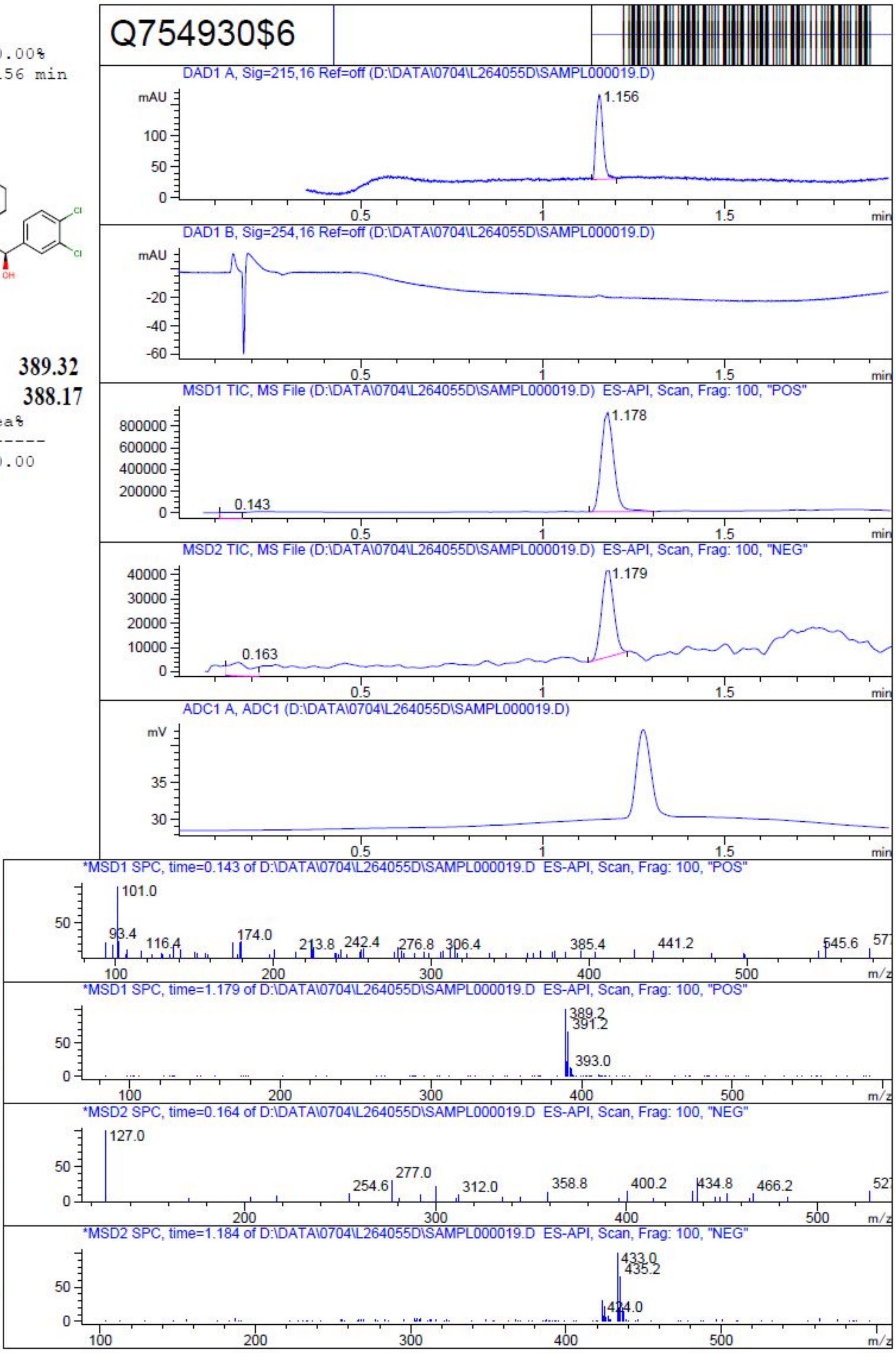

RT 0.143

RT 1.178

RT 0.163

RT 1.179

Enamine ID: PV-001919611983

MaxPeak: 96.42%
Ret_Time: 1.137 min

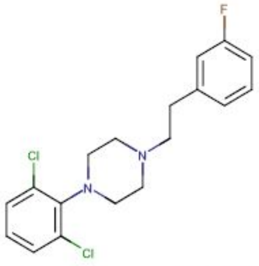

Mol Wt 353.26
Exact Mass 352.13

#	Time	Area%
1	0.889	1.30
2	1.137	96.42
3	1.194	2.28

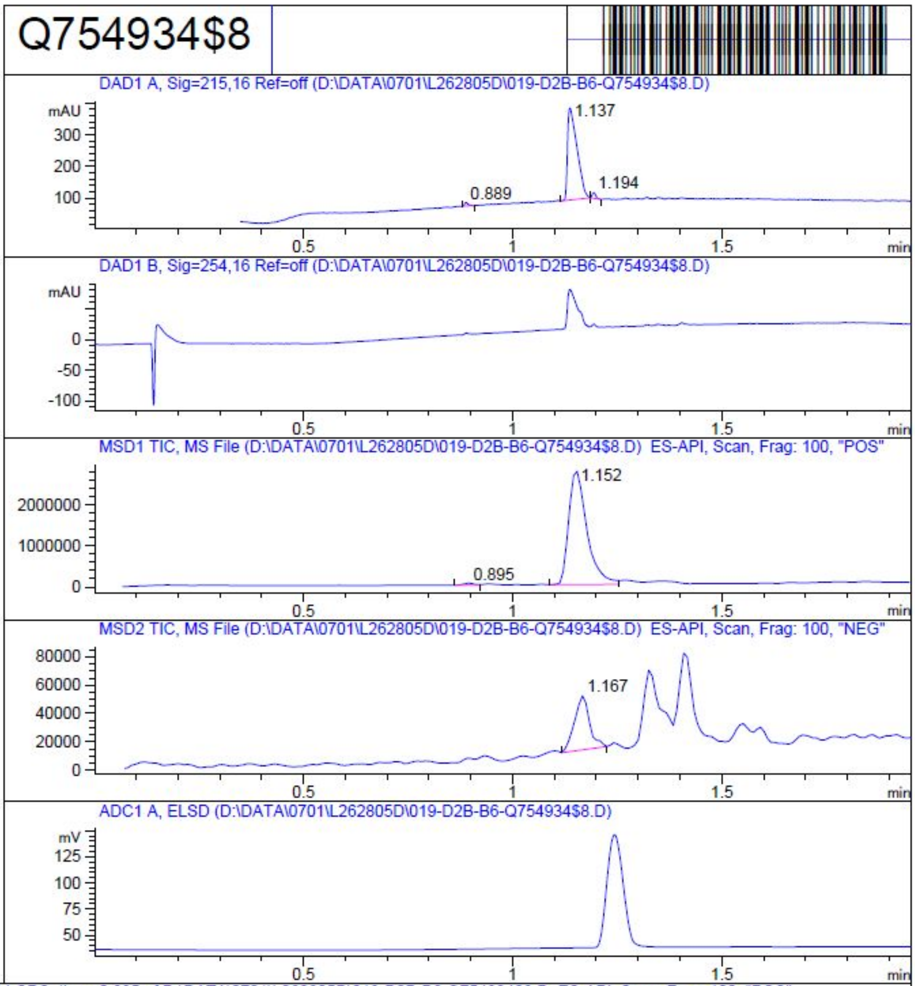

RT 0.895

RT 1.152

RT 1.167

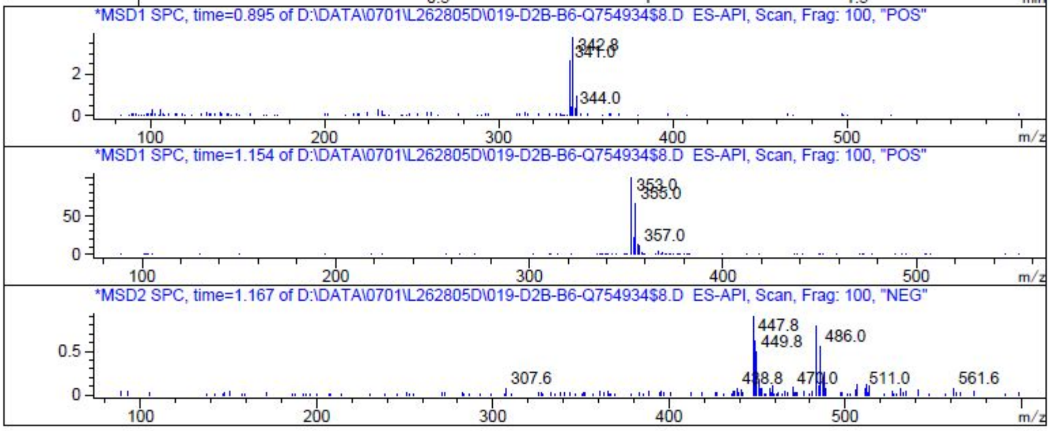

Enamine ID: PV-001918950467

MaxPeak: 100.00%
Ret_Time: 1.062 min

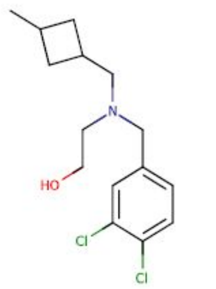

Mol Wt 302.24

Exact Mass 301.14

#	Time	Area%
1	1.062	100.00

RT 1.080

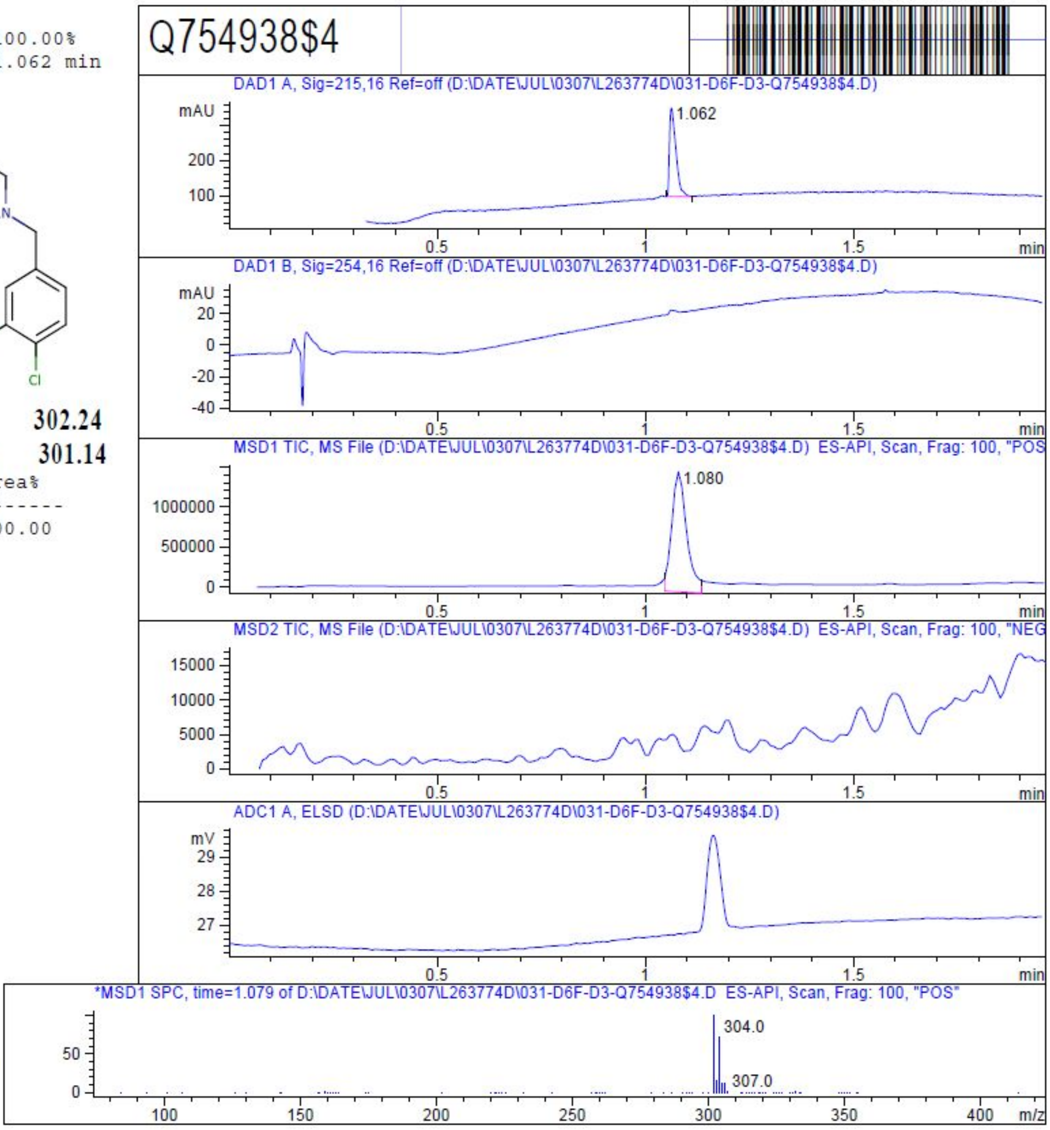

Enamine ID: PV-001918897538

MaxPeak: 100.00%
Ret_Time: 0.844 min

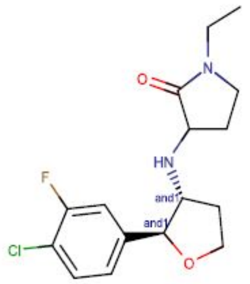

Mol Wt 326.79
Exact Mass 326.15

#	Time	Area%
1	0.844	100.00

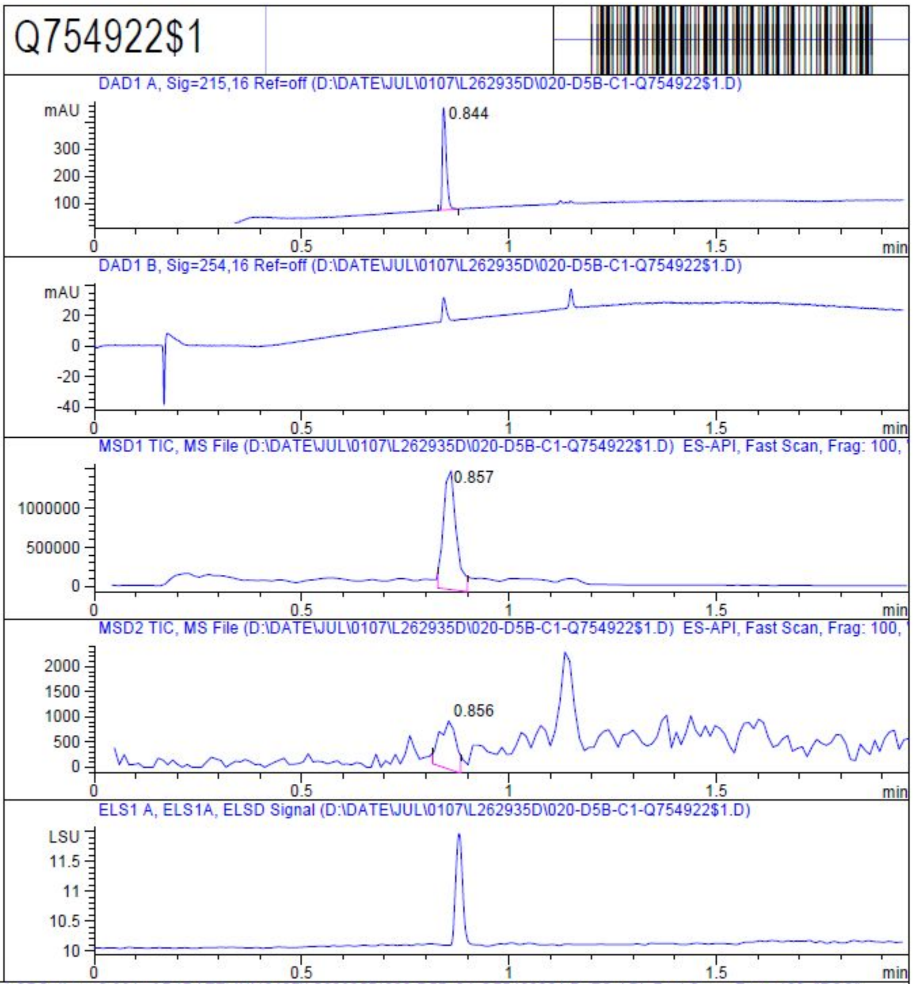

RT 0.857

RT 0.856

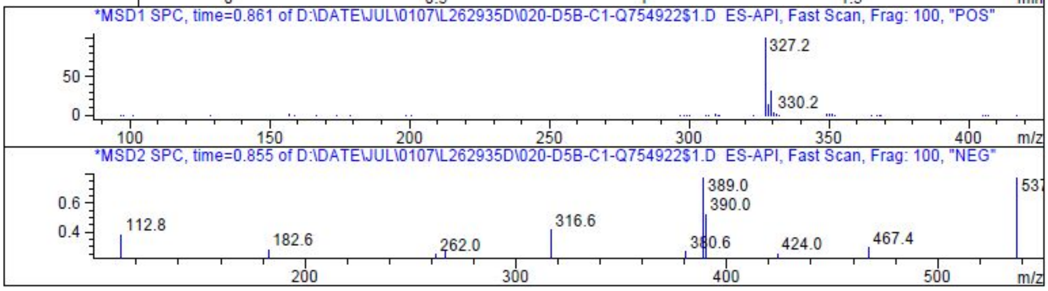

Enamine ID: PV-001921689112

MaxPeak: 98.45%
Ret_Time: 1.127 min

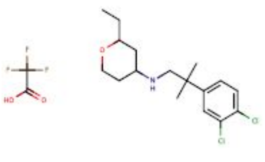

Mol Wt 444.32
Exact Mass 329.18

#	Time	Area%
1	1.088	1.55
2	1.127	98.45

Q754950$3

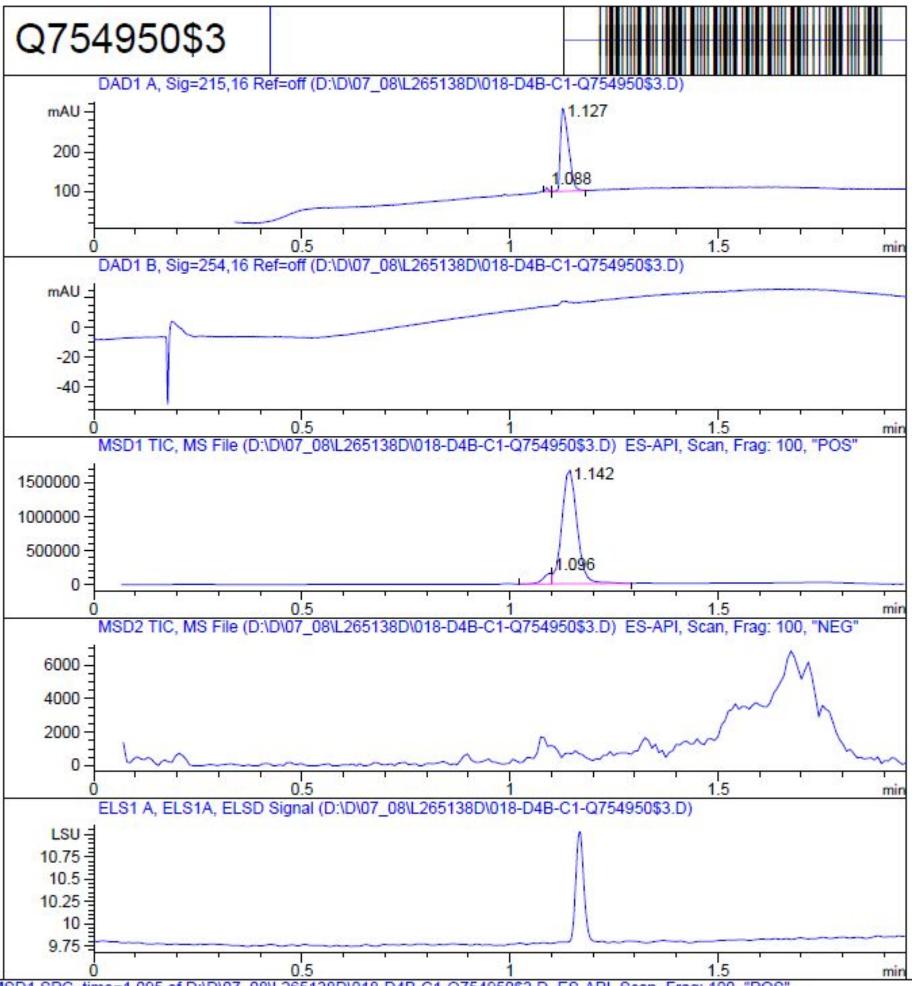

RT 1.096

RT 1.142

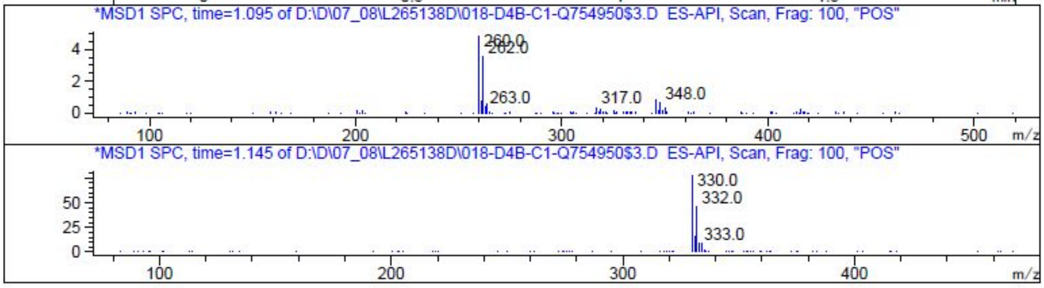

Enamine ID: PV-001923897672

```
MaxPeak:   96.55%
Ret_Time:  0.644 min
```

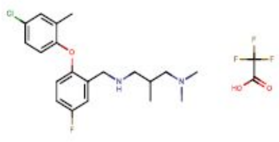

Mol Wt 478.91
Exact Mass 364.22

#	Time	Area%
1	0.644	96.55
2	0.959	3.45

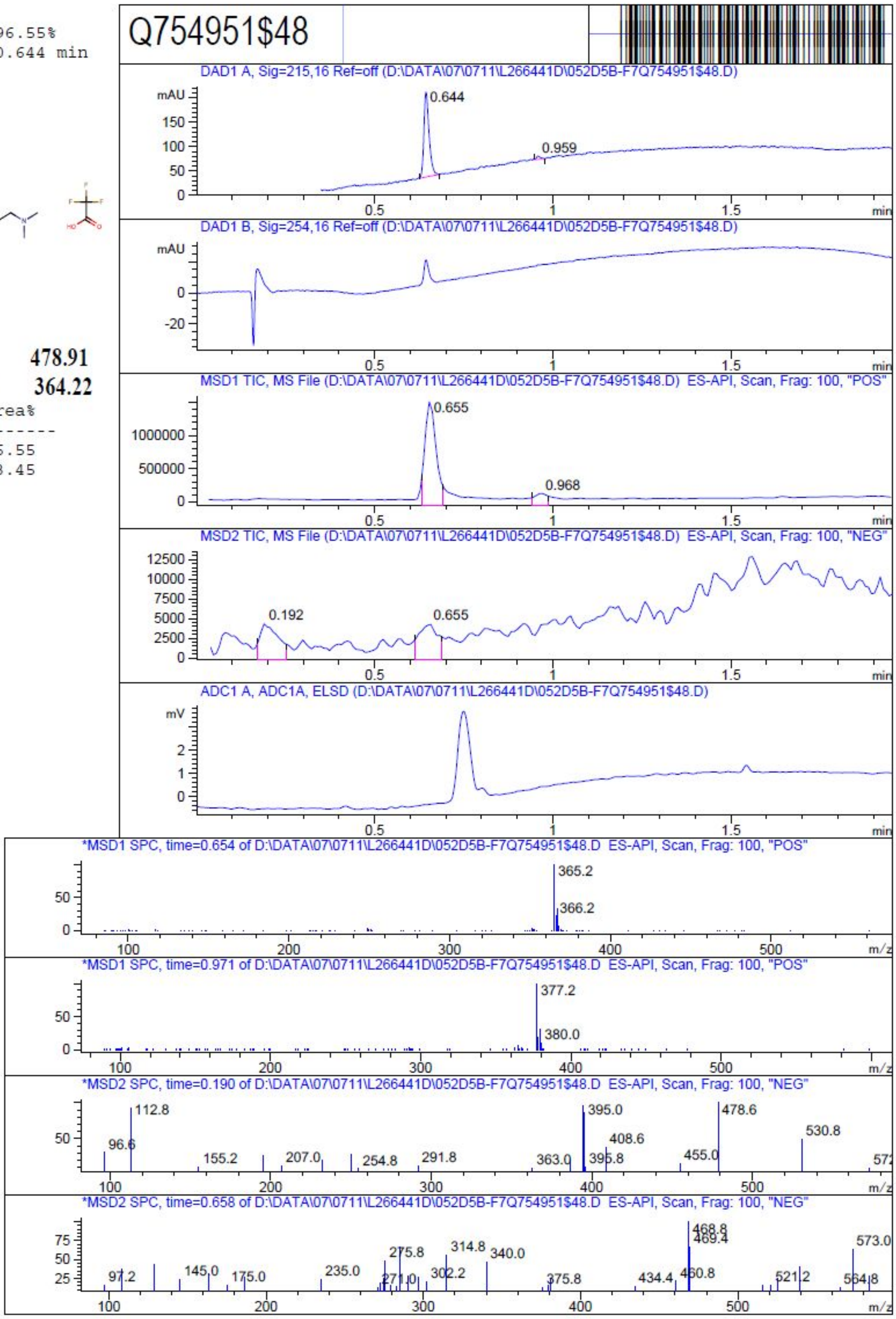

RT 0.655

RT 0.968

RT 0.192

RT 0.655

Enamine ID: PV-001925822957

MaxPeak: 100.00%
Ret_Time: 0.797 min

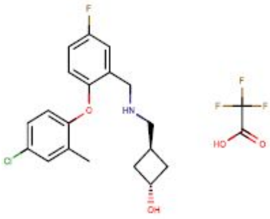

Mol Wt 463.85
Exact Mass 349.16

#	Time	Area%
1	0.797	100.00

RT 0.807

RT 0.193

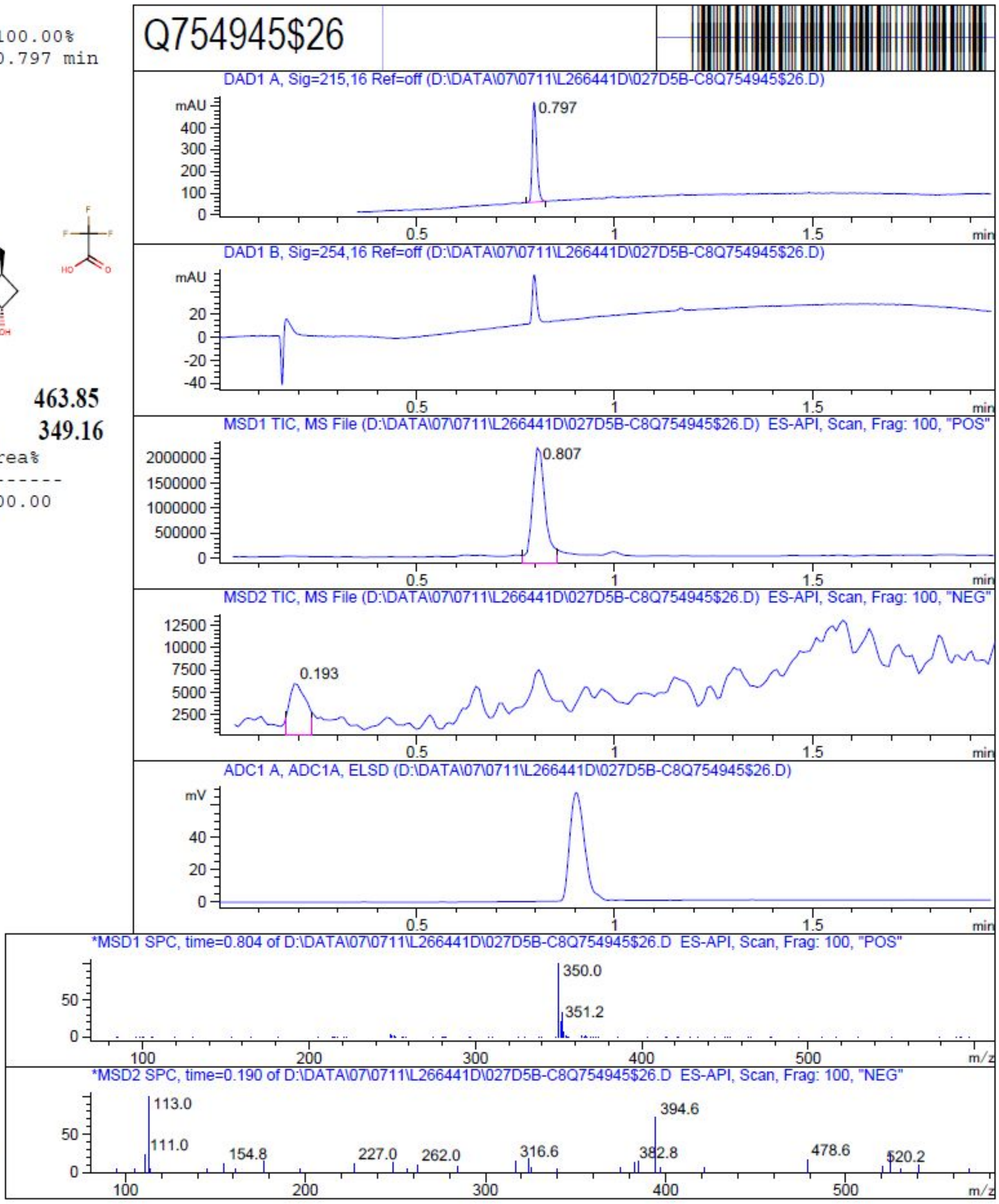

Enamine ID: PV-001926691632

MaxPeak: 93.45%
Ret_Time: 1.624 min

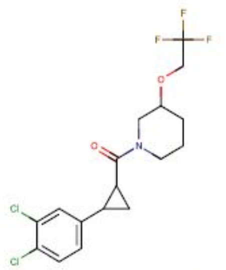

Mol Wt 396.23
Exact Mass 395.1

#	Time	Area%
1	1.592	6.55
2	1.624	93.45

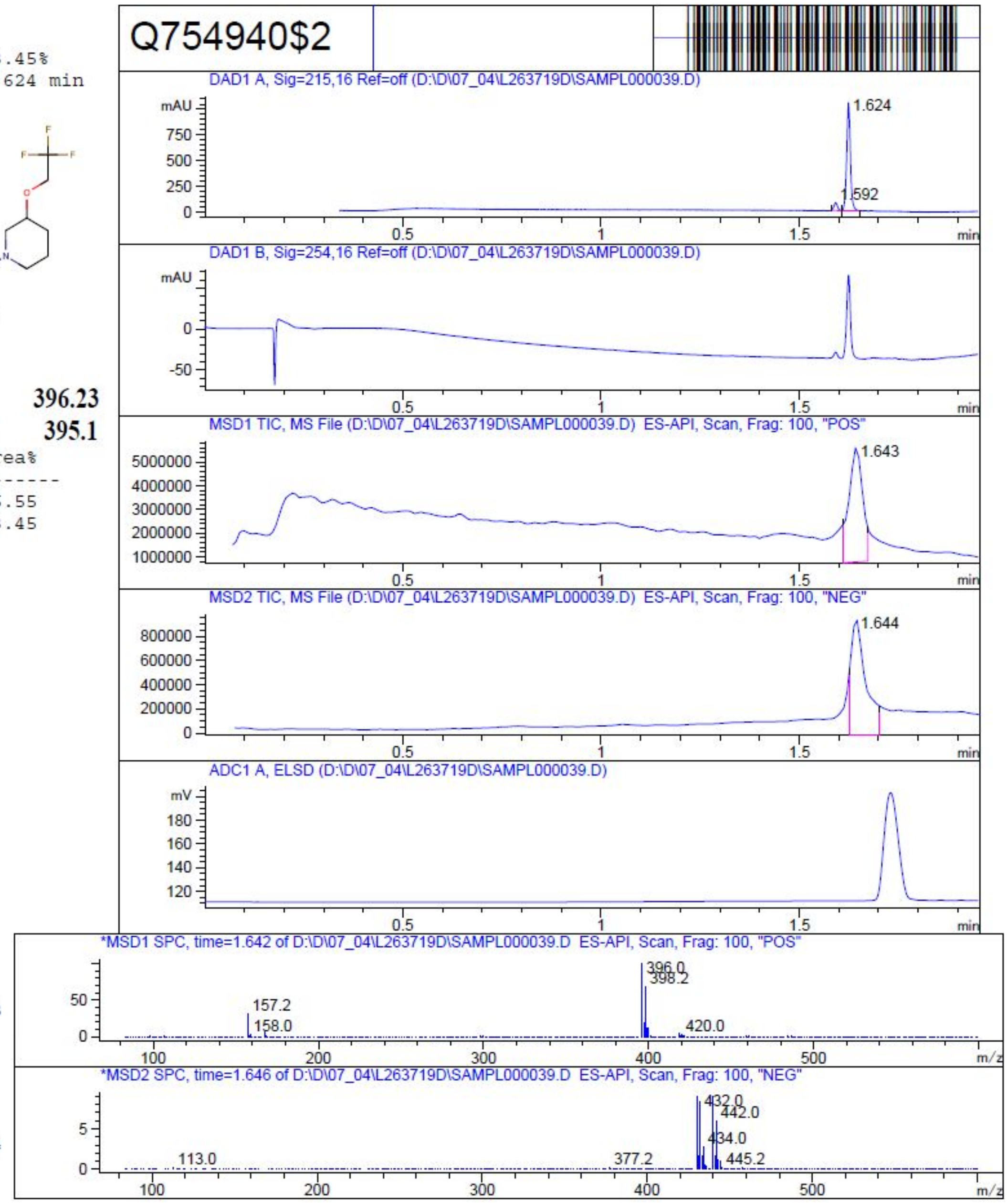

RT 1.643

RT 1.644

Enamine ID: Z1083362508

MaxPeak: 100.00%
Ret_Time: 1.058 min

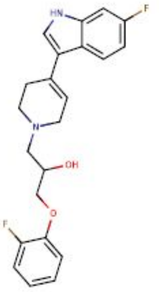

Mol Wt 384.42
Exact Mass 384.2

#	Time	Area%
1	1.058	100.00

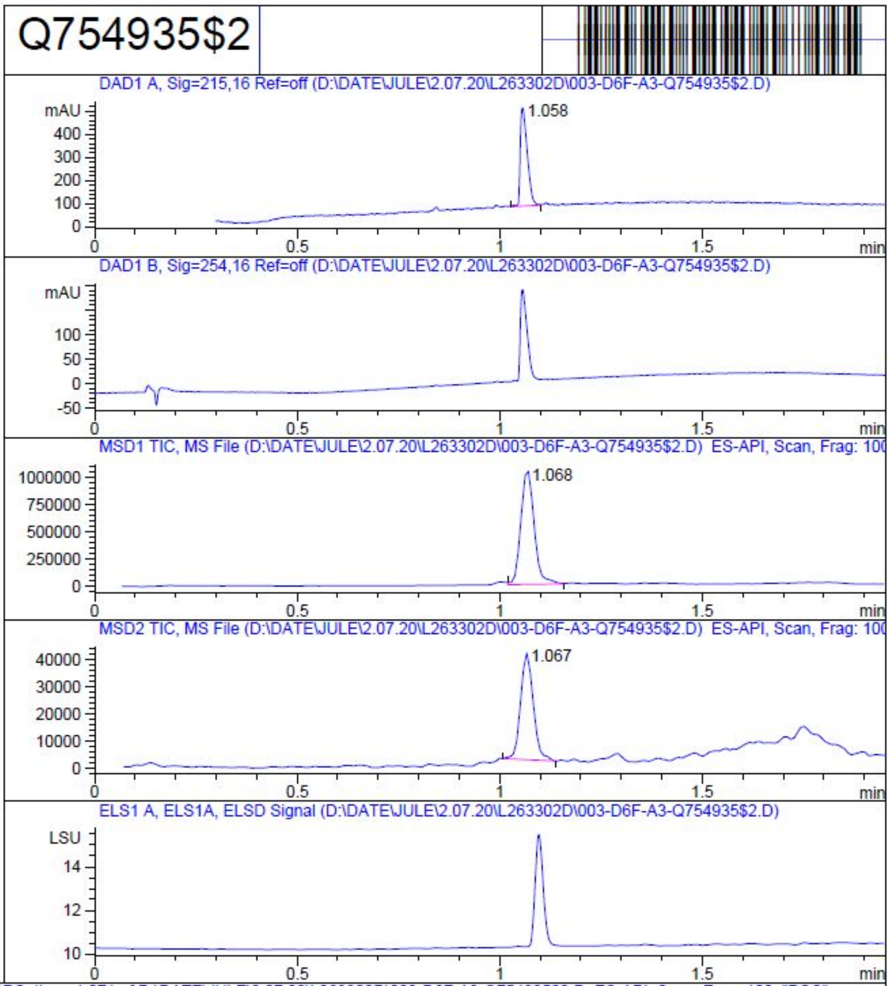

RT 1.068

RT 1.067

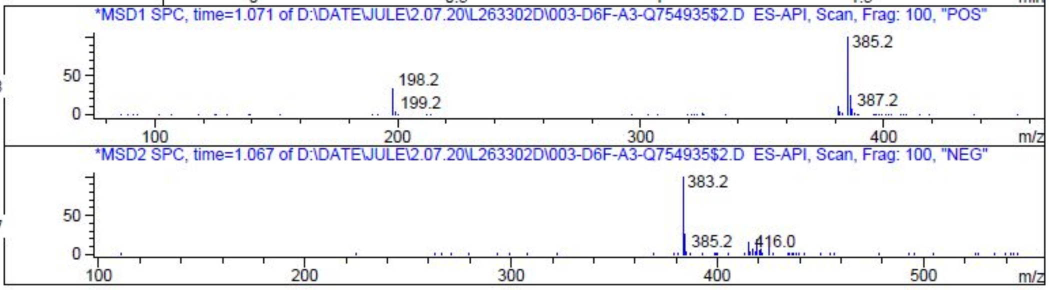

Enamine ID: Z1485114833

MaxPeak: 100.00%
Ret_Time: 1.083 min

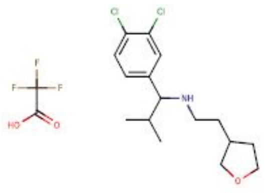

Mol Wt 430.29
Exact Mass 315.16

#	Time	Area%
1	1.083	100.00

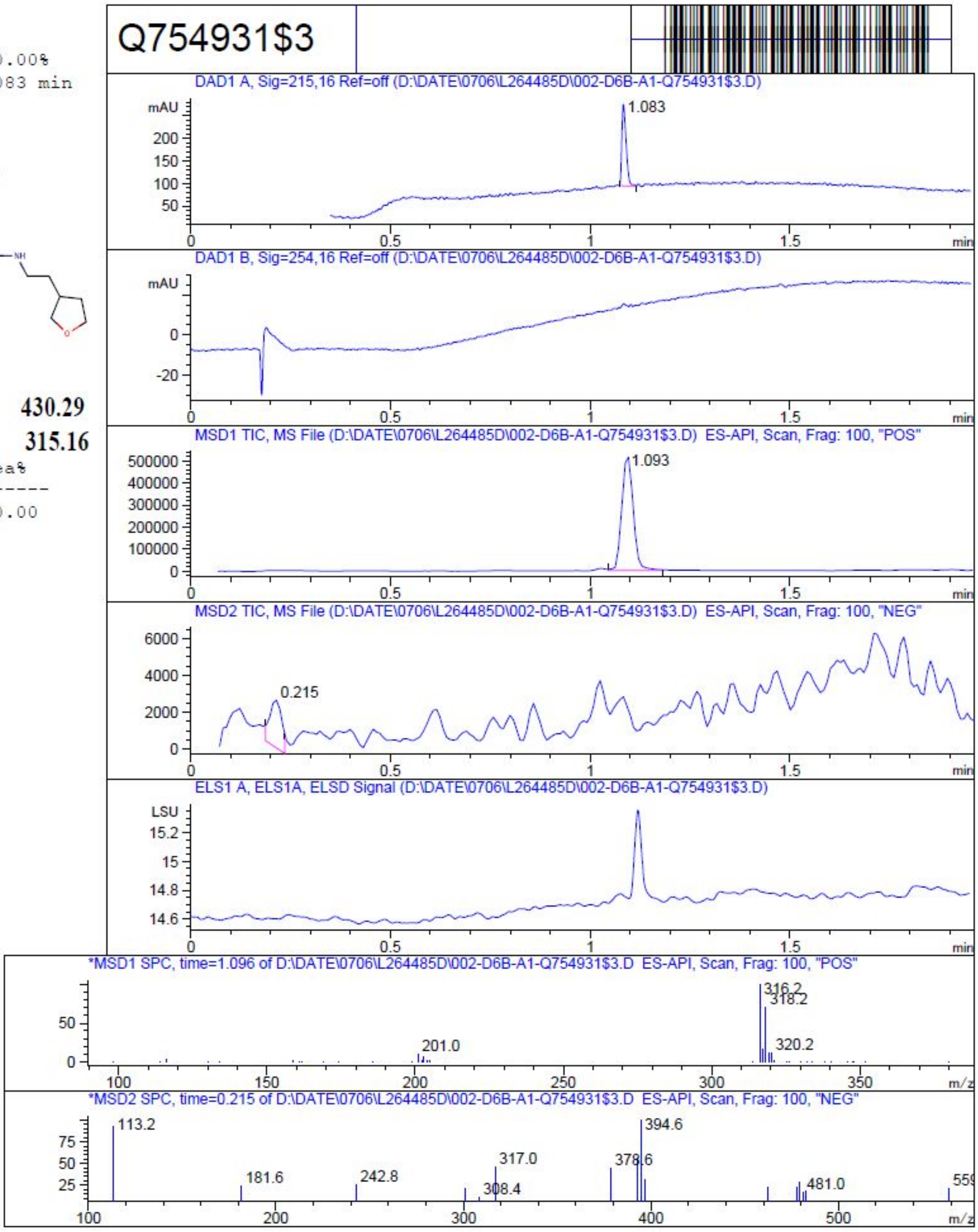

RT 1.093

RT 0.215

Enamine ID: Z1663514926

MaxPeak: 98.94%
Ret_Time: 1.037 min

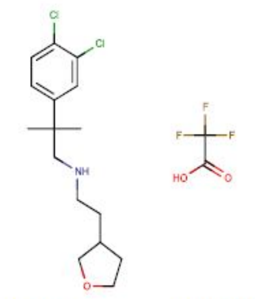

Mol Wt 430.29
Exact Mass 315.16

#	Time	Area%
1	0.694	1.06
2	1.037	98.94

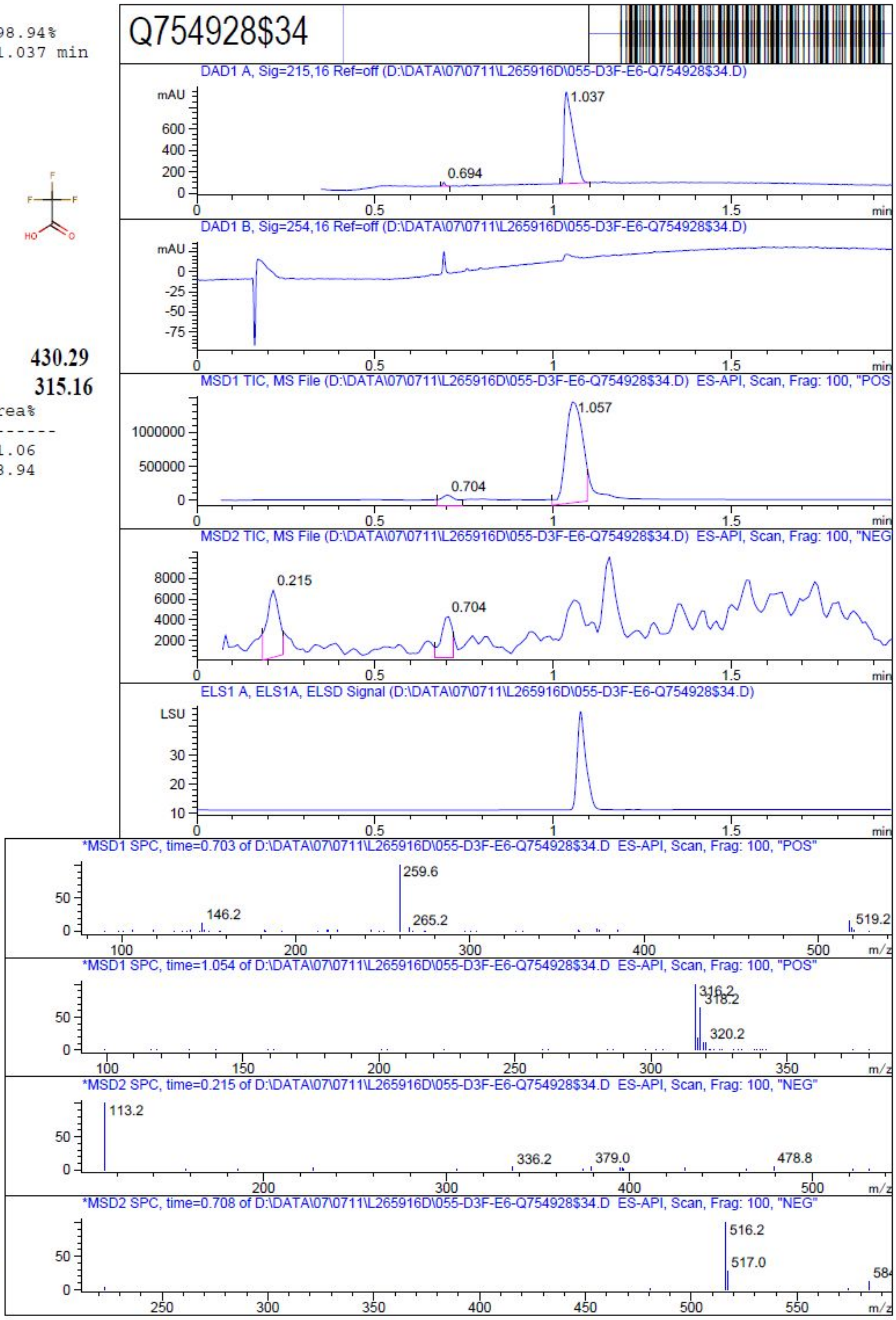

Enamine ID: Z1783772638

MaxPeak: 90.57%
Ret_Time: 1.071 min

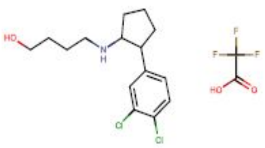

Mol Wt 416.26
Exact Mass 301.14

#	Time	Area%
1	1.050	5.62
2	1.071	90.57
3	1.186	3.81

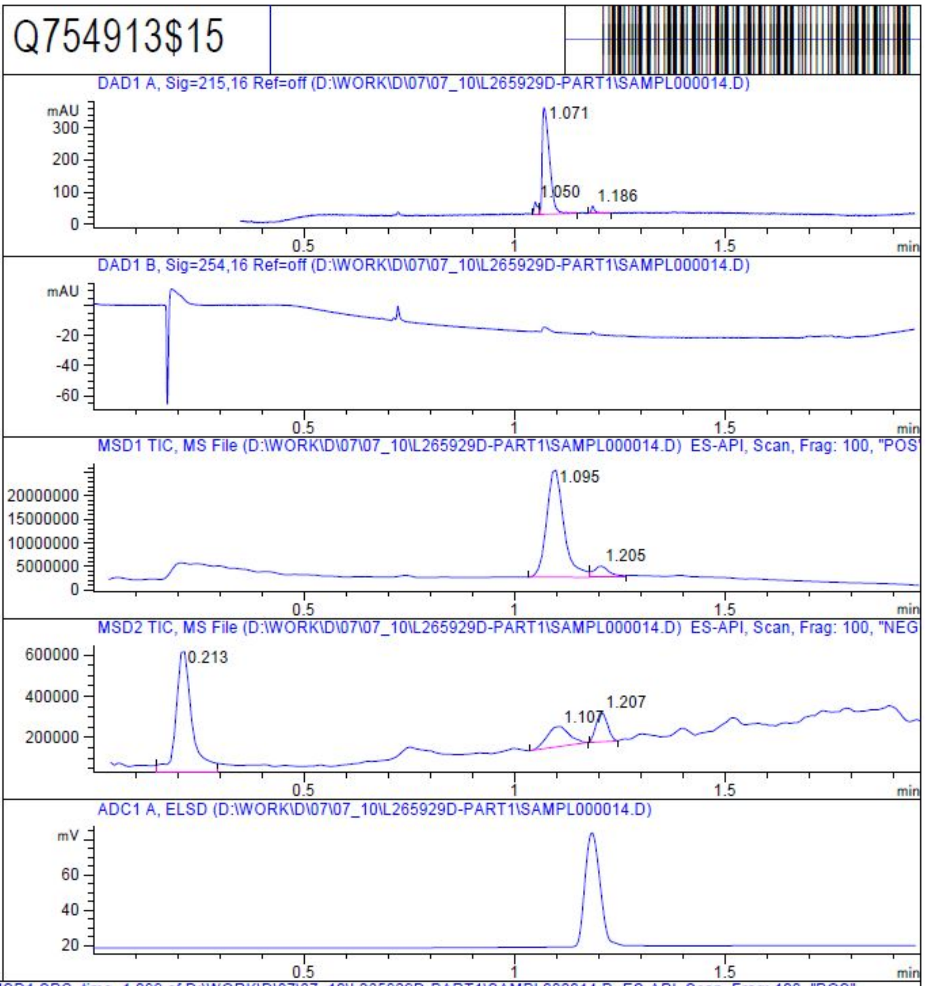

RT 1.095

RT 1.205

RT 0.213

RT 1.107

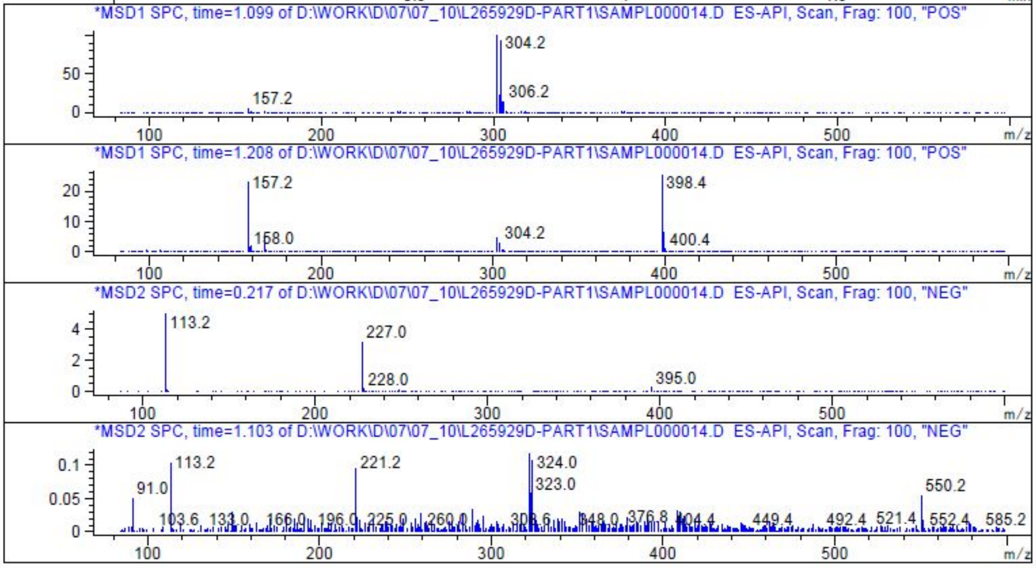

Enamine ID: Z1904820040

MaxPeak: 100.00%
Ret_Time: 1.105 min

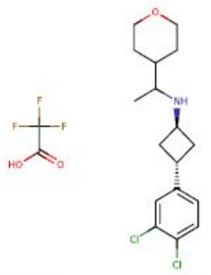

Mol Wt 442.3
Exact Mass 327.16

#	Time	Area%
1	1.105	100.00

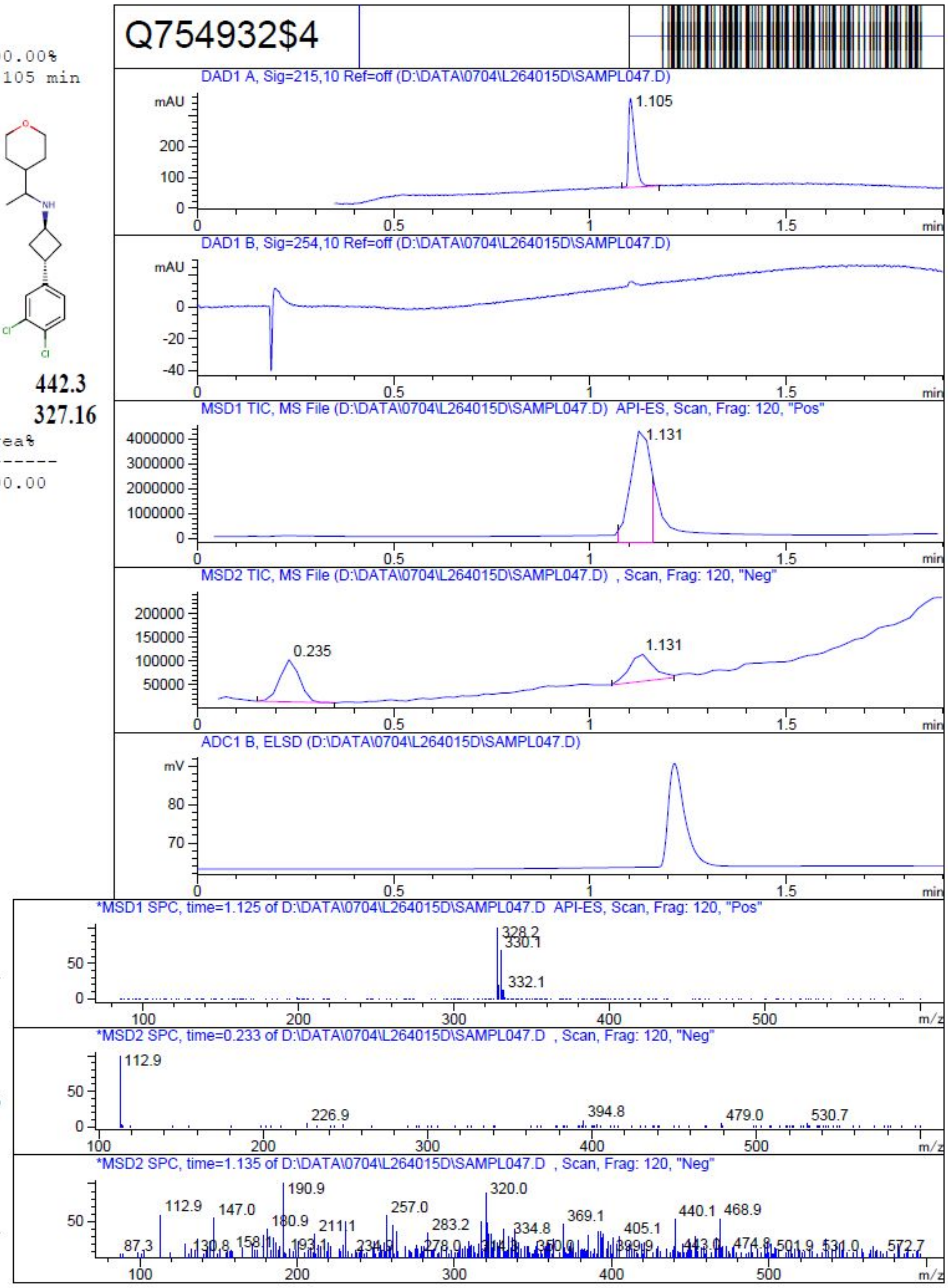

RT 1.131

RT 0.235

RT 1.131

Enamine ID: Z1986623199

MaxPeak: 98.86%
Ret_Time: 0.589 min

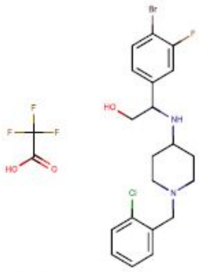

Mol Wt 555.79
Exact Mass 442.11

#	Time	Area%
1	0.589	98.86
2	0.874	1.14

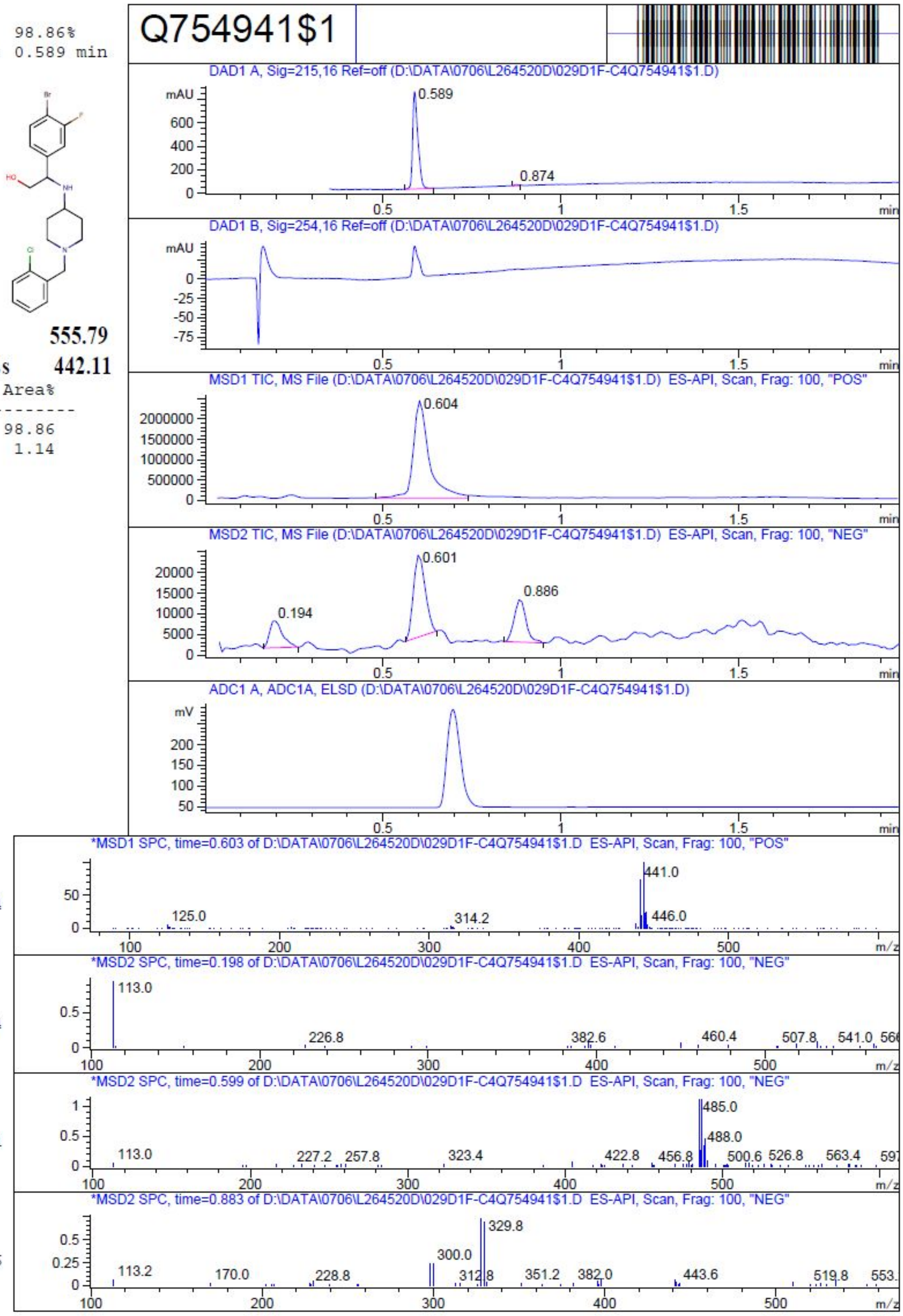

Enamine ID: Z2201777668

MaxPeak: 100.00%
Ret_Time: 1.075 min

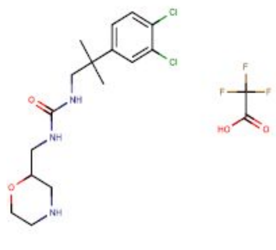

Mol Wt 474.3
Exact Mass 359.15

#	Time	Area%
1	1.075	100.00

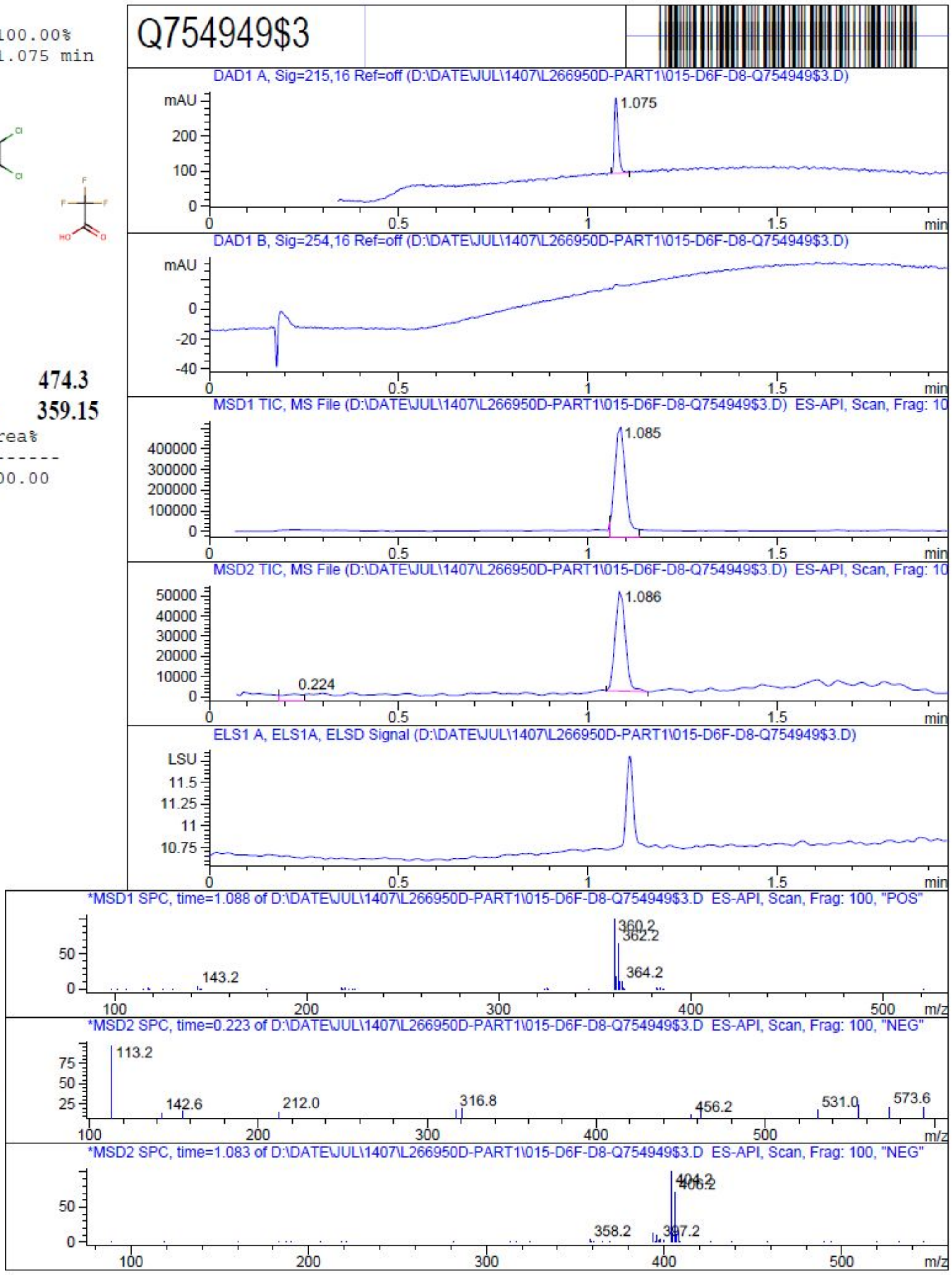

RT 1.085

RT 0.224

RT 1.086

Enamine ID: Z2361435743

MaxPeak: 100.00%
Ret_Time: 1.084 min

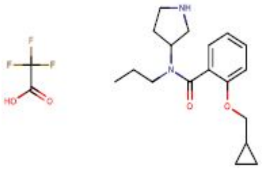

Mol Wt 416.44
Exact Mass 302.24

#	Time	Area%
1	1.084	100.00

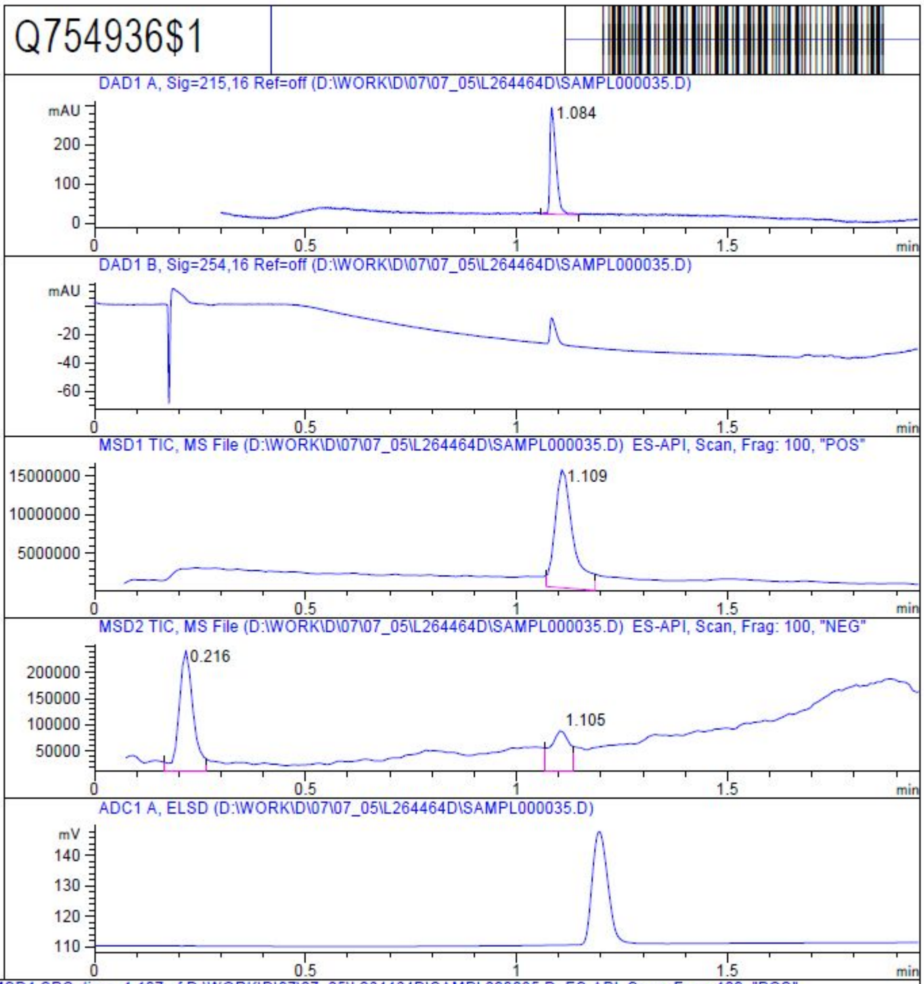

RT 1.109

RT 0.216

RT 1.105

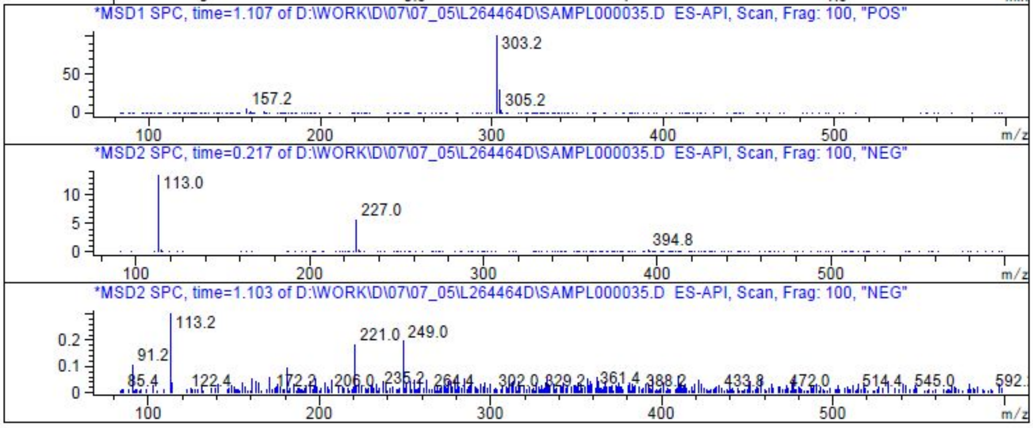

Enamine ID: Z2393213853

MaxPeak: 97.44%
Ret_Time: 1.081 min

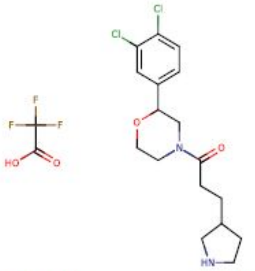

Mol Wt 471.3

Exact Mass 356.14

#	Time	Area%
1	0.967	2.56
2	1.081	97.44

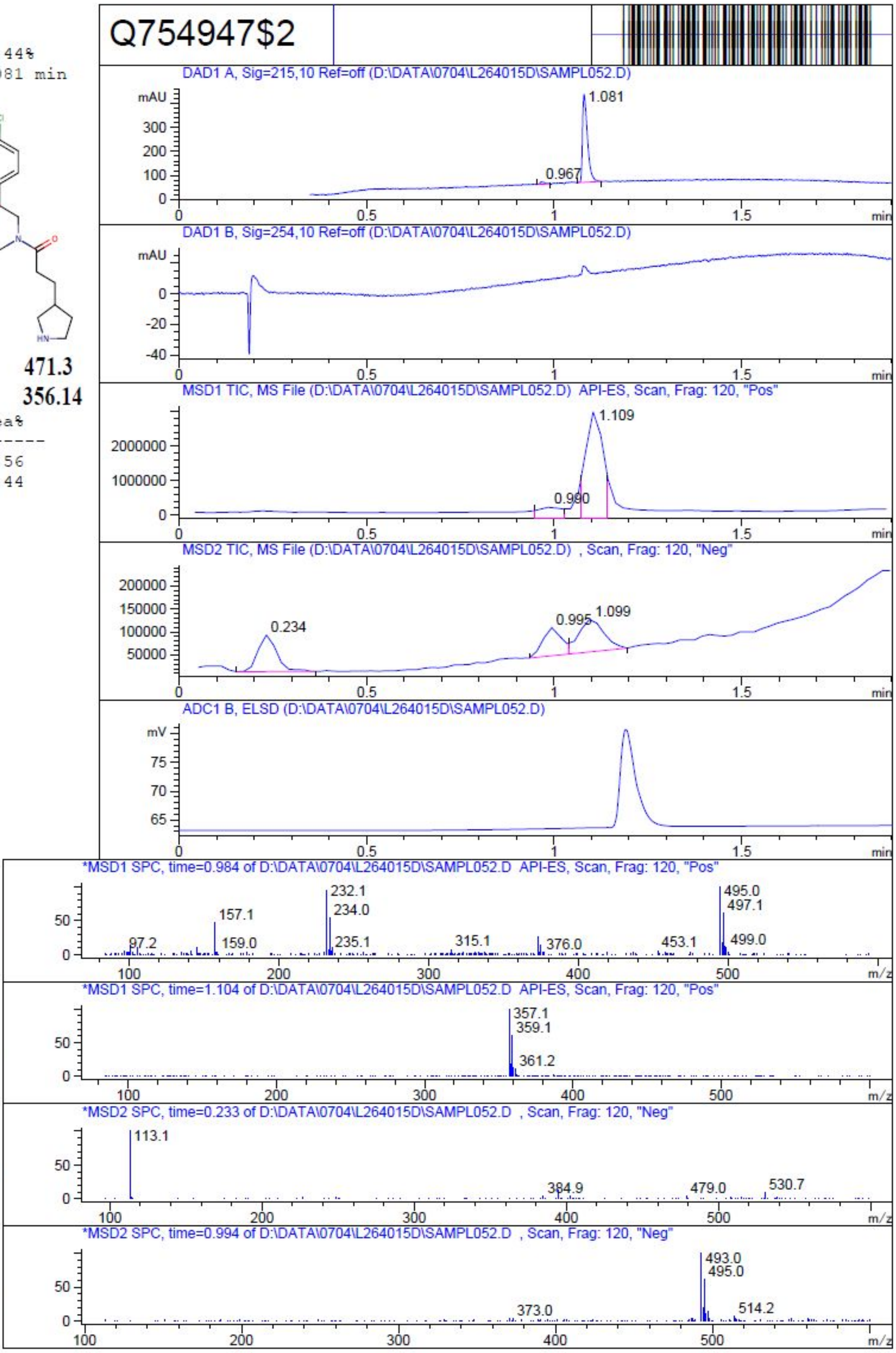

RT 0.990

RT 1.109

RT 0.234

RT 0.995

Enamine ID: Z2436746713

MaxPeak: 98.77%
Ret_Time: 0.929 min

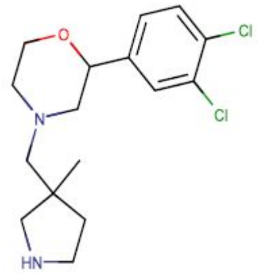

Mol Wt 329.26
Exact Mass 328.15

#	Time	Area%
1	0.929	98.77
2	1.143	1.23

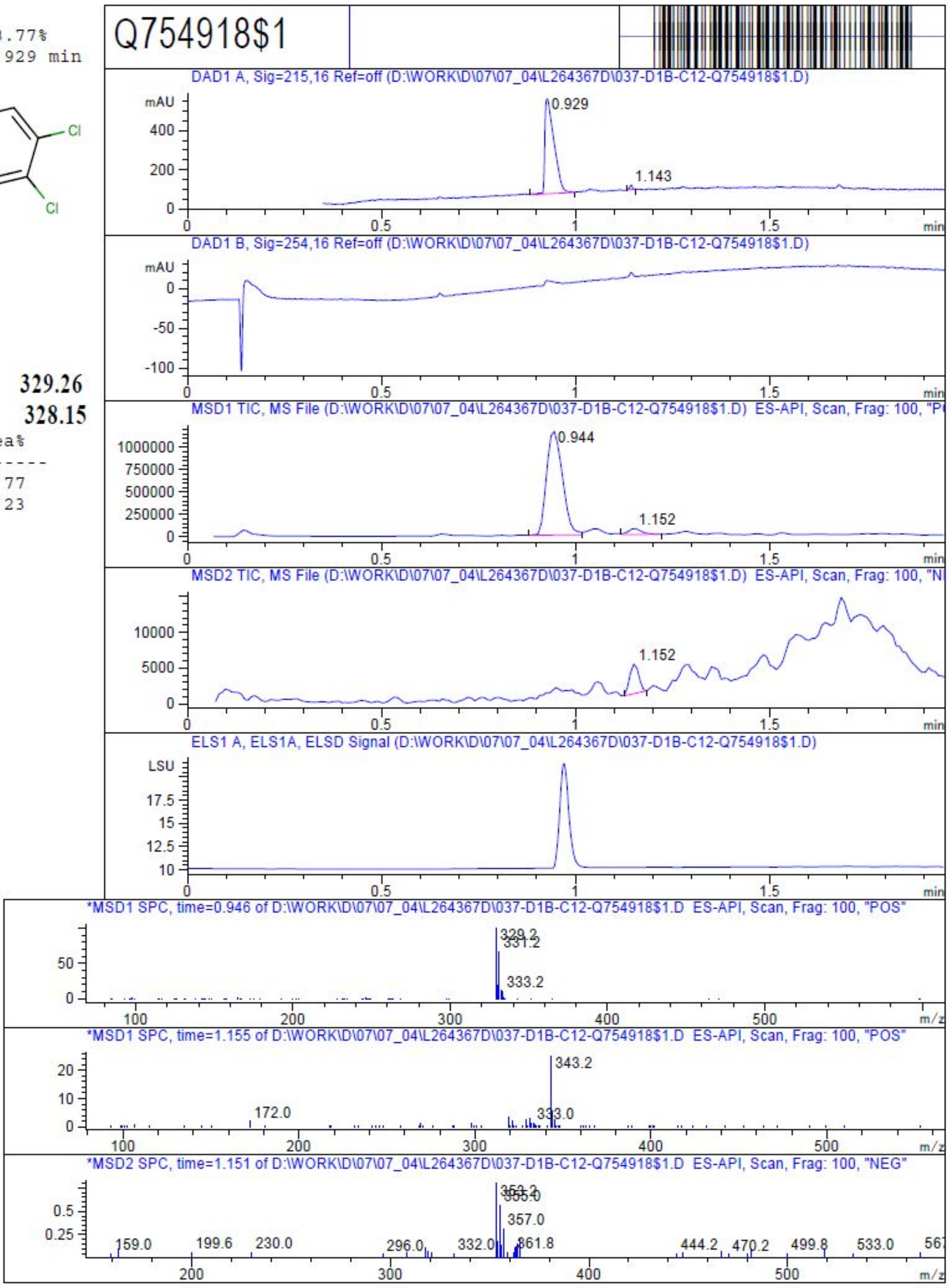

RT 0.944

RT 1.152

RT 1.152

Enamine ID: Z2444828348

MaxPeak: 96.66%
Ret_Time: 1.026 min

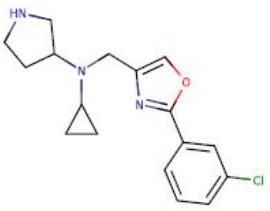

Mol Wt 317.81
Exact Mass 317.16

#	Time	Area%
1	0.749	3.34
2	1.026	96.66

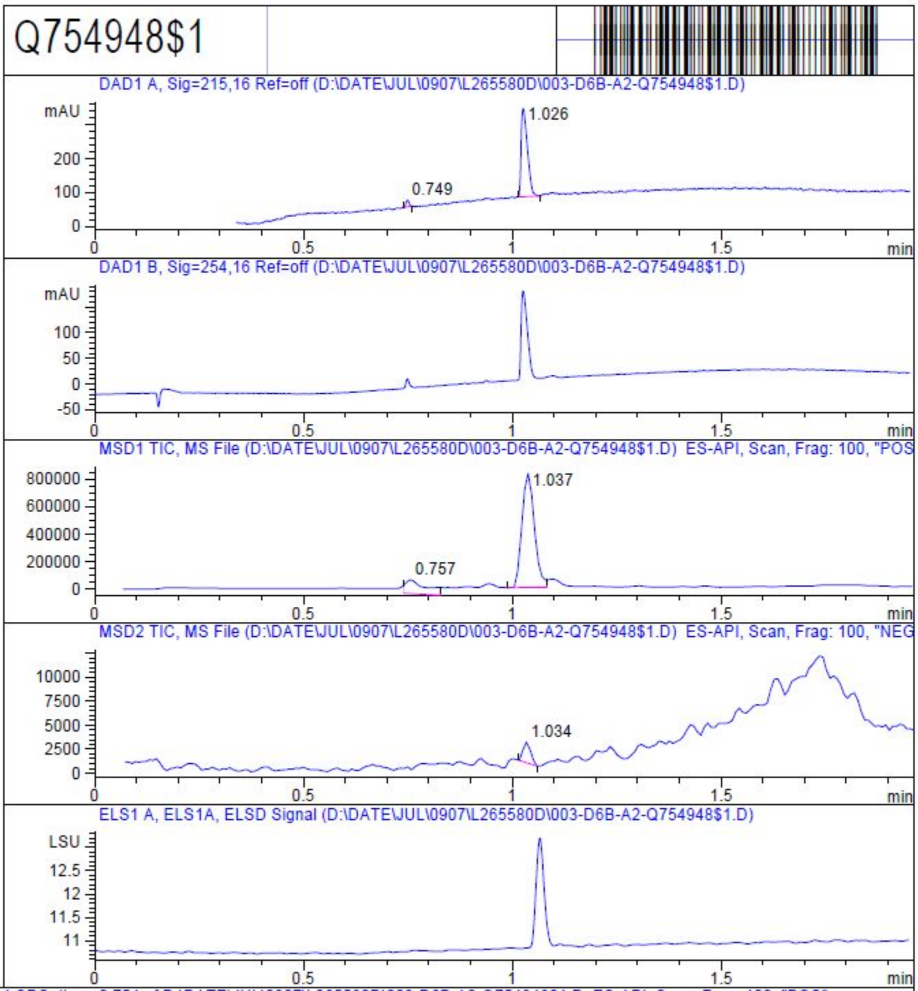

RT 0.757

RT 1.037

RT 1.034

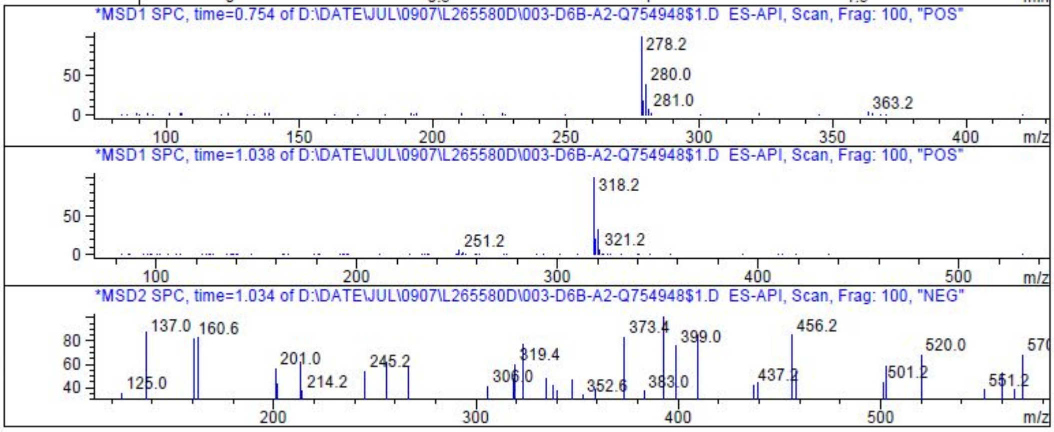

Enamine ID: Z2447436630

MaxPeak: 98.13%
Ret_Time: 0.882 min

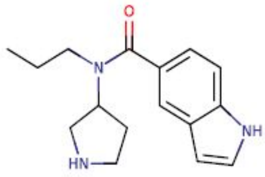

Mol Wt 271.36
Exact Mass 271.2

#	Time	Area%
1	0.882	98.13
2	1.445	1.87

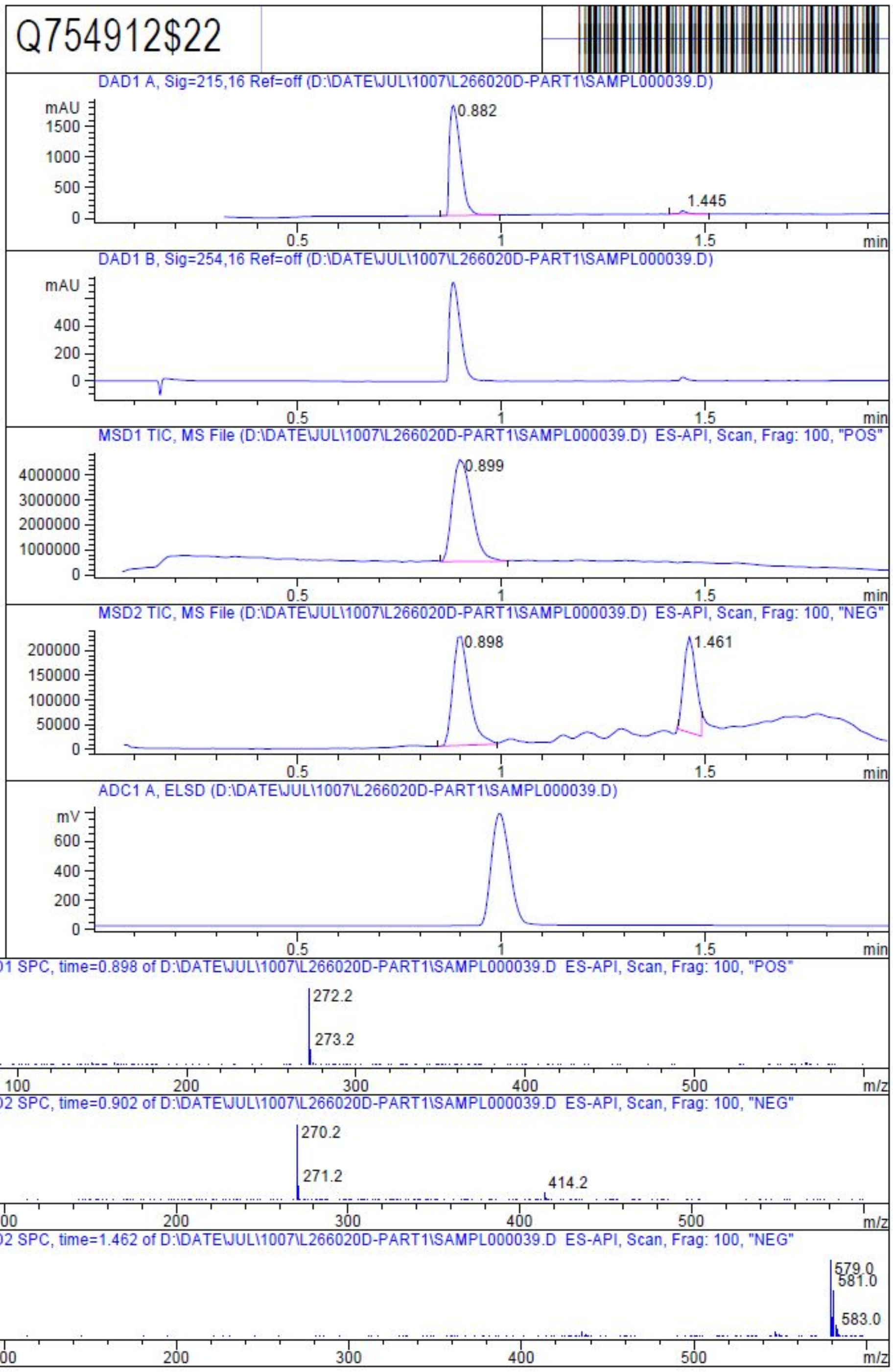

Enamine ID: Z2488563933

MaxPeak: 100.00%
Ret_Time: 0.993 min

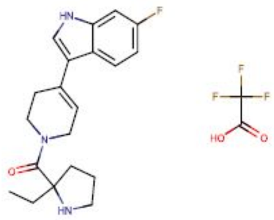

Mol Wt 455.45
Exact Mass 341.23

#	Time	Area%
1	0.993	100.00

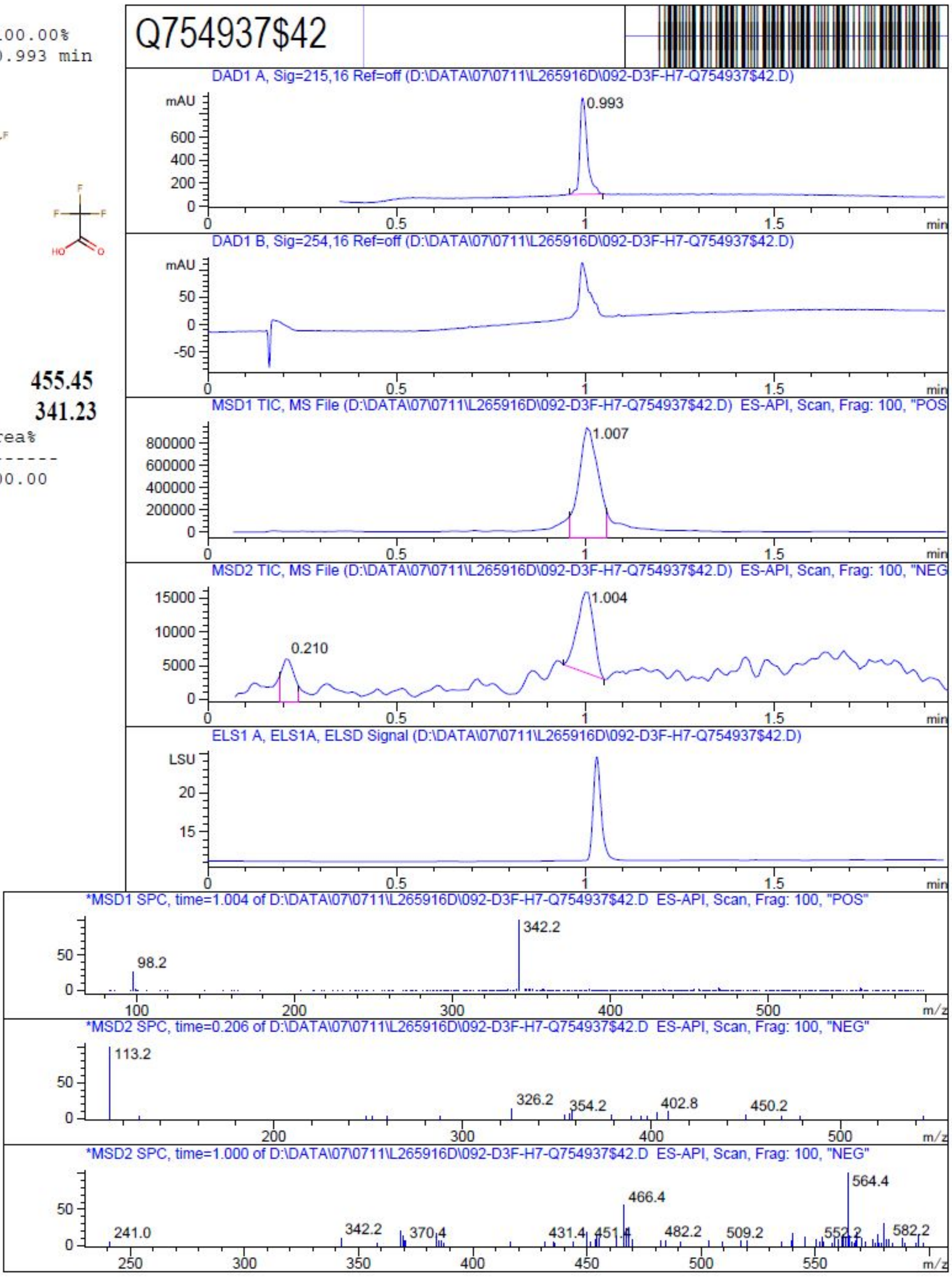

Enamine ID: Z2488796202

MaxPeak: 96.69%
Ret_Time: 1.047 min

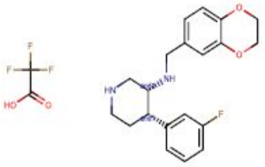

Mol Wt 456.43
Exact Mass 342.21

#	Time	Area%
1	1.047	96.69
2	1.114	3.31

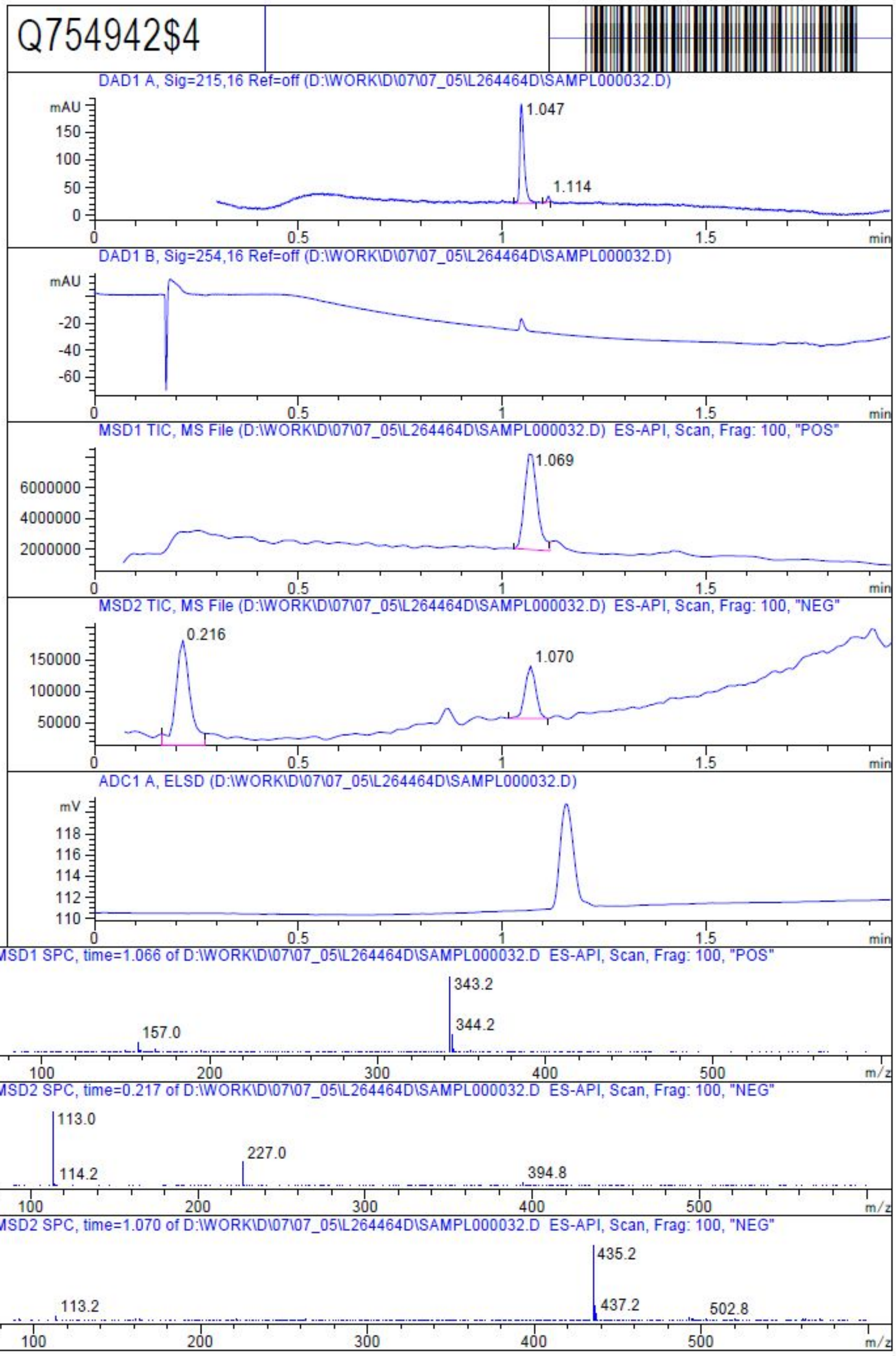

Enamine ID: Z2569757498

MaxPeak: 100.00%
Ret_Time: 1.040 min

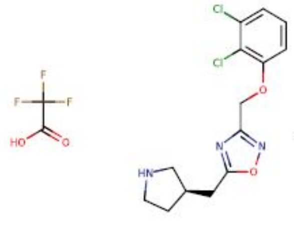

Mol Wt 442.22
Exact Mass 327.07

#	Time	Area%
1	1.040	100.00

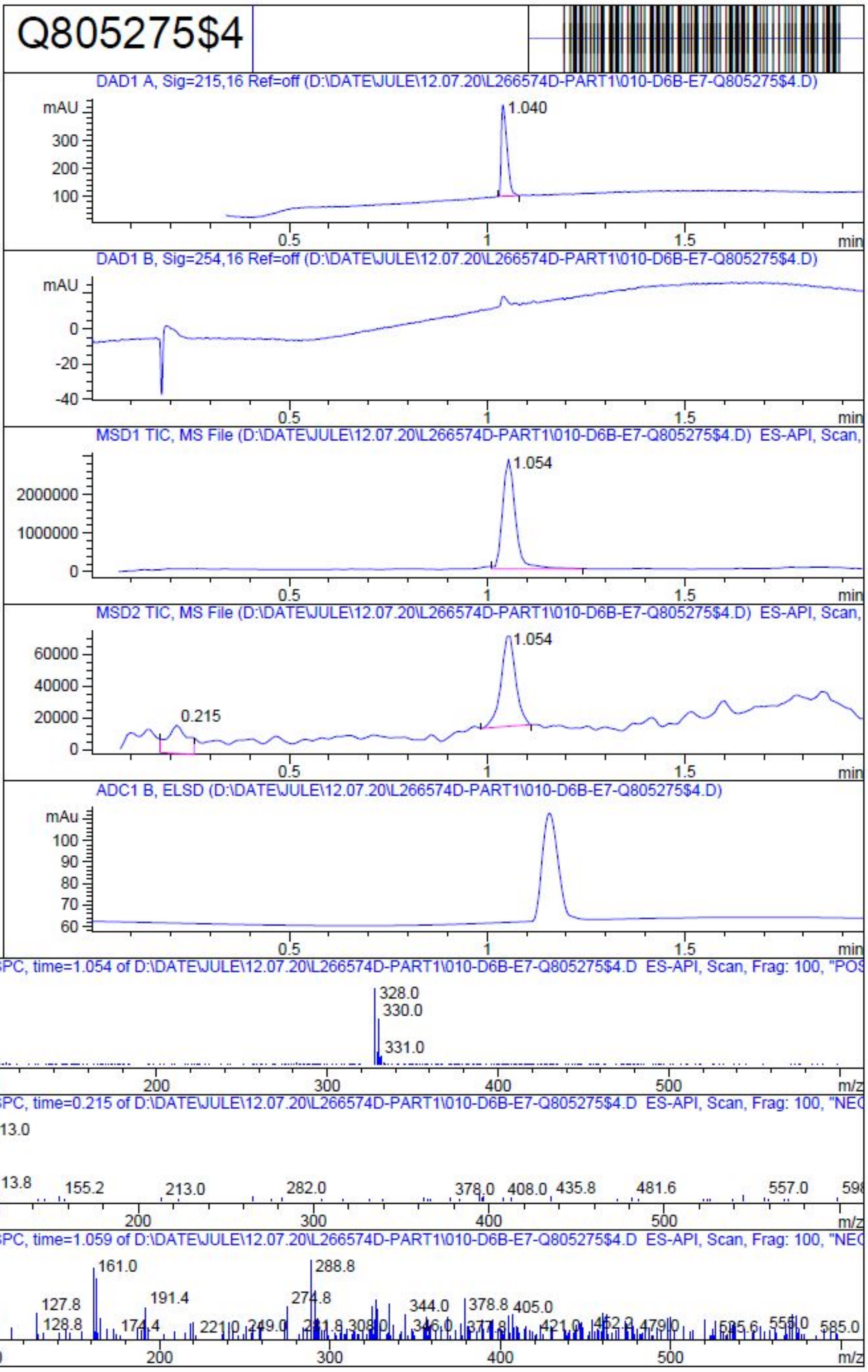

Enamine ID: Z2617873335

MaxPeak: 96.34%
Ret_Time: 1.072 min

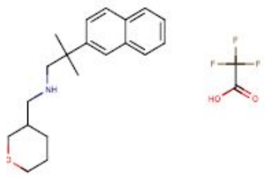

Mol Wt 411.46
Exact Mass 297.26

#	Time	Area%
1	0.983	3.66
2	1.072	96.34

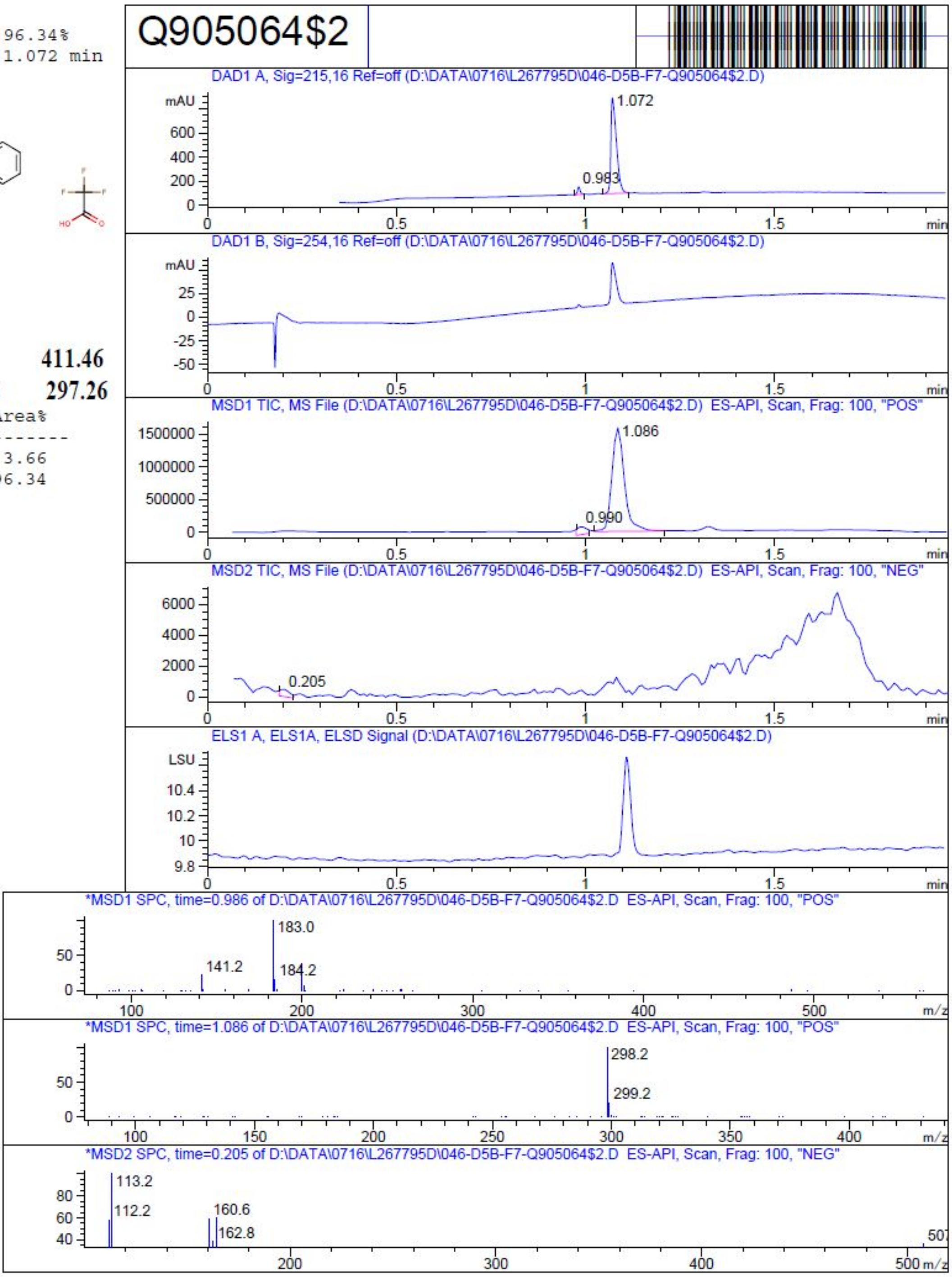

RT 0.990

RT 1.086

RT 0.205

Enamine ID: Z2698567135

MaxPeak: 98.46%
Ret_Time: 1.134 min

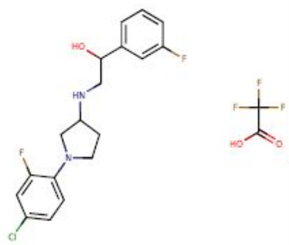

Mol Wt 466.83
Exact Mass 352.15

#	Time	Area%
1	1.134	98.46
2	1.182	1.54

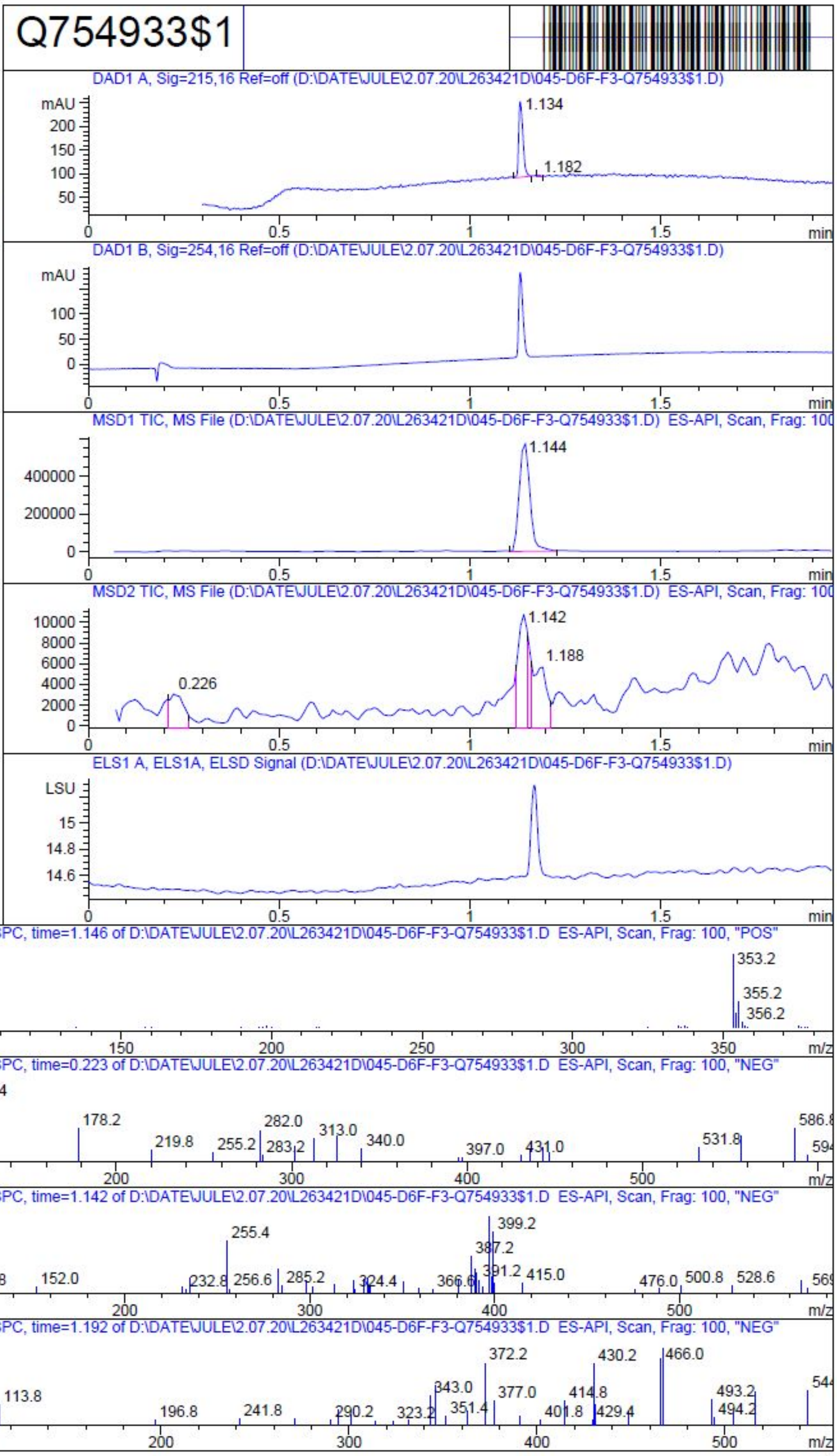

Enamine ID: Z2722622816

MaxPeak: 100.00%
Ret_Time: 1.046 min

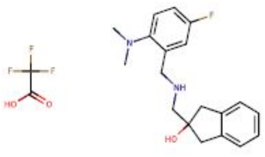

Mol Wt 428.42
Exact Mass 314.22

#	Time	Area%
1	1.046	100.00

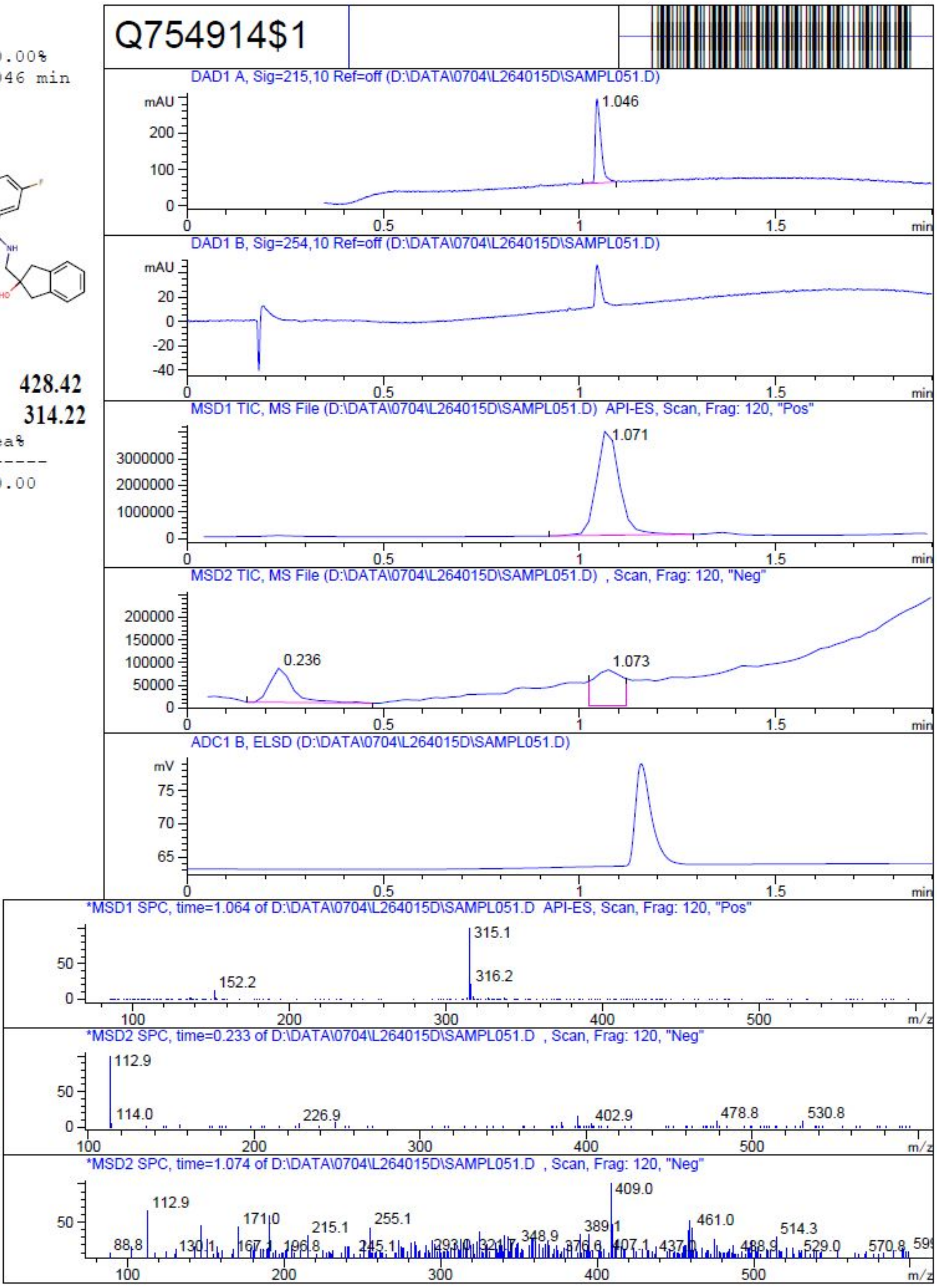

RT 1.071

RT 0.236

RT 1.073

Enamine ID: Z2769565776

MaxPeak: 100.00%
Ret_Time: 1.025 min

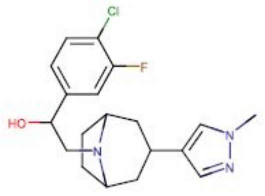

Mol Wt 363.86
Exact Mass 363.19

#	Time	Area%
1	1.025	100.00

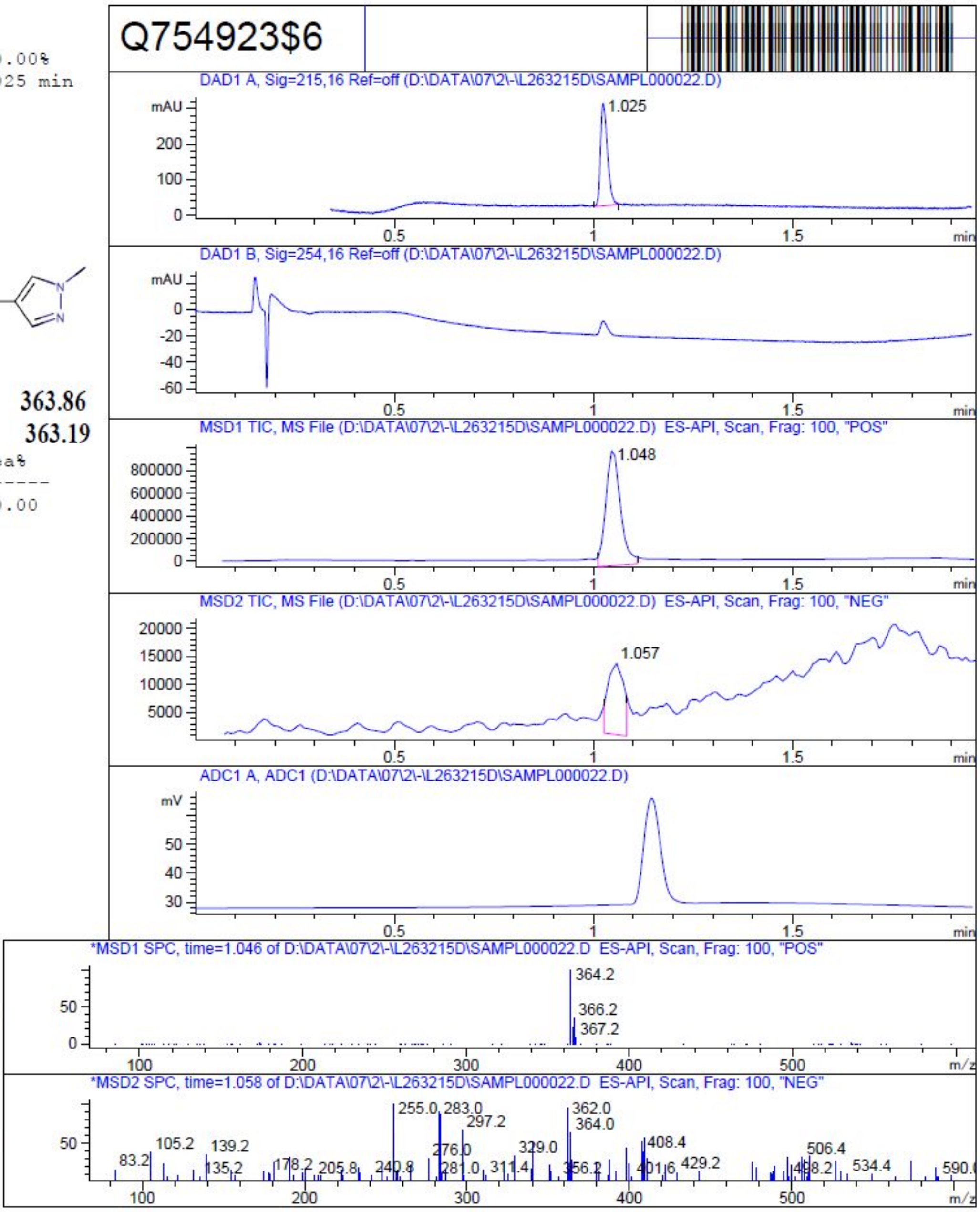

RT 1.048

RT 1.057

Enamine ID: Z2843312692

MaxPeak: 100.00%
Ret_Time: 1.076 min

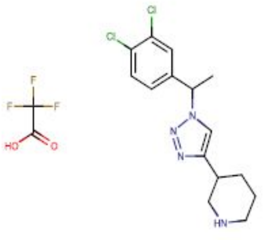

Mol Wt 439.26
Exact Mass 324.12

#	Time	Area%
1	1.076	100.00

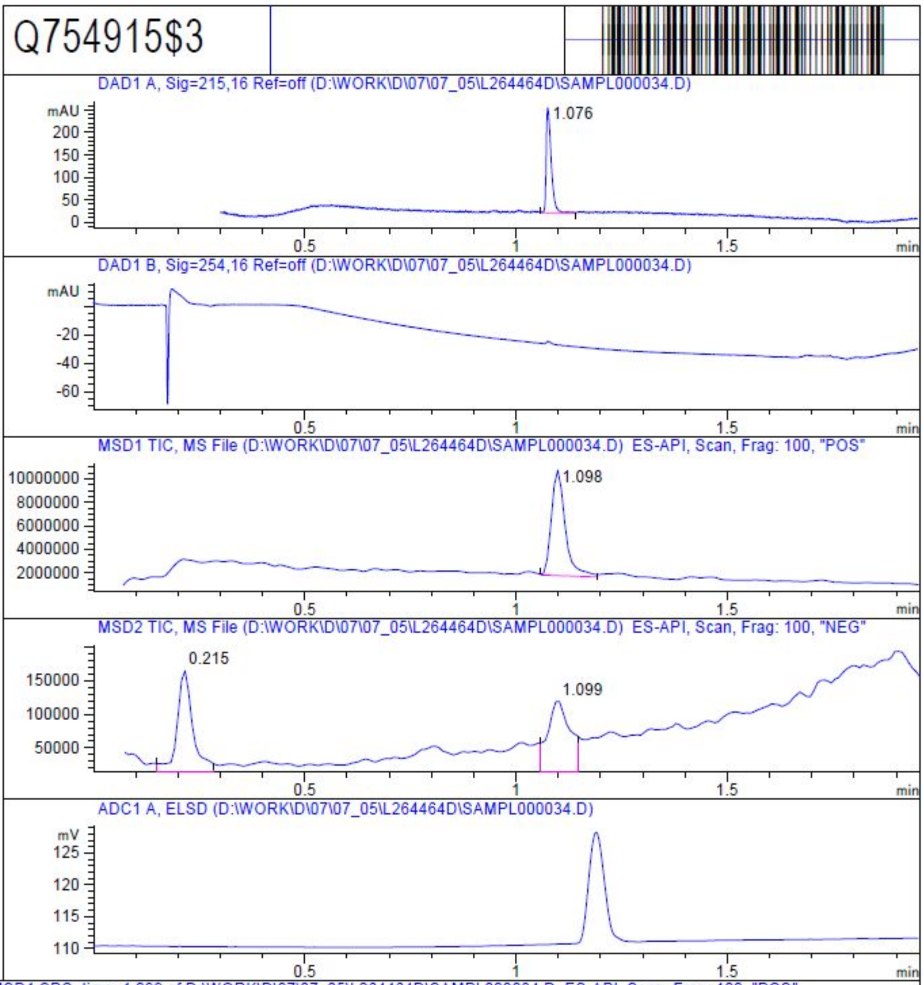

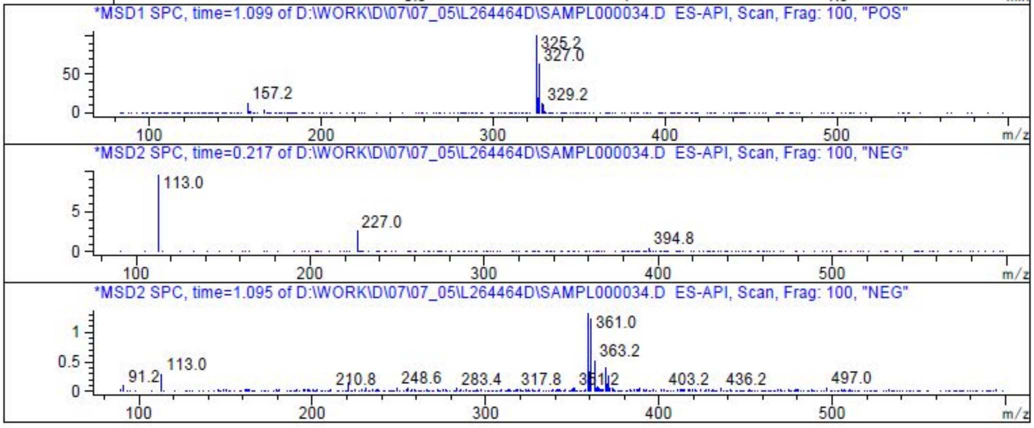

RT 1.098

RT 0.215

RT 1.099

Enamine ID: Z2846041003

MaxPeak: 97.54%
Ret_Time: 1.072 min

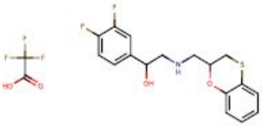

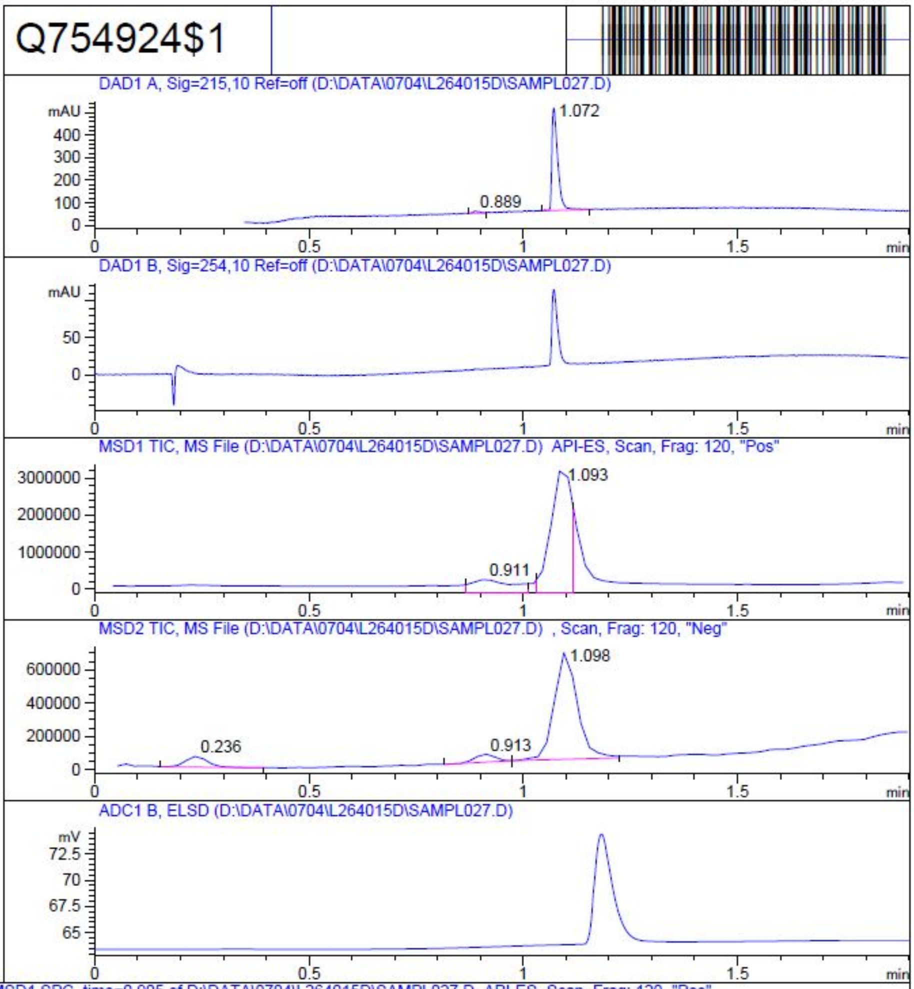

Mol Wt 451.41
Exact Mass 337.12

```
#  Time    Area%
-----------------
1  0.889    2.46
2  1.072   97.54
```

RT 0.911

RT 1.093

RT 0.236

RT 0.913

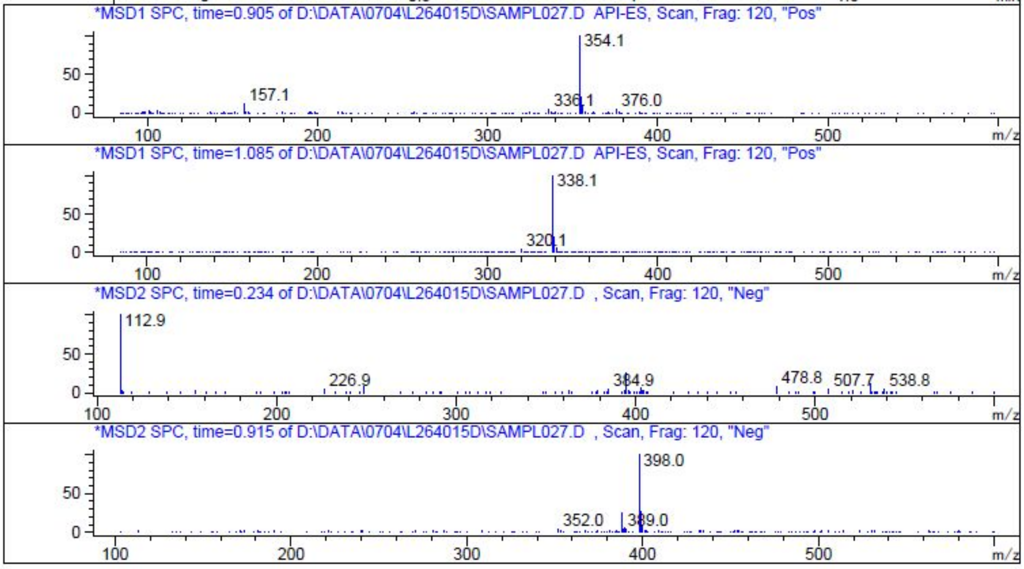

Enamine ID: Z3045122589

Q754929$5

MaxPeak: 100.00%
Ret_Time: 1.106 min

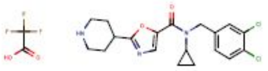

Mol Wt 508.32
Exact Mass 393.13

#	Time	Area%
1	1.106	100.00

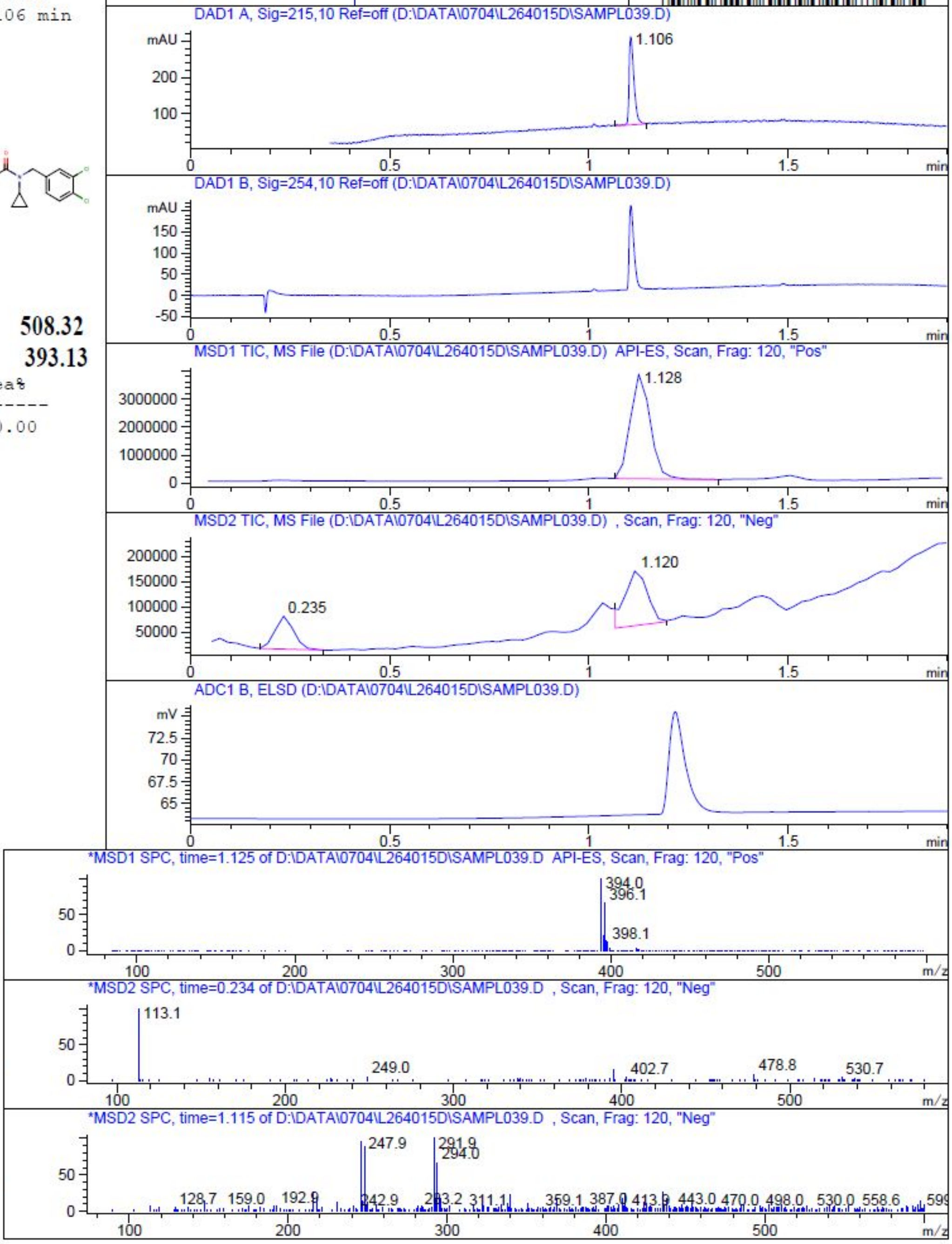

RT 1.128

RT 0.235

RT 1.120

Enamine ID: Z3100431738
